# Electrochemical Platform for Automated Design-of-Experiment Optimization of Ni-Catalyzed Cross-Electrophile Coupling Reactions

**DOI:** 10.1021/jacsau.6c01220

**Published:** 2026-09-22

**Authors:** Jieping Chen, Mathew R. Johnson, Zhifeng Tan, Tessa H. T. Myren, Kyle W. Quasdorf, Carolyn S. Wei, Shannon S. Stahl

**Affiliations:** † Department of Chemistry, 5228 University of Wisconsin−Madison, Madison, Wisconsin 53706, United States; ‡ Drug Substance Technologies - Synthetics, Process Development, 7129 Amgen Inc., One Amgen Center Drive, Thousand Oaks, California 91320, United States

**Keywords:** automated electrochemistry, electrochemical platform, nickel catalysis, cross-electrophile coupling, design of experiments (DoE)

## Abstract

Electrosynthetic organic reactions have advanced significantly in recent years, but their adoption for large-scale applications remains limited. Here, we report an automated experimental platform designed to facilitate translation of small-scale chemical and electrochemical redox reactions into robust larger-scale electrochemical processes. This platform, which integrates a commercial chemical synthesis workstation, peristaltic pumps, and a potentiostat, is equipped to conduct an automated sequence of batch experiments to optimize standard variables, such as stir rate and temperature, in addition to variables unique to electrosynthesis, such as applied current and total charge passed. Analysis of these variables using a design-of-experiment (DoE) approach accelerates identification of optimal reaction conditions and translation of mg-to-multigram operations. To evaluate the DoE optimization protocol and experimental platform, four Ni-catalyzed C­(*sp* ^2^)–C­(*sp* ^3^) cross-electrophile coupling reactions were selected from the literature that were originally conducted on a 0.2–0.4 mmol scale with chemical or electrochemical reduction methods. Each reaction was successfully scaled by a factor of 30–250, delivering up to 20 g (100 mmol) of isolated product. These results show how small-scale redox reactions may be rapidly translated to scalable electrosynthetic processes.

## Graphical abstract

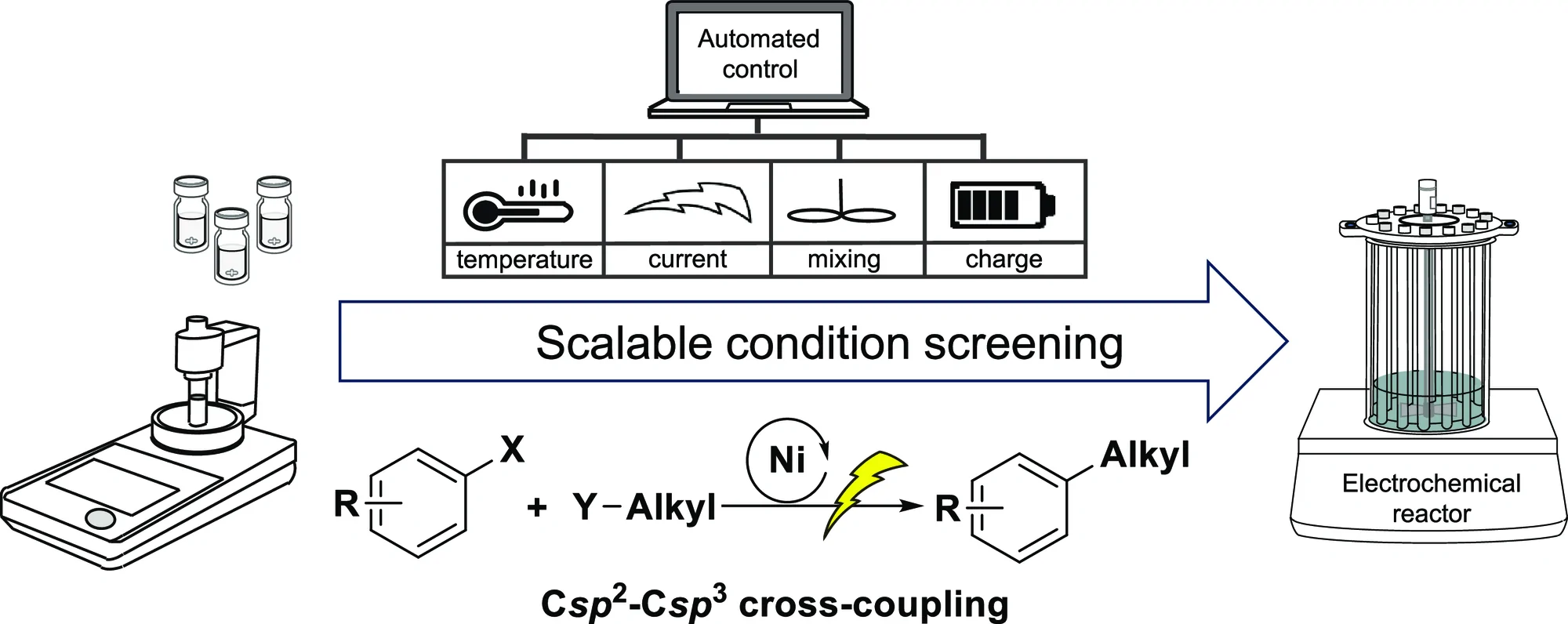

## Introduction

Electrochemistry presents unique opportunities for chemical synthesis,^1–4^ bypassing the need for conventional chemical redox reagents that can be hazardous or difficult to handle on a large scale. Such features underlie growing interest in electrosynthesis beyond lab-scale applications, particularly within the pharmaceutical industry.^5^ While redox reactions are widely used in early stages of drug discovery, their small scale places few constraints on the reagents that may be used. The safety and sustainability benefits of electrosynthesis become more evident in larger-scale reactions. Nonetheless, the rapid pace and short timelines for drug development, in addition to high compound attrition, represent barriers to the adoption of new or unfamiliar technologies as drug candidates are scaled. Such challenges motivated the present study, which originated from an interest in overcoming challenges to the use of electrochemical synthesis in early stages of drug development when small-scale (μg–mg) redox reactions identified using chemical or electrochemical methods are translated to decagrams or larger-scale applications (Figure 1a). Nickel-catalyzed cross-electrophile coupling (Ni XEC) reactions were selected as the focus of this study (Figure 1b) due to the rapidly growing use of these transformations on a laboratory scale^6^ and the interest in using electrochemistry to perform these reactions on a large scale.^5^

**Figure 1:**
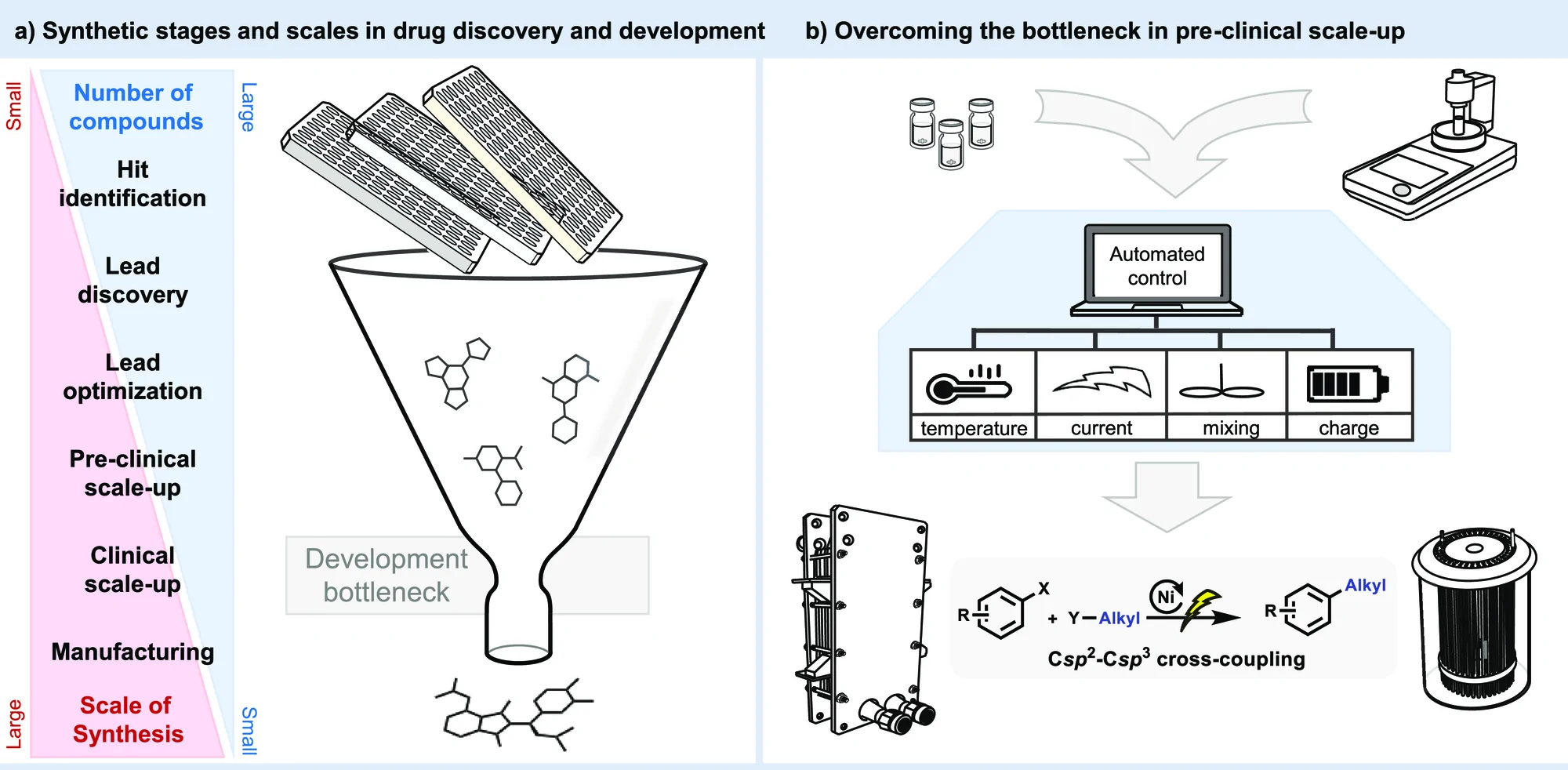
Illustrations of (a) the scale-up bottleneck encountered in the drug discovery and development pipeline and (b) automated strategy to accelerate the scale-up of pre-clinical candidates.

Translating small-scale redox reactions into a robust and scalable electrochemical process remains an active challenge. Many reactions that use chemical oxidants and reductants do not have effective electrochemical counterparts. Even when small-scale reactions are developed using electrochemistry, efforts to scale up the reaction often encounter challenges that require lengthy empirical optimization. The performance of an electrochemical reaction is controlled by multiple factors, including polarization of the working electrode and mass transport of the electroactive species within the cell, both of which are affected by inter-electrode distance, electrode geometry, and other parameters. Such features often change when moving to larger reactors or different configurations (e.g., stirred batch reactors versus parallel-plate flow reactors), and they complicate translation when scaling reactions.^7–9^ “Lack of established scale-up principles” has been identified as one of the primary roadblocks for development and adoption of electrochemical processes.^5^

Synthetic scale-up often requires optimization of multiple interdependent parameters, and design-of-experiment (DoE) methods^10,11^ provide a robust statistical framework to evaluate this complex parameter space efficiently to access an optimized outcome. Use of electrochemistry introduces additional parameters, such as the applied current/potential and total charge passed during the reaction, that influence the reaction outcome; however, continuous variables of this type should be straightforward to integrate within the standard DoE methodology.

Here, we introduce a platform to support rapid development of scalable electrosynthetic reactions. The efforts outlined herein complement other automated electrochemical reaction optimization platforms that have targeted small-scale synthetic reactions.^12–23^ The present system is designed to serve as a low-barrier entry point for non-experts in electrochemistry, integrating widely used process chemistry equipment and inexpensive electronics, controlled by custom software that supports automated DoE optimization of reaction conditions. The platform adapts an electrochemical stirred-tank reactor (eSTR) previously introduced by Harper and coworkers at AbbVie for both small- and large-scale electrochemical reactions.^24^ This reactor platform and workflow are demonstrated in the scale-up of four different Ni-catalyzed C­(*sp* ^2^)–C­(*sp* ^3^) XEC reactions identified from the literature,^25–28^ including three reactions developed using small-scale electrochemical cells and one developed as a chemical reaction with Mn powder as the reductant. The results provide a foundation for straightforward translation of new redox reactions to larger-scale electrosynthetic processes.

## Results and Discussion

### Design of the Automated Electrochemical Platform

The electrochemical platform was designed to execute a sequence of batch electrolysis operations with minimal manual intervention between runs. The primary system components (Figure 2) include a Mettler Toledo EasyMax workstation to control the temperature and overhead stirring rate; the eSTR, installed in an EasyMax port normally occupied by a standard stirred reaction vessel; a potentiostat for control of the electrolysis parameters (charge, applied current, or potential); a fraction collector to retain samples from reactions conducted in the DoE workflow; and three peristaltic pumps, one to deliver reaction solution to the reactor, a second to supply solvent to wash the reactor between experiments, and a third to transfer the reaction mixture after electrolysis to a fraction tube and the cleaning solvent following washes to a waste vessel. To ensure exclusion of air and moisture during the reaction, the reagent solution and its delivery pump, the EasyMax/eSTR assembly, and the potentiostat were maintained in a simple N_2_-purged box.

**Figure 2:**
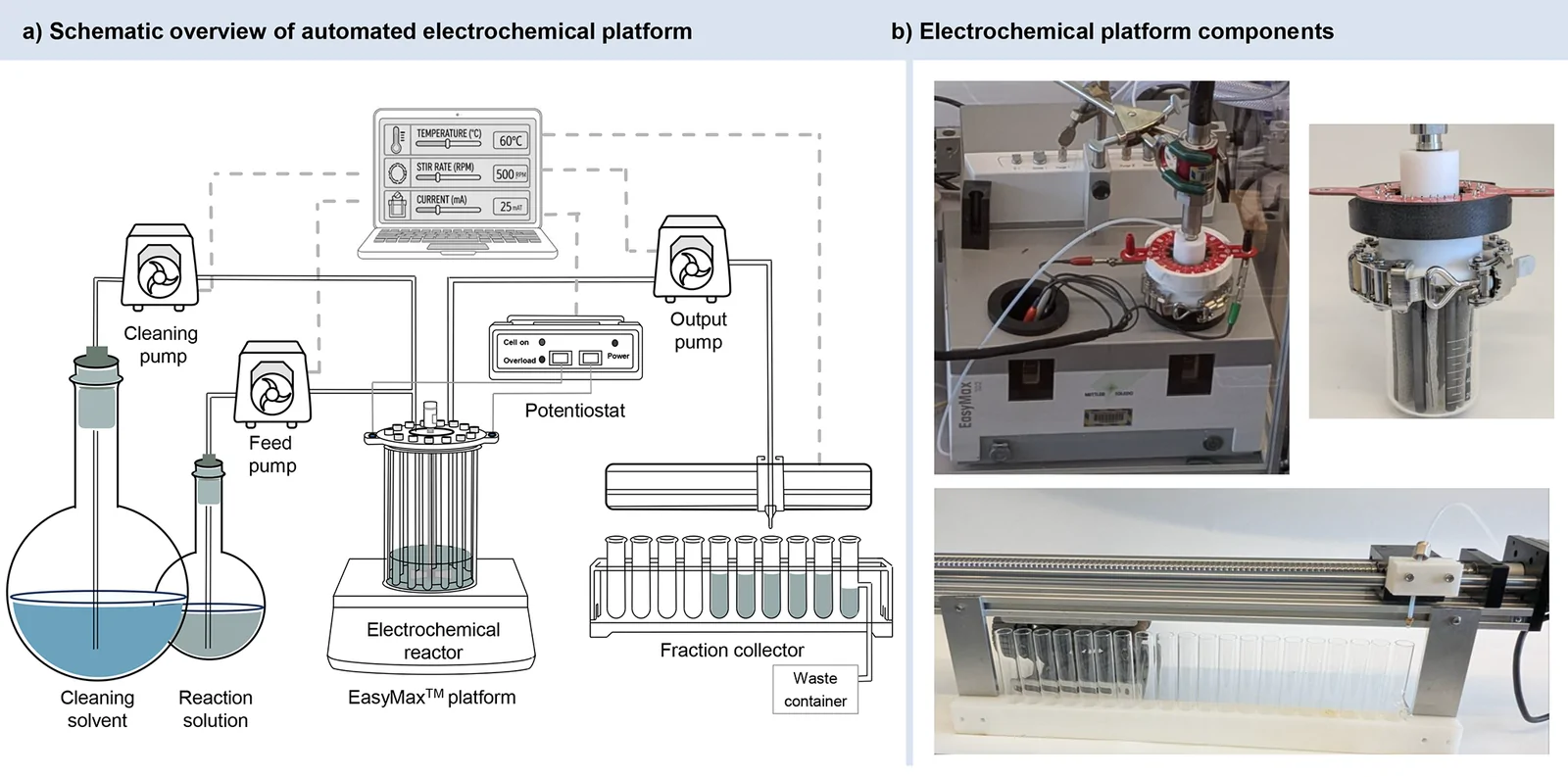
Illustration of (a) the automated electrochemical platform and (b) photographs of selected components used in this study, including the EasyMax controller, electrochemical reactor, and fraction collector.

The eSTR reactor features 14 electrode ports arranged around a central overhead stirrer, with an alternating sequence of seven anodes and seven cathodes. An annular printed circuit board was designed to simplify electrical connection to the multi-electrode eSTR through the use of spring contacts that provide a fixed interface between the potentiostat and the electrode array (see Section 2 of the Supporting Information for circuit board specifications). This design allows the electrodes to be connected and disconnected a a single unit. The more typical individual wiring of each electrode is inconvenient, time-consuming to configure, and introduces a source of error from mechanically fragile connections in addition to complications and hazards with larger reactors.

The individual hardware components were coordinated through the EasyMax interface, which managed temperature and stir rate, paired with a custom Python code and graphical user interface to manage the potentiostat, pumps, and the fraction collector.^29^ The overall reaction sequence was established by using an established DoE script^30^ to vary the desired electrolysis parameters (e.g., current, potential, charge, and electrolysis time). Once the relevant operations and time parameters were imported into EasyMax and Python software, the automated experimental workflow was initiated. The ensuing experiments featured several common steps: transfer of the reagent solution into the reactor, performance of the electrolysis under the user-defined conditions, withdrawal of the reaction solution and dispensing into a fraction tube, and a sequence of three reactor washings with a clean solvent.

This protocol enables automated DoE analysis of electrochemical conditions without physical reconfiguration of the reactor after each reaction. This approach not only minimizes manual effort but also avoids variabilities and complications that can arise from repeated mechanical disassembly/reassembly of the reactor.

### Applications

In order to test the automated electrochemical reactor platform, process operations, and DoE optimization protocol, we selected several Ni XEC reactions from the literature for development on a larger scale. A nickel-catalyzed electrochemical C­(*sp* ^2^)–C­(*sp* ^3^) reductive cross-coupling reported by Weix and coworkers was selected as an initial reaction (Figure 3a).^27^ The catalyst system features NiBr_2_ with a pair of ancillary ligands (one bipyridine and one terpyridine derivative). Substrates selected for DoE analysis were 5-bromo-2-chloropyridine (**1a**) and 1-*N*-Boc-4-bromopiperidine (**2a**). The previously reported reaction of these substrates was conducted at 70 °C on a 0.4 mmol scale. Reproduction of these conditions afforded the cross-coupled product in 62% yield (^1^H NMR), matching the isolated yield reported by Weix.^27^

**Figure 3:**
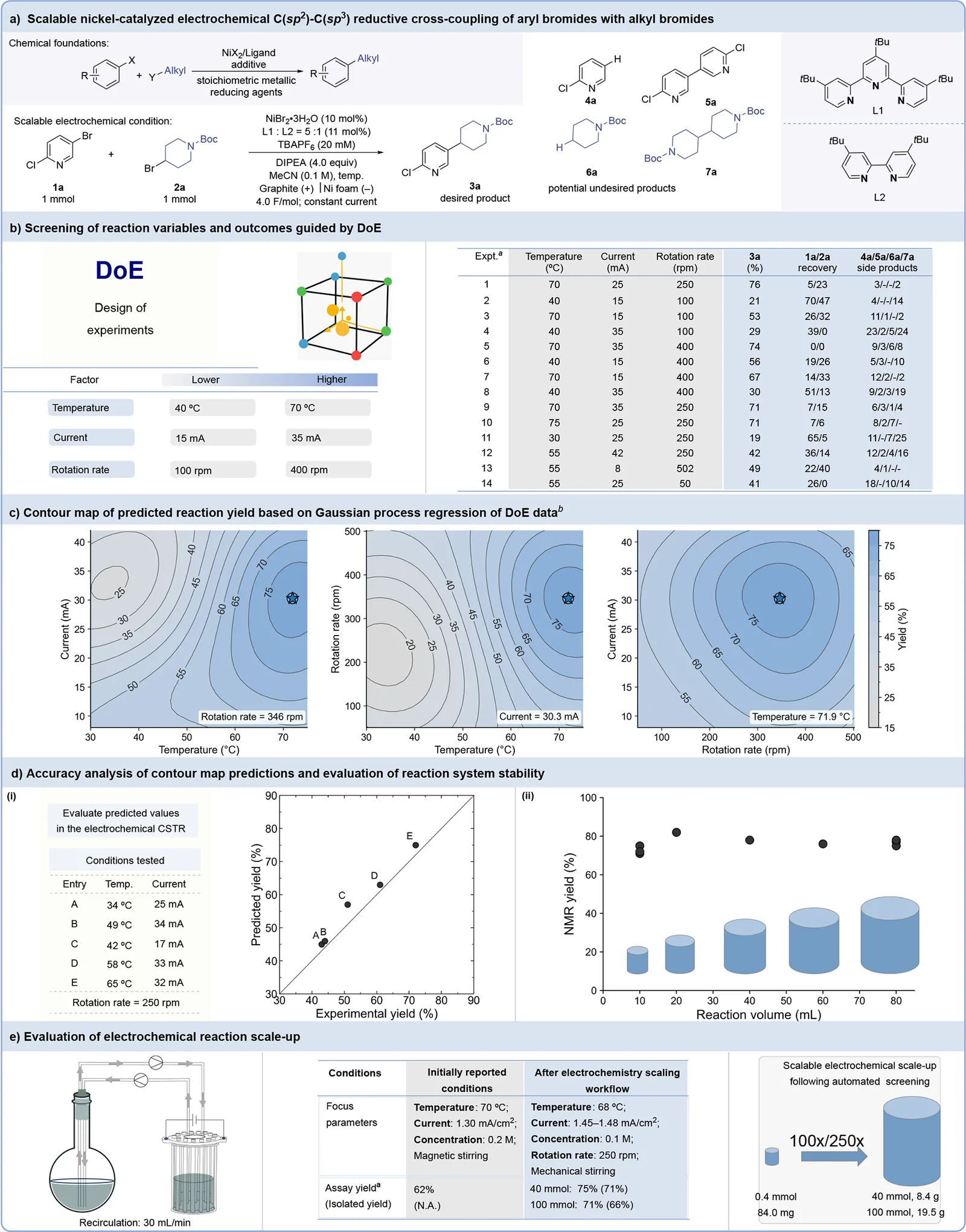
Evaluating the practicality of the designed automated electrochemical platform using a model reaction. (a) Ni-catalyzed electrochemical C­(*sp* ^2^)–C­(*sp* ^3^) cross-coupling of aryl bromide with alkyl bromide. (b) Reaction variable design via DoE and experimental data. (c) Contour map of the predicted reaction-yield landscape derived from DoE data. (d) Accuracy analysis of contour map predictions and evaluation of reaction system stability. (i): comparison between contour-predicted yields and experimental yields; (ii): variation of yield with reaction volume and trends in reaction reproducibility. (e) Evaluation of electrochemical reaction scale-up. *^a^* Assay yields determined by ^1^H NMR using 1,3,5-trimethoxybenzene as an internal standard. *^b^* Stars represent conditions corresponding to the maximum predicted yield.

The original report noted the reaction depends strongly on temperature and applied current. Consequently, we included these parameters, together with stir rate, to account for potential mass-transfer effects in the eSTR, in our initial DoE analysis (Figure 3b). The DoE parameter ranges included (i) temperature: 40–70 °C, (ii) current: 15–35 mA (corresponding to current densities of 0.77–1.79 mA/cm^2^), and (iii) rotation rate: 100–400 rpm (Figure 3b). During preliminary experiments in which each substrate was at 0.2 M, solid formation during the reaction led to blocking of the pump tubing. This complication was avoided by lowering the concentration to 0.1 M, and subsequent experiments were therefore conducted under these conditions. Aside from this change and variation of the DoE parameters, other conditions matched those of the original report: NiBr_2_·3H_2_O and ligands **L1**/**L2** (10, 9.2, and 1.8 mol %, respectively), 4 equiv of diisopropylethylamine (DIPEA) as the reductant, and 20 mM TBAPF_6_ (TBA = tetrabutylammonium) as the electrolyte, with graphite anodes and nickel foam cathodes. The programmed sequence executed 14 reactions without intervention over a 72 h time period. Samples from each reaction were collected in the fraction tube and analyzed by ^1^H NMR spectroscopy. The best result obtained from these experiments led to a 76% yield of **3a** and featured a temperature of 70 °C, a current density of 1.28 mA/cm^2^, and a stir rate of 250 rpm (Figure 3b).

The parameter-outcome relationships were visualized by using Gaussian process regression (GPR) to construct three-dimensional predictive surface plots (see Section 3 of the Supporting Information).^31,32^ The predictive two-dimensional contour maps plotting yield as a function of temperature vs current, temperature vs rotation rate, and current vs rotation rate are illustrated in Figure 3c. GPR modeling allows full prediction of yields within the selected range of parameter variables. To assess the accuracy of the model, five points within the 3D plot were randomly selected and used to evaluate the conditions experimentally on the electrochemical platform (Figure 3d-i). Comparison between predicted and experimental results gave a mean absolute error of 3.8%, an RMSE of 4%, and a maximum error of 6%, confirming the reliability of the simulations based on the GPR model. In contrast, lowering the temperature or applying higher currents led to diminished yields, accompanied by increased byproduct formation such as Het–Br hydrodehalogenation (**4a**) and alkyl radical homocoupling (**6a**). Similarly, we assessed reproducibility at different scales under near-optimal conditions identified by the GPR model: 70 °C, 1.5 mA/cm^2^ current density (the current varies with the solution volume and the surface area of the immersed electrodes), and a stir rate of 250 rpm (Figure 3d-ii). These reactions, which included three reactions at 10 mL, one reaction each at 20 mL, 40 mL, and 60 mL scale, and four at the 80 mL scale, were conducted as automated sequential reactions, similar to the DoE screening experiments. The data in Figure 3d reveal a high level of consistency across the different experiments, with yields of 71–75% for the 10 mL reactions, 76–82% for the 20–60 mL reactions, and 72–78% for the 80 mL reactions. This assay is also an effective means of determining whether irreversible reactor changes, such as electrode passivation or fouling effects, are occurring and perturbing performance. The reproducibility observed here indicates that deleterious electrode passivation has not occurred and supports the viability of electrode reuse.

In order to test the reaction performance on an even larger scale, the reaction setup was adapted to support recirculation from an external reservoir (Figure 3e). A 400 mL reaction solution (40 mmol substrate, 0.1 M) was prepared in a round-bottom flask, and two peristaltic pumps were employed to support inlet and outlet flow of the reaction mixture through the eSTR, while the other returned it from the eSTR back to the flask. The volume of the reaction mixture in the eSTR was maintained at approximately 76 mL, with a recirculating flow rate of 30 mL/min. Electrolysis was performed at a current density of 1.5 mA/cm^2^ (constant current of 150 mA) at 68 °C with a rotation rate of 250 rpm. These conditions afforded the desired product in 75% yield (71% isolated, 8.4 g). Increasing the scale further to 100 mmol afforded product **3a** in 71% yield (66% isolated, 19.5 g; see Section 3.1.17 in the Supporting Information for details). Both of these larger-scale results, obtained by using recirculating flow conditions, closely mirror the outcomes of the smaller-scale batch experiments and reinforce the robustness of the platform. More broadly, this initial demonstration validates the utility of the automated DoE protocol and reactor system to screen diverse reaction conditions, supporting reliable operation of a literature-reported Ni XEC reaction on multiple scales, including a 250-fold scale-up.

The second case study consisted of a Ni-catalyzed reductive cross-coupling reaction reported by Mei and coworkers in 2020, which features migration of Ni, converting a terminal alkyl substrate to a branched product (Figure 4a-i).^25^ We selected 4′-chloroacetophenone (**1b**) and (2-bromoethyl)­benzene (**2b**) as representative substrates to investigate this reaction. Reproduction of the original report was complicated by a lack of reported current density; however, our small-scale batch conditions led to the formation of product **3b** in 69% ^1^H NMR yield, similar to that reported (73% yield).

**Figure 4:**
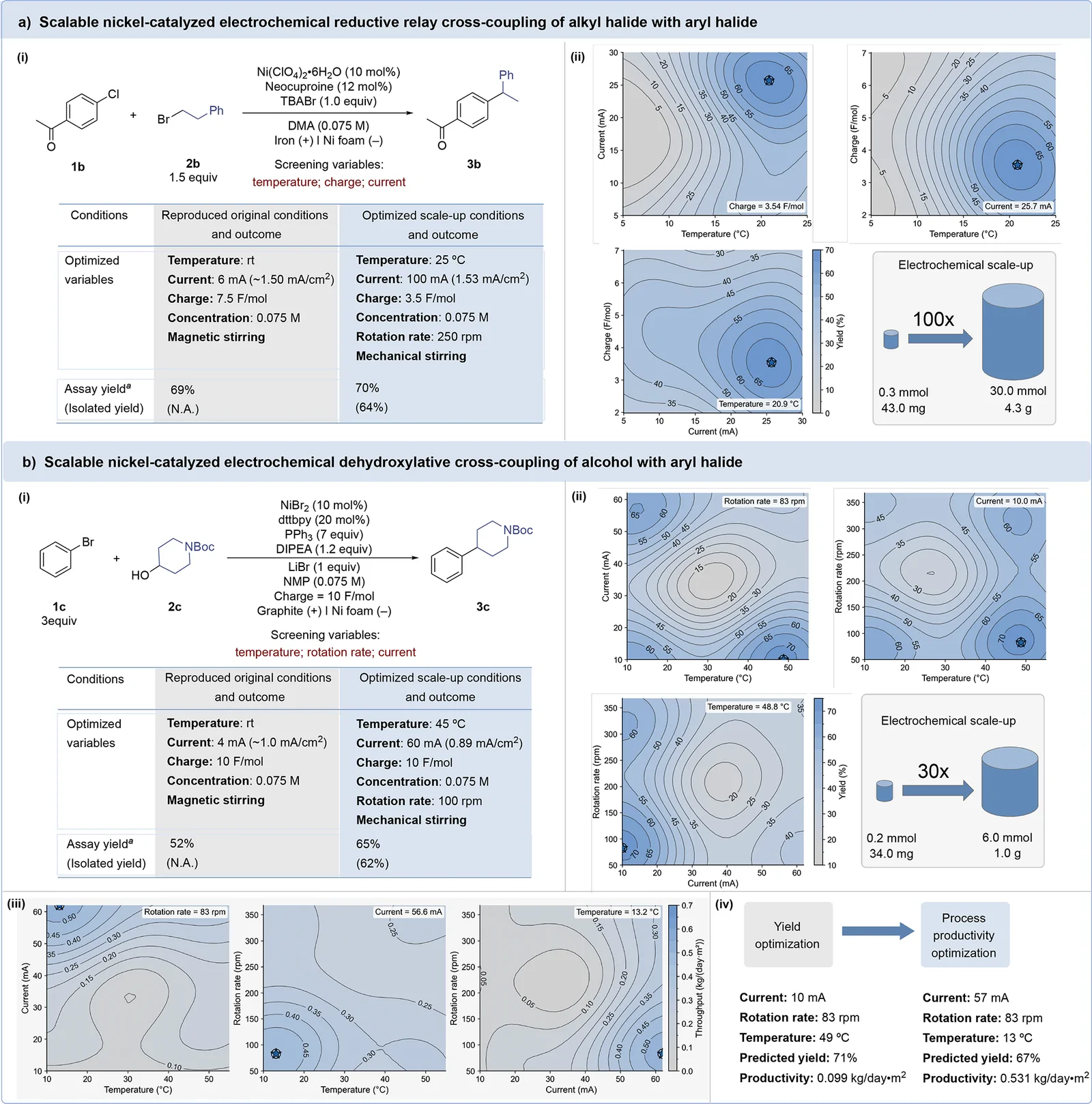
Condition optimization using an automated electrochemical rig. (a) Nickel-catalyzed electrochemical reductive relay cross-coupling of alkyl halides with aryl halides. (b) Nickel-catalyzed electrochemical dehydroxylative cross-coupling of alcohols with aryl halides.

The optimized conditions for the reported reaction featured an unusually high charge (7.5 F/mol vs a theoretical charge of 2 F/mol). The larger charge significantly prolongs the reaction time. For this reason, we opted to include “charge” among the three DoE parameters to be evaluated, supplemented with temperature and current/current density. The automated platform was then used to conduct a DoE screen, following a protocol similar to that outlined in Figure 3. The DoE parameter ranges included (i) temperature: 5–25 °C, (ii) current: 10–25 mA (corresponding to current densities of 0.60–1.49 mA/cm^2^), and (iii) charge: 3–6 F/mol. The data obtained from these experiments (see Table S11 in the Supporting Information) were subjected to GPR analysis, which was used to create the 2D contour maps shown in Figure 4a-ii. The optimal charge was found to be ∼3.5 F/mol, significantly lower than was used originally, while the optimal temperature was just below ambient conditions (∼21 °C), and the best current (and current density) was toward the top of the range evaluated: 25.7 mA (1.53 mA/cm^2^). Similar conditions were then applied to a 30 mmol scale reaction, delivering 4.3 g of the desired product in 70% yield (64% isolated yield) at a constant current density of 1.53 mA/cm^2^ with a total charge passed corresponding to 3.5 F/mol at 25 °C.

The third reaction selected, reported by Li and coworkers, is even more complex than the previous two reductive coupling reactions because it involves a paired electrolysis process (Figure 4b-i).^26^ Specifically, the alcohol is converted to an alkyl bromide at the anode prior to undergoing Ni-catalyzed coupling with aryl bromide at the cathode. The anodic reaction is proposed to proceed via in situ oxidation of bromide to bromine, Br_2_-mediated conversion of Ph_3_P to [Ph_3_PBr]­Br, alcohol activation as an alkoxyphosphonium species, and subsequent transformation into an alkyl bromide. This transformation is complicated by the need for seven equivalents of PPh_3_, multiple other additives, and in situ C–O bond activation. The reaction was evaluated with bromobenzene (**1c**) and *N*-Boc-4-hydroxypiperidine (**2c**) as model substrates. Once again, reproduction of the original report was complicated by a lack of reported current density; however, our small-scale batch conditions led to formation of product **3c** in 52% ^1^H NMR yield, similar to that reported in the literature (57% yield).

The subsequent DoE workflow was again conducted by evaluating three parameters: (i) temperature: 15–45 °C, (ii) current: 15–50 mA (corresponding to current densities of 0.89–2.97 mA/cm^2^), and (iii) rotation rate: 100–300 rpm (Figure 4b-ii). 2D contour maps constructed from GPR analysis of the DoE data (see Table S15 in the Supporting Information) show bimodal behavior, with good results appearing at high and low values for each of the three parameters. The highest yield was obtained at elevated temperature with low stirring rates and current/current density. A 6 mmol reaction conducted under conditions near the global optimum conditions for yield afforded product **3c** in 65% yield (62% isolated yield; 1.02 g). The mechanistic basis for the optimal conditions was not investigated, but the lower stir rates and current likely reflect the interplay between the rates of the redox reactions at the two electrodes in the paired electrolysis steps, the rates of the chemical reactions in solution, and mass transport of species between the electrodes. This successful translation across a 30-fold increase in scale highlights the merits of mechanism-agnostic automated DoE screening of electrolysis conditions.

The unique 2D contour plots identified for this reaction are notable because they show how the data could be used to optimize for different outputs. Most of the analysis in this study has focused on optimizing the reaction yield, but the same data could be used to optimize for other metrics, such as process productivity, reaction selectivity, or product purity, among others. This concept is illustrated in Figure 4b-iii, where the influence of current, rotation rate, and temperature are evaluated in 2D contour maps that reflect “reaction productivity”. A comparison of the resulting yield- and productivity-optimized conditions, summarized in Figure 4b-iv, illustrates that the optimal conditions may change depending on the metric of interest. The conditions identified for optimal productivity lead to a somewhat lower yield (67% vs 71%) but more than a five-fold increase in surface-area-normalized rate of product generation (0.53 vs 0.10 kg/day·m^2^, assuming 24 h/day operation).

Each of the three case studies described above featured adaptation of small-scale electrochemical reactions; however, many Ni XEC reactions reported in the literature or evaluated in laboratory-scale applications, such as medicinal chemistry, employ chemical reductants (e.g., Zn or Mn powders). Such reactions lack a direct electrochemical starting point, but larger-scale applications could benefit from electrochemical conditions. As a case study, we considered a nickel-catalyzed asymmetric reductive coupling reaction reported by Chen and coworkers in 2022,^28^ which features coupling of 5-bromo-2-(trifluoromethyl)­pyridine (**1d**) with 1-chloropropyl 4-methylbenzoate (**2d**) using Mn powder as the reductant. The original report employed a chiral biimidazoline (BiIM) ligand with 4-heptyl substituents. This ligand is not commercially available, and its preparation requires 10 linear steps.^33,34^ To facilitate our efforts here, we purchased a closely related BiIM ligand with *sec*-butyl substituents. The small-scale (0.6 mmol) results obtained with this ligand under the reported conditions with Mn powder as the reductant afforded the desired product **3d** in 56% yield and 89% *ee*, similar to the reported values of 59% yield and 94% *ee* with the 4-heptyl ligand (Figure 5).^28^

**Figure 5:**
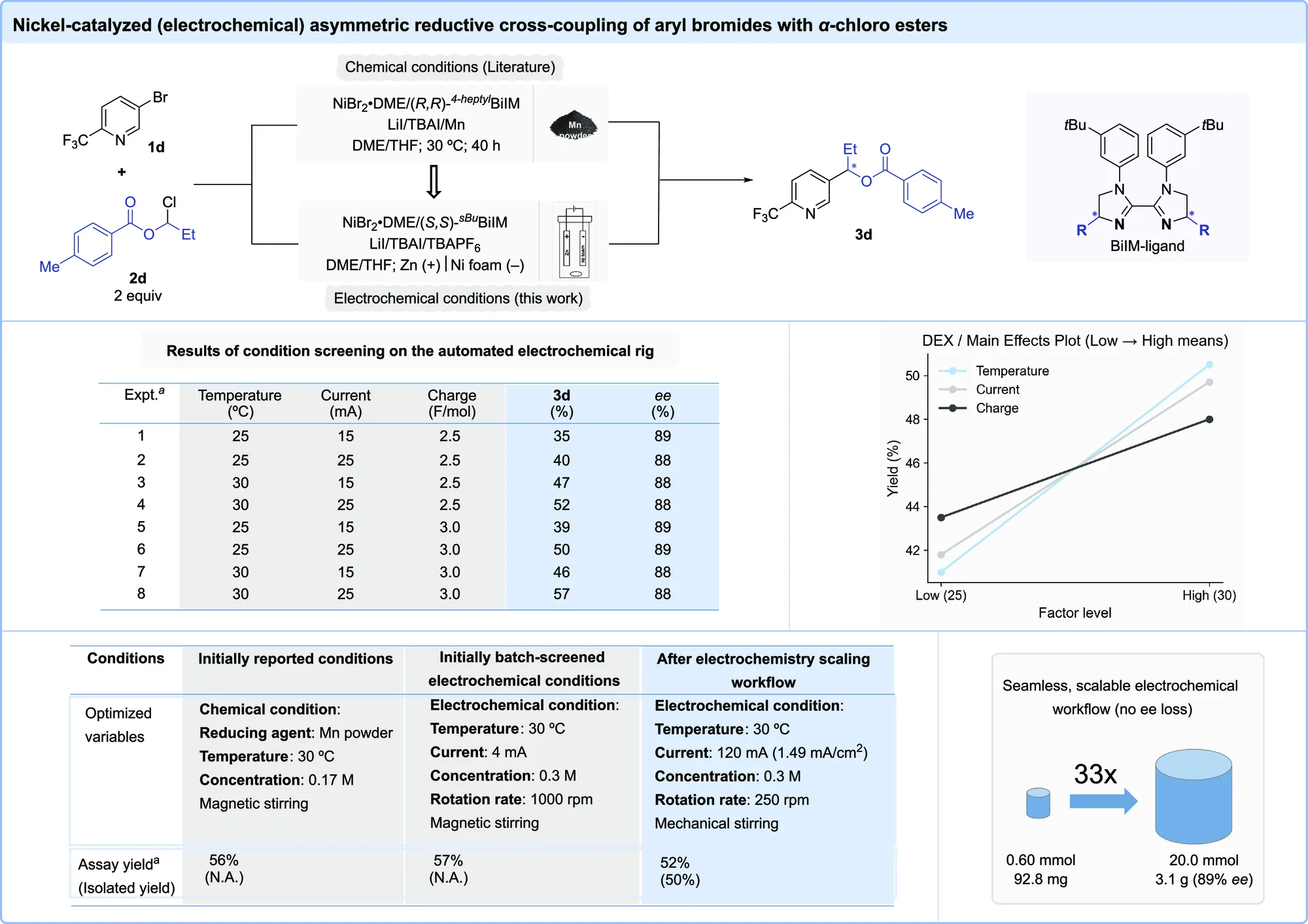
Conversion of a conventional asymmetric chemical reaction into an asymmetric electrochemical reaction. *^a^* Assay yields were determined by ^1^H NMR using 1,3,5-trimethoxybenzene as an internal standard. *ee* values were determined by chiral SFC.

Preliminary electrochemical conditions were evaluated on a small scale, starting with conditions very similar to those of the chemical conditions. A Ni foam cathode (working electrode) paired with a sacrificial Zn anode was the most effective. The low dielectric constants of the cosolvents, THF and DME, prompted the inclusion of 2.5 equiv of TBAPF_6_ as an electrolyte to enhance the solution conductivity, and improved product yield was observed at higher substrate concentrations (0.3 M **1d**). These efforts enabled the formation of **3d** in 57% yield and 89% *ee* under electrochemical conditions, providing an effective starting point for the DoE optimization.

The subsequent DoE workflow used a smaller set of conditions (eight reactions) relative to the studies outlined above due to the high cost of the ligand, but the effort still enabled optimization of the larger-scale reaction. Three DoE parameters were evaluated, including (i) temperature: 25–30 °C, (ii) current: 15–25 mA (corresponding to current densities of 0.89–1.49 mA/cm^2^), and (iii) charge: 2.5–3 F/mol (Figure 5). The automated sequence was performed sequentially across the eight conditions, and simplified DoE mean/main-effects plots were used to visualize how the reaction yield varies with temperature, charge, and current among the conditions evaluated (Figure 5). The optimized conditions (30 °C, 25 mA – corresponding to 1.5 mA/cm^2^, 3 F/mol) used in a 20 mmol-scale reaction generated the desired cross-coupled product in 52% yield and 89% *ee* (50% isolated yield; 3.09 g), reflecting a 33-fold increase in scale without erosion of *ee* relative to the small-scale chemical and electrochemical reactions.

## Conclusion

The work outlined above introduces a versatile, automated platform that facilitates the discovery of scalable electrosynthetic conditions. The apparatus minimizes manual labor and removes many of the features that can lead to variability and/or irreproducibility in experimental setup, including electrode installation and electrical connections, programmatic control over current, charge, temperature, and rotation rate, and automated reagent delivery, in situ cleaning, and product collection. The automated DoE analysis of reaction variables, including parameters such as current density and charge that are unique to electrochemistry, provides an efficient strategy to optimize the electrochemical reaction, and analysis of the data by Gaussian process regression provides a highly accessible method to identify optimal conditions. The utility of this platform has been demonstrated in four independent Ni-catalyzed C­(*sp* ^2^)–C­(*sp* ^3^) cross-coupling reactions, leading to applications on a 30–250-fold larger scale than those originally reported, with yields ranging from very similar to significantly higher than the original small-scale results. This approach minimizes scale-up effects by maintaining an identical reactor configuration across scales. The insights generated during optimization in this architecture provide a starting point for a broader systematic investigation of scaling laws, which will be necessary to translate these electrochemical transformations to larger vessels for further increases in reaction throughput and commercial relevance. Use of an eSTR reactor design that has been shown to be effective on a kilogram scale^24^ should facilitate translation to these larger scales.

Overall, this automated electrochemical reactor provides a powerful, generalizable tool to support efficient exploration of reaction space, rapid optimization of known and novel reactions, and efficient translation from microscale to preparative scale synthesis. Accessibility and ease of implementation were guiding considerations in the platform design, which uses modular, commercially available components, together with open-source control software and fabrication files. Ultimately, it would be ideal to translate this approach to a commercially available unit of integrated platform technologies that further reduces barriers to adoption.

## Supplementary Material



## Data Availability

GitHub repository: https://github.com/stahlss/2026-Electrochemical-Platform-for-Automated-Design-of-Experiment-Optimization

## Notes

Experimental details and compound characterization data (PDF)
