# Supplementary material for: Electrochemical Platform for Automated Design-of-Experiment Optimization of Ni-Catalyzed Cross-Electrophile Coupling Reactions
Source: JACS Au. 2026 Sep 22;6(10):5885–93. doi: 10.1021/jacsau.6c01220 (PMC13714832; doi:10.1021/jacsau.6c01220)
Supplement: Supplementary file 1 [file au6c01220_si_001.pdf]

Supporting Information for

# Electrochemical Platform for Automated Design-of-Experiment Optimization of Ni-catalyzed Cross-Electrophile Coupling Reactions

Jieping Chen,[1‡] Mathew R. Johnson,[1‡] Zhifeng Tan,[1] Tessa H. T. Myren,[2] Kyle W. Quasdorf,[2] Carolyn S. Wei,[2] Shannon S. Stahl*

[1]Department of Chemistry, University of Wisconsin-Madison, 1101 University Avenue, Madison, WI, 53706, United States
[2]Drug Substance Technologies - Synthetics, Process Development, Amgen Inc., One Amgen Center Drive, Thousand Oaks, California 91320, United States

Email: *stahl@chem.wisc.edu, ‡J.P.C. and M.R.J. contributed equally

## Contents

**1. General considerations ........ S3**
**1.1 Solvents and reagents ........ S3**
**1.2 Characterization of products ........ S3**
**2. Set-up and operation of the apparatus ........ S3**
**2.1 Electrochemical reactor controller user interface ........ S3**
**2.2 Design of Experiments (DoE) methodology ........ S4**
**2.2.1 Introduction to design of experiments ........ S4**
**2.2.2 Selection of the DoE design ........ S5**
**2.2.3 Experimental efficiency and reduced designs ........ S5**
**2.2.4 Selection of the number of experiments ........ S5**
**2.2.5 Data modeling and optimization ........ S6**
**2.2.6 Generation of DoE experimental data ........ S6**
**2.3 DoE post-processing template user guide ........ S7**
**2.4 Design of printed circuit board (PCB) ........ S8**
**2.5 Control of the potentiostat via the PCB ........ S9**
**2.6 Control of the peristaltic pump via the PCB ........ S10**
**2.7 Control of fraction collector via the PCB ........ S10**
**2.8 Loading data via Excel into user software, Gamry, and EasyMax™ workstation ........ S11**
**2.9 Automated electrolysis workflow and efficiency ........ S13**
**2.9.1 Flowchart illustrating the automated electrolysis process ........ S13**
**2.9.2 Efficiency comparison of automated and manual optimization ........ S13**
**2.9.3 Validation of the washing procedure ........ S14**
**2.9.4 Overcoming challenges with insolubility ........ S15**
**3. Experimental procedures for reactions on the automated electrochemical platform ........ S15**
**3.1 General procedure for optimizing the nickel-catalyzed electrochemical C($sp^2$)–C($sp^3$) reductive cross-coupling of aryl bromides and alkyl bromides ........ S15**
**3.1.1 Reproduction of the reported procedure ........ S15**
**3.1.2 Preparation of the reaction solution for condition screen ........ S16**
**3.1.3 Set-up of reaction conditions ........ S16**
**3.1.4 Set-up of stirred-tank electrochemical reactor ........ S18**
**3.1.5 Set-up of purge box ........ S19**
**3.1.6 Set-up of peristaltic pumps ........ S20**

**3.1.7 Set-up of fraction collector ........ S20**
**3.1.8 Pre-operation test of the automated electrochemical platform ........ S21**
**3.1.9 Operational procedure for automated electrochemical screening ........ S21**
**3.1.10 Results analysis ........ S22**
**3.1.11 3D representation model set-up: Predicted reaction yield of 3a ........ S23**
**3.1.12 2D contour map representation model set-up: Predicted reaction yield of 3a ........ S24**
**3.1.13 Accuracy assessment of 3D predicted data via experimental testing ........ S25**
**3.1.14 System stability evaluation using electrochemical reactions of varying volumes ........ S26**
**3.1.15 System stability evaluation in 80 mL-scale electrochemical reactions ........ S27**
**3.1.16 System stability test using electrochemical reactions of large scale (40 mmol) ........ S27**
**3.1.17 System stability test using electrochemical reactions of large scale (100 mmol) ........ S28**
**3.2 General procedure for optimizing the nickel-catalyzed electrochemical C(*sp*$^2$)–C(*sp*$^3$) reductive relay cross-coupling of alkyl halides to aryl halides ........ S29**
**3.2.1 Reproduction of the reported procedure ........ S29**
**3.2.2 Preparation of the reaction solution for condition screen ........ S29**
**3.2.3 Set-up of reaction conditions ........ S30**
**3.2.4 Results analysis ........ S32**
**3.2.5 3D representation model set-up: Predicted reaction yield of 3b ........ S32**
**3.2.6 2D contour map representation model set-up: Predicted reaction yield of 3b ........ S34**
**3.2.7 System stability test using electrochemical reactions of large scale ........ S35**
**3.3 General procedure for optimizing the nickel-catalyzed electrochemical dehydroxylative cross-coupling of alcohols with aryl halides ........ S36**
**3.3.1 Reproduction of the reported procedure ........ S36**
**3.3.2 Preparation of the reaction solution for condition screen ........ S37**
**3.3.3 Set-up of reaction conditions ........ S37**
**3.3.4 Results analysis ........ S39**
**3.3.5 3D representation model set-up: Predicted reaction yield of 3c ........ S40**
**3.3.6 2D contour map representation model set-up: Predicted reaction yield of 3c ........ S42**
**3.3.7 2D contour map representation model set-up: Predicted productivity of 3c ........ S43**
**3.3.8 System stability test using electrochemical reactions of 80 mL scale ........ S43**
**3.4 General procedure for optimizing the nickel-catalyzed electrochemical asymmetric C(*sp*$^2$)–C(*sp*$^3$) reductive cross-coupling of *α*-Cl esters and aryl bromides ........ S44**
**3.4.1 Reproduction of the reported procedure ........ S44**
**3.4.2 Batch condition screening for Ni-catalyzed electrochemical asymmetric C(*sp*$^2$)–C(*sp*$^3$) reductive cross-coupling of *α*-chloro esters with aryl bromides ........ S45**
**3.4.3 Preparation of the reaction solution for condition screen in the eSTR ........ S47**
**3.4.4 Set-up of reaction conditions ........ S48**
**3.4.5 Results analysis ........ S49**
**3.4.6 System stability test using electrochemical reactions of 80 mL scale ........ S50**
**4. References ........ S50**
**5. NMR Spectra ........ S52**

## 1. General considerations

### 1.1 Solvents and reagents

Anhydrous solvents (DMF, DMA, NMP, THF, DME) were purchased from Millipore Sigma and stored over activated 4 Å molecular sieves in a nitrogen-filled glove box. $NiBr_2·3H_2O$, $Ni(ClO_4)_2·6H_2O$, $NiBr_2$, $NiBr_2·DME$, 4,4',4''-tri-tert-butyl-2,2',6'-terpyridine, 4,4'-di-tert-butyl-2,2'-bipyridine, and neocuproine was purchased from Millipore Sigma or Thermo Scientific. BiIM-type ligand was purchased from Ambeed. Anhydrous salt additives and electrolyte (LiBr, LiI, $TBAPF_6$, TBABr, TBAI) were purchased from Millipore Sigma or Thermo Scientific. All anhydrous metal salts were stored and handled in a nitrogen-filled glove box and used as received. The other commercially available chemicals were used as received.

### 1.2 Characterization of products

All proton ($^1H$) and carbon ($^{13}C$) nuclear magnetic resonance (NMR) spectra were recorded on a Bruker Avance 400.1 MHz spectrometer at 25 ºC ($^1H$ 400.1 MHz, $^{13}C$ 100.6 MHz) using $CDCl_3$ (99.8 atoms% D) as the solvent. Chemical shifts are given in parts per million (ppm) relative to the residual solvent peak ($CHCl_3$) in the $^1H$ NMR (7.26 ppm) and $^{13}C$ NMR (77.0 ppm) spectra.

## 2. Set-up and operation of the apparatus

### 2.1 Electrochemical reactor controller user interface

A graphical user interface (GUI) was designed to control the three peristaltic pump pumps, fraction collector, and potentiostat, including their activation, deactivation, and timing functions. Screenshots of the GUI are shown below. The interface is user-friendly and enables straightforward configuration and operation of the pumps, valves, and other components of the electrochemical reactor.

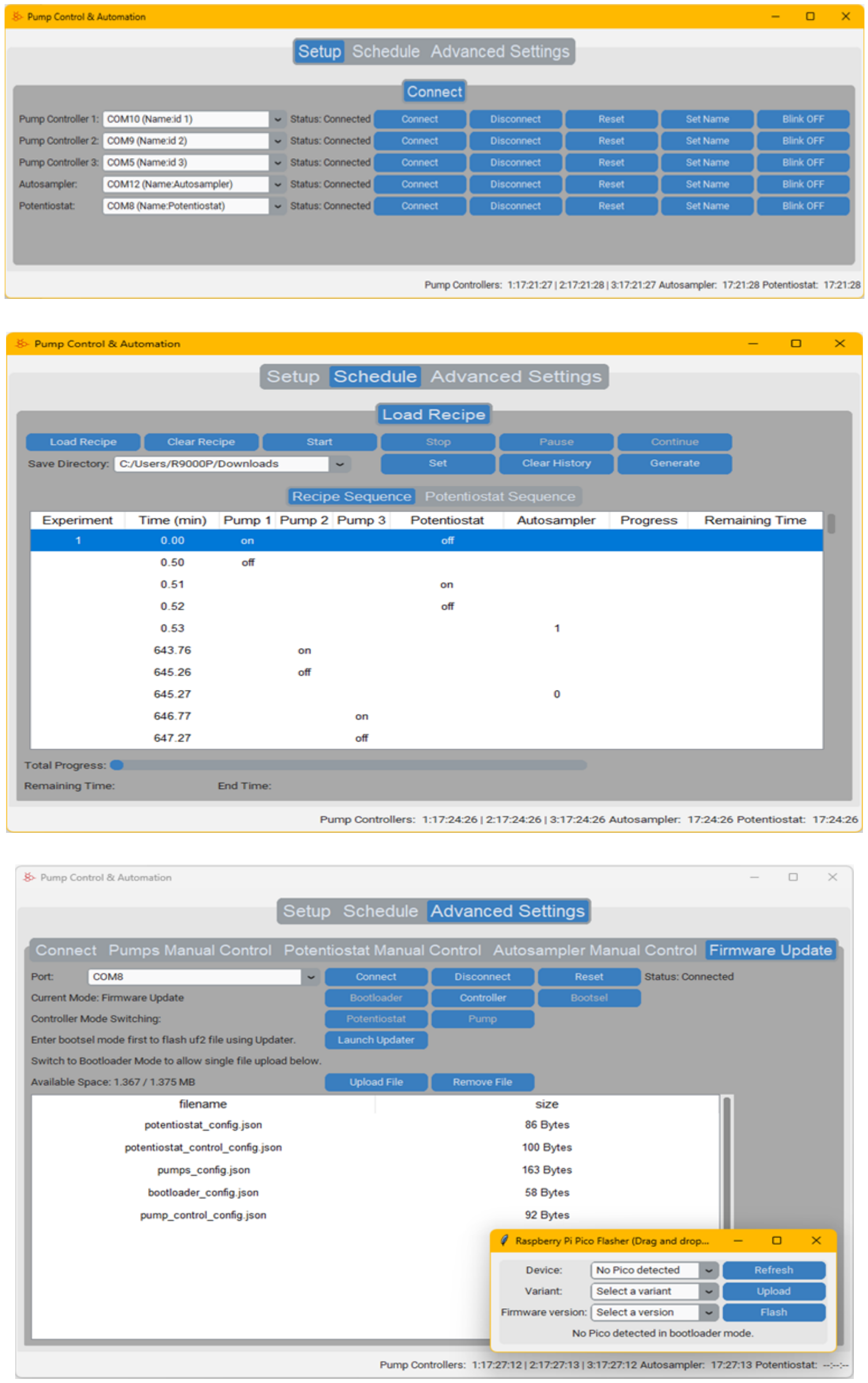


**Figure S1** Graphical user interface

**Note**: For a detailed walkthrough on designing the graphical user interface, please refer to the GitHub repository associated with this publication. The full source code for building the GUI is provided there.

## 2.2 Design of Experiments (DoE) methodology

### 2.2.1 Introduction to design of experiments

Design of Experiments (DoE) is a statistical approach that enables the systematic investigation of multiple experimental variables simultaneously. In contrast to conventional one-factor-at-a-time (OFAT)

optimization, in which individual parameters are varied independently while the others are held constant, DoE enables the evaluation of main effects, interactions between experimental variables, and nonlinear responses within a defined experimental space. This approach therefore provides an efficient means of generating multidimensional reaction data that can subsequently be used to construct predictive models and identify optimal reaction conditions.

**2.2.2 Selection of the DoE design**

A reduced Central Composite Design (CCD) approach was employed to investigate the reaction parameters in each reaction study. CCD was selected because it enables systematic exploration of multiple continuous variables and provides information on both linear and nonlinear responses within the experimental design space. In particular, the combination of factorial points, axial points, and center points enables efficient sampling of the reaction space while limiting the total number of experiments required. For each of the first three reactions studied, three reaction parameters of the four variables (current, rotation rate, temperature, and charge) were selected for optimization. These parameters were chosen based on their expected influence on electrochemical reaction performance and included reaction variables such as current/current density, charge, temperature, and/or rotation rate, depending on the specific reaction.

**2.2.3 Experimental efficiency and reduced designs**

The fourth case study, focusing on a nickel-catalyzed electrochemical asymmetric C($sp^2$)–C($sp^3$) reductive cross-coupling of $\alpha$-Cl esters and aryl bromides, provided an opportunity to investigate optimization under the constraint of limited substrate availability. Rather than a CCD-style approach, we elected to optimize using a simpler $2^3$ factorial screening, which provided effect sizes and variable interdependencies with only eight experiments. This choice maximized the information density obtained from a limited number of possible experiments, while retaining sufficient coverage of the reaction parameter space for subsequent modeling.

**2.2.4 Selection of the number of experiments**

For three experimental factors, a full quadratic response model contains ten coefficients: one intercept, three linear terms, three two-factor interaction terms, and three quadratic terms. A conventional three-factor CCD contains eight factorial points and six axial points, together with one or more center-point experiments. In the first three studies, a CCD-inspired approach was taken, in which approximately 14–15 experiments were selected for each optimization campaign to provide sufficient coverage of the three-dimensional experimental space while maintaining reasonable experimental throughput and minimizing material consumption. The selected number of experiments represents a balance between the amount of information generated and the practical cost of conducting the optimization campaign. This consideration was particularly relevant for reactions involving limited or valuable substrates and reagents.

### 2.2.5 Data modeling and optimization

The experimental data generated from the DoE campaigns were subsequently analyzed using Gaussian process regression (GPR). Rather than relying solely on a conventional quadratic response-surface model, GPR provides a flexible approach for modeling potentially nonlinear relationships between reaction parameters and reaction performance. The 14–15 experimental data points generated from the DoE therefore served as a structured initial dataset for constructing the GPR response model and identifying promising regions of the experimental space. The resulting model was then used to predict reaction conditions expected to provide improved performance. In this manner, the combination of structured DoE sampling and GPR analysis enables efficient exploration of multidimensional electrochemical reaction space while limiting the number of experiments and associated consumption of materials.

### 2.2.6 Generation of DoE experimental data

The experimental design matrices were generated using the online Design of Experiments (DoE) calculator provided by Numiqo. For each case study, a Central Composite Design (CCD) was selected, and three experimental factors and their corresponding ranges were entered into the calculator. The resulting DoE matrices were then used to define the experimental conditions for the automated optimization campaigns, as illustrated below.

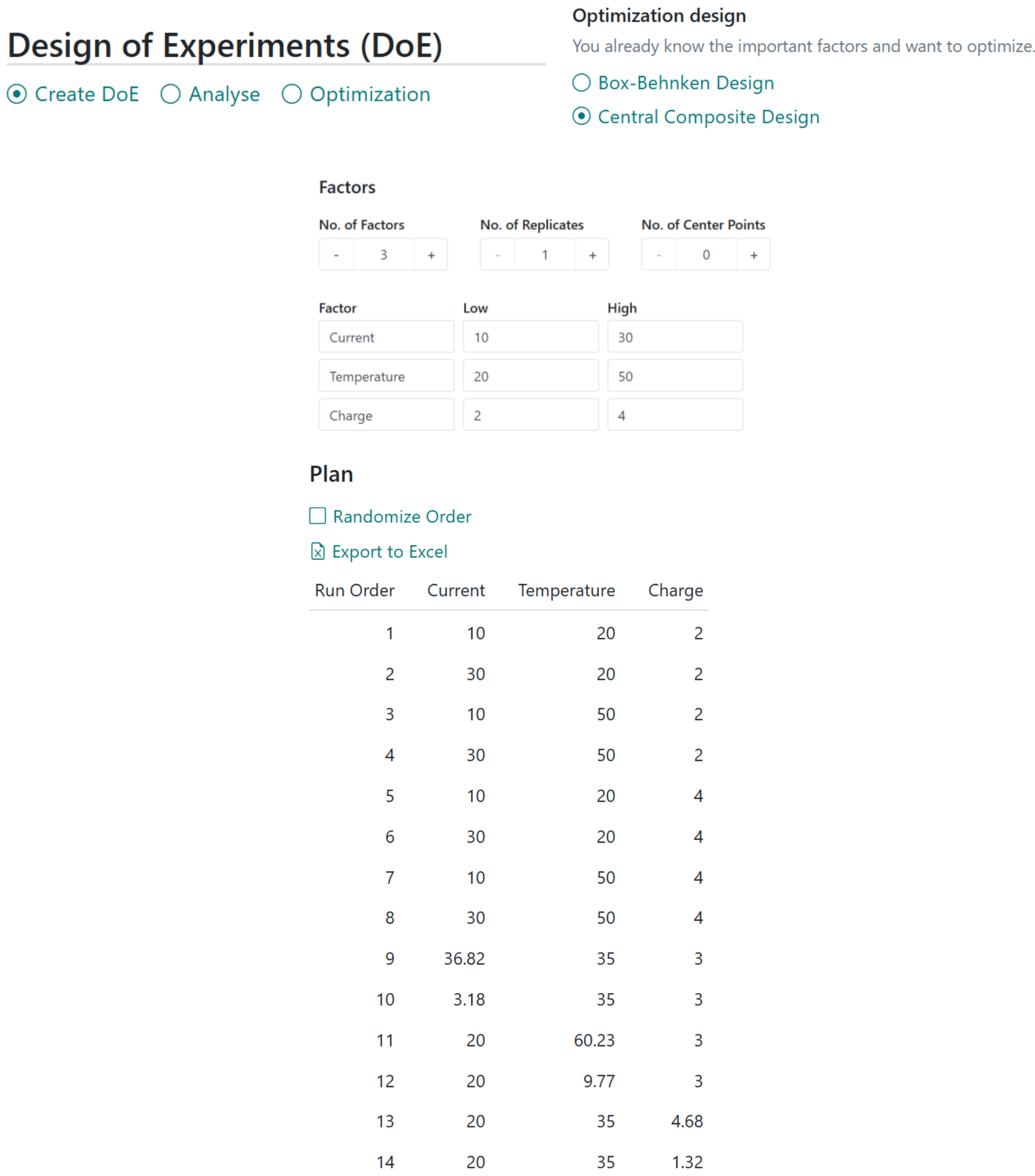


**Figure S2** Schematic illustration of theoretical DoE data generation

## 2.3 DoE post-processing template user guide

Detailed instructions and scripts for generating 3D surface plots and 2D contour maps of reaction yields are available in this publication's associated GitHub repository. Using the DoE post-processing workflow, interactive plots can be generated to visualize the relationships between reaction parameters and yields. Representative 3D surface plots and 2D contour maps generated from this workflow are shown below. Complete code and step-by-step instructions for reproducing these plots are provided in the repository.

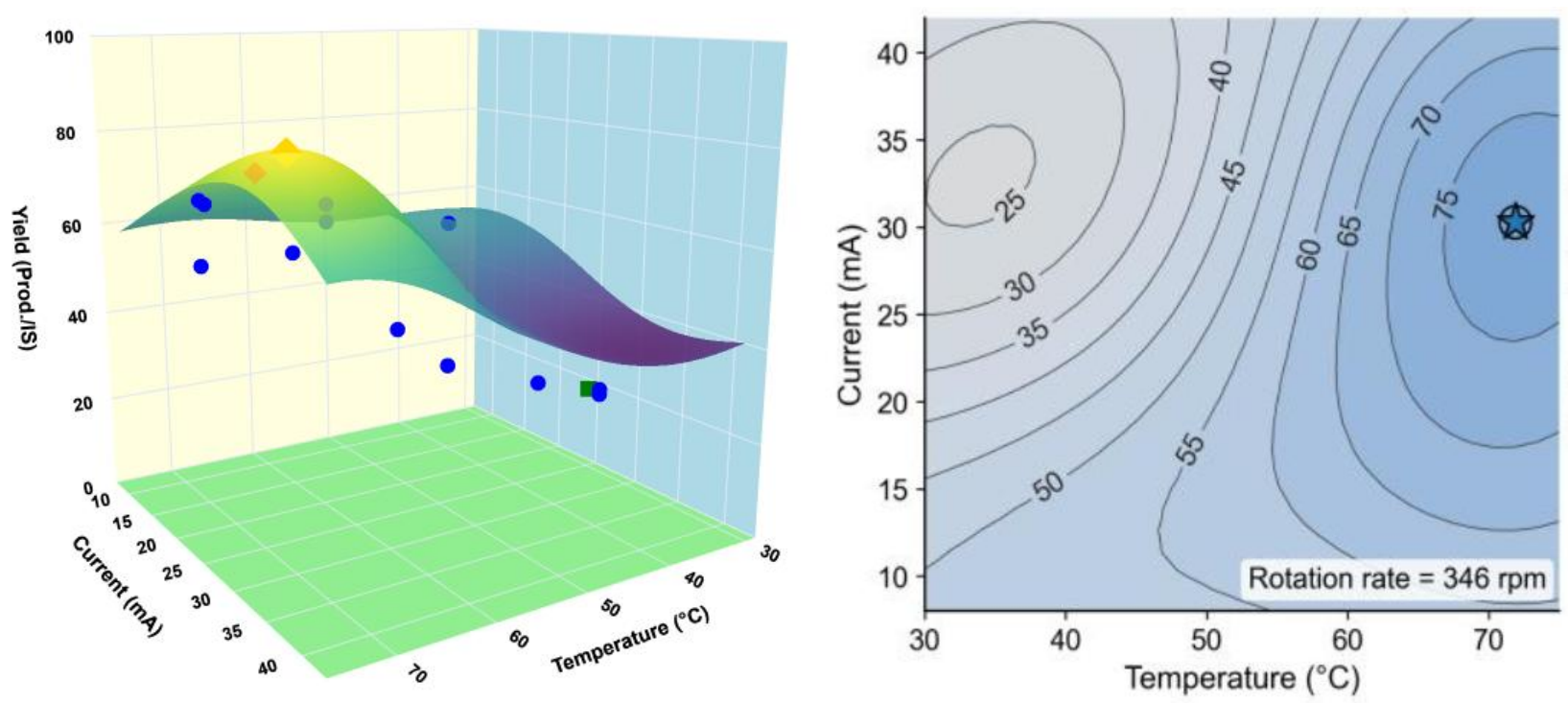


**Figure S3** Representative 3D surface plot (left); 2D contour map (right)

**Note**: For practical demonstration purposes, only the clearest and most intuitive surface plot is included in both the Supporting Information and the main text.

### 2.4 Design of printed circuit board (PCB)

We use the PCB as a physical interface to connect the experimental devices (peristaltic pump, fraction collector, and potentiostat) to the computer. It converts the control signals output from the computer into signal types that the devices can accept, and simultaneously transmits the signals measured by the devices back to the computer.

The challenge of establishing simple, reliable electrical contact to the reactor electrode array was addressed through the design of a two-layer annular printed circuit board (PCB). The PCB functions as an electrode-contact ring, providing reproducible electrical connections to the circular array of electrodes within the 100 mL stirred-tank reactor. The board geometry was constrained by the reactor headspace, the circular electrode pitch, the need for rapid assembly and disassembly, and the requirement that each electrode contact remain mechanically compliant despite small variations in electrode position, diameter, and insertion depth. Large battery-style spring contacts were selected because their broad deflection range allows reliable compression against relatively rigid electrodes without requiring precise positioning. This compliance also helps maintain contact during stirring, handling, and minor reactor vibration.

The annular PCB body was used to keep the contact interface compact while preserving access to the central reactor volume through the board's open central aperture. Electrical connection to the potentiostat was made through external tabs extending from the annulus. These tabs were intentionally placed away from the individual electrode contacts to simplify assembly and reduce the risk of accidental shorting during wiring, clipping, or handling. Circuit separation was achieved using the two copper layers of the PCB: electrodes belonging to one half-cell were connected through one copper layer, while electrodes belonging to the opposing half-cell were connected through the opposite copper layer. In this configuration, each layer functions as a mechanically integrated current-collection bus, while the insulating PCB substrate and solder

mask maintain separation between the two circuits. The final contact-ring design contained fourteen through-hole spring-contact positions arranged around the annulus, corresponding to the fourteen electrodes in the reactor array. Adjacent electrode positions were assigned to opposing half-cells, producing an alternating polarity arrangement around the ring. This layout minimizes the distance between paired anode/cathode positions in the reactor while maintaining electrical separation at the PCB level. The large copper areas used for each bus provide low-resistance current collection and avoid reliance on narrow traces, which would be mechanically fragile and unnecessarily resistive for this application. Exposed conductive regions were limited to the spring-contact footprints and external connection pads, while solder mask and geometric clearance were used to reduce incidental contact between opposing-polarity features. Overall, the PCB converts a mechanically crowded three-dimensional electrode array into a compact, reproducible, and easily assembled electrical interface fabricated using standard two-layer PCB manufacturing. The complete PCB design package, including the fabrication outputs and source design files used to manufacture the annular contact ring, is available in the associated GitHub repository.

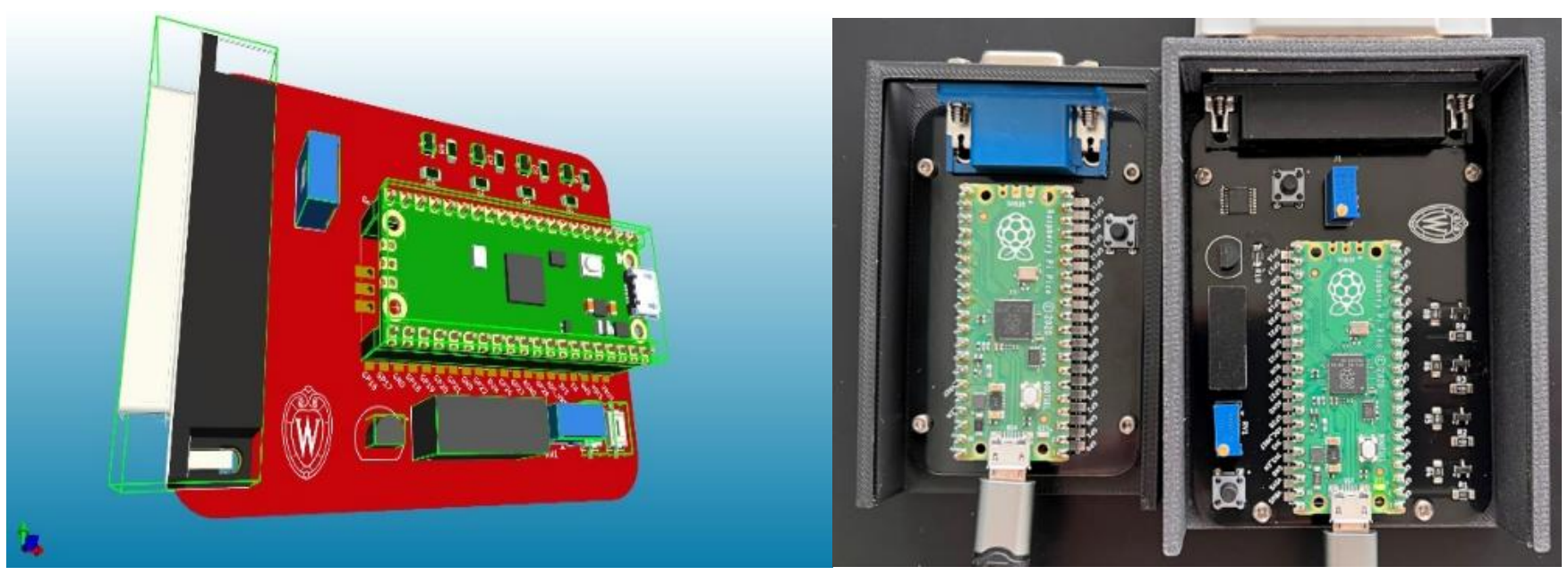

**Figure S4** Model of the PCB (left); Photograph of the assembled PCB (right)

**2.5 Control of the potentiostat via the PCB**

Using the external PCB interface, we implemented computer-controlled operation of the potentiostat. The system can deliver a specified current at a designated time—for example, during the first reaction stage of 5 hours, the specified current is applied. After the reaction ends, the potentiostat is automatically turned off, and the solution is transferred from electrochemical stirred-tank reactor (eSTR) to the designated tube in the fraction collector. Following reactor cleaning, the second reaction begins. Once the second batch of reaction solution is introduced into the reactor, the potential is instructed to turn on again, initiating the second electrolysis cycle. This process is repeated for subsequent reaction batches. For implementation details, please refer to our code repository.

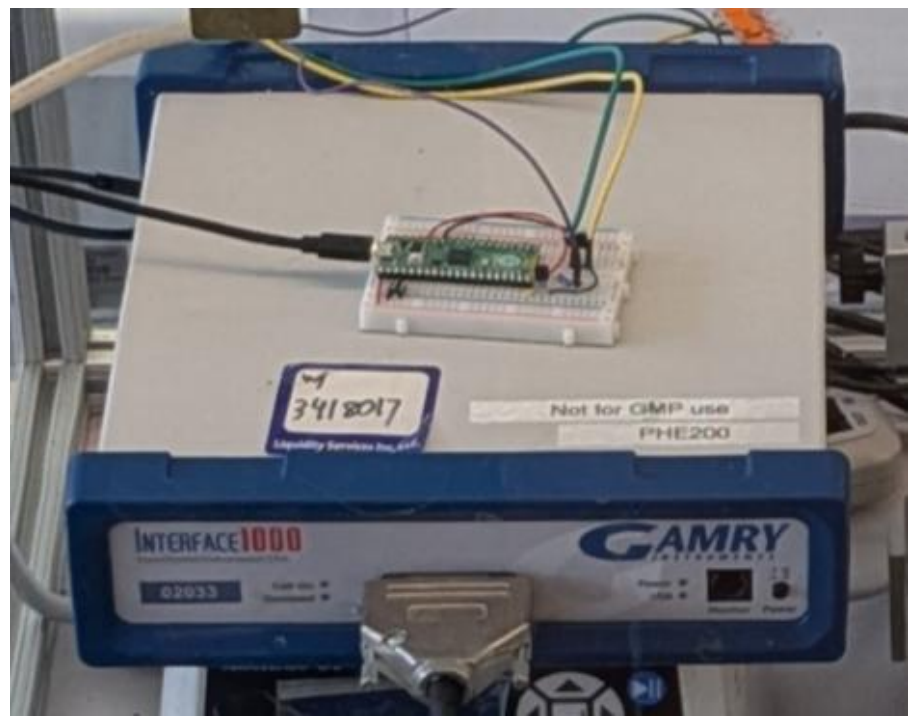


**Figure S5** Photograph of the potentiostat

### 2.6 Control of the peristaltic pump via the PCB

Using the external PCB interface, we implemented computer-controlled operation of three peristaltic pump pumps. Pump 1 (the feed pump) draws a specified volume of reactant solution into the eSTR at a designated time. After the reaction is complete, Pump 2 (the product output pump) transfers the reaction mixture from the eSTR to the designated fraction collector test tube. Pump 3 (cleaning pump) delivers the cleaning solvent—typically the same as that used in the reaction—to flush the eSTR. The waste solvent from the cleaning process is withdrawn by Pump 2 and collected in a designated waste test tube. This sequence is repeated for subsequent reaction cycles. For implementation details, please refer to our code repository.

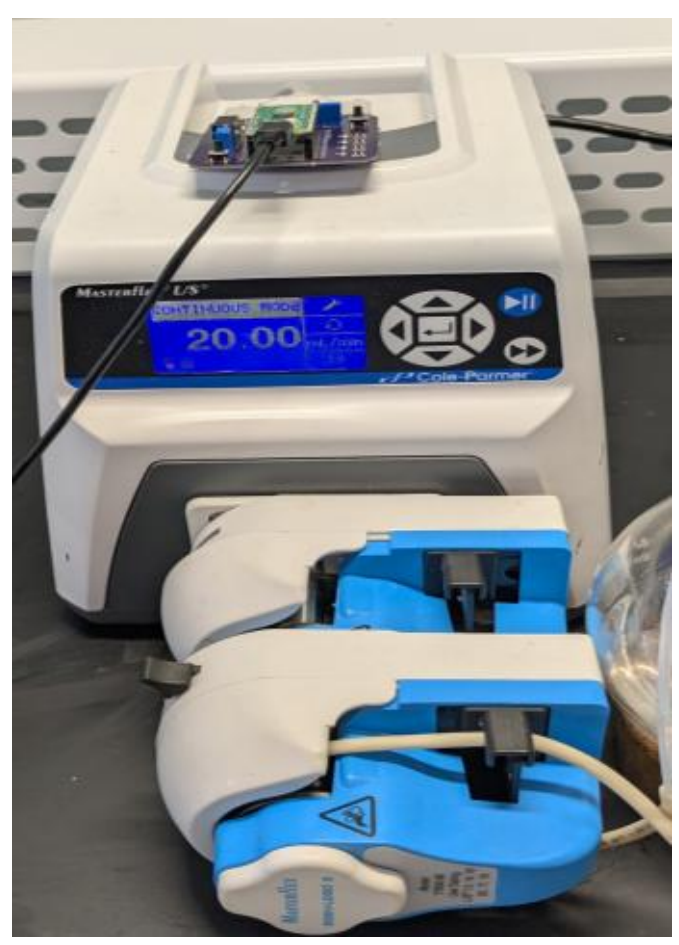


**Figure S6** Photograph of the pump

### 2.7 Control of fraction collector via the PCB

Using the external PCB interface, we implemented computer-controlled operation of the fraction collector. At the start of the first reaction, the tubing connected to Pump 2 is positioned above the first test tube. After the reaction ends, Pump 2 transfers the reaction mixture to the designated test tube. During the cleaning stage, the tubing is moved above the waste collection test tube, allowing the waste solution to be withdrawn by Pump 2 into the waste line. This sequence is repeated for subsequent reactions, with the fraction collector

moving the tubing to the corresponding test tube for each reaction cycle. For implementation details, please refer to our code repository.

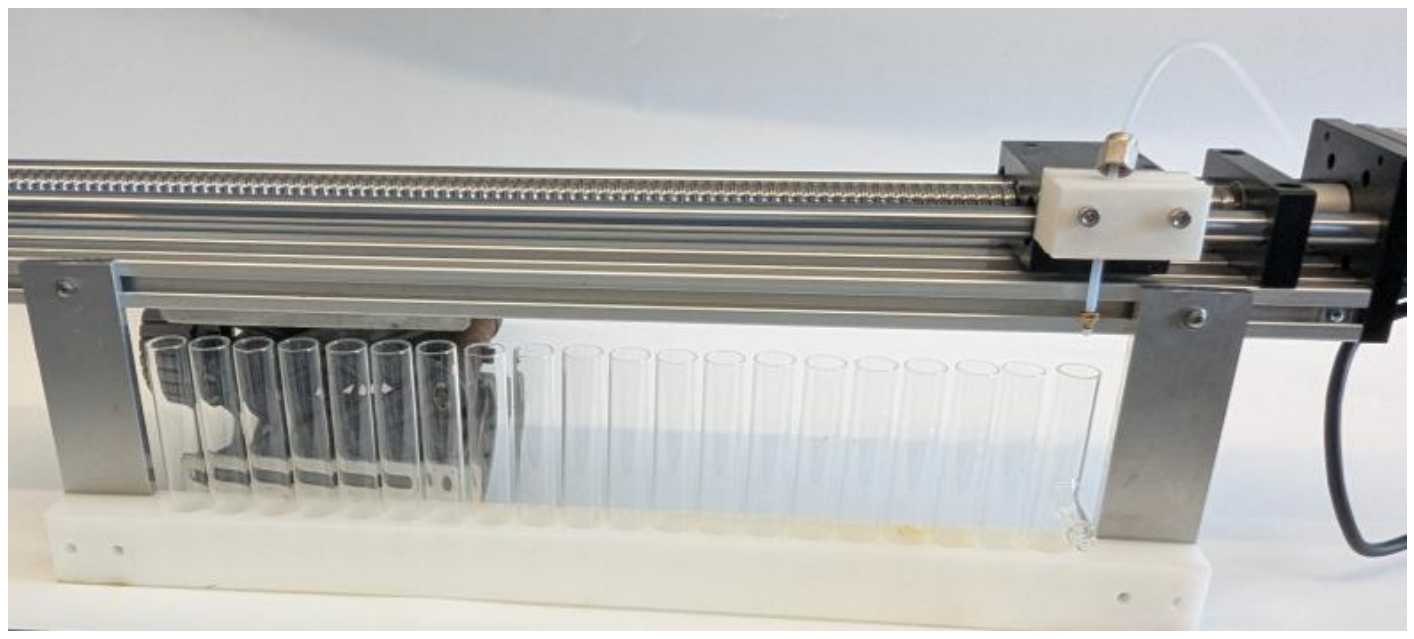

**Figure S7** Photograph of the fraction collector

**2.8 Loading data via Excel into user software, Gamry, and EasyMax™ workstation**

The computer control software reads input data via Excel. We created an Excel spreadsheet where we input parameters such as current, charge, temperature, rotation rate, reactant concentration, and reaction volume. The spreadsheet automatically calculates the reaction time. The generated data is then read by the integrated control software for the pumps, potentiostat, and fraction collector, producing corresponding Gamry-formatted files. These files can be loaded into Gamry potentiostat to control the applied potential. Additionally, the reaction temperature, rotation rate, and calculated reaction time for each reaction are input into EasyMax™ workstation. After testing that each pump, Gamry, and EasyMax™ workstation are functioning properly, the "Start" button in the GUI can be clicked, and the system automatically executes the reaction. For details on the Excel spreadsheet set-up, as well as the software installation and usage, please refer to our code repository.

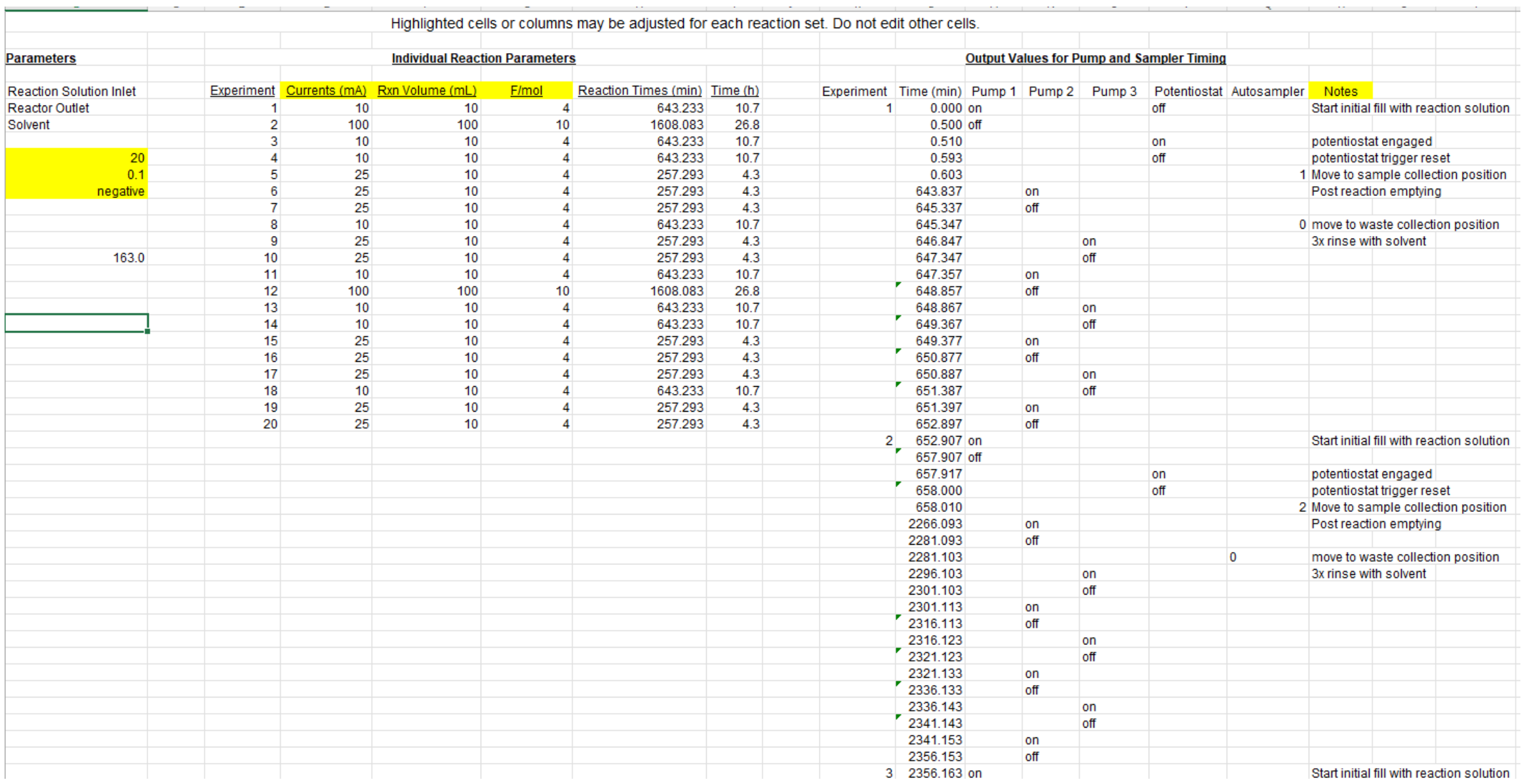

Highlighted cells or columns may be adjusted for each reaction set. Do not edit other cells.

**Parameters**

| | |
|---|---|
| Reaction Solution Inlet | |
| Reactor Outlet | |
| Solvent | |
| | |
| | 20 |
| | 0.1 |
| | negative |
| | |
| | |
| | 163.0 |

**Individual Reaction Parameters**

| Experiment | Currents (mA) | Rxn Volume (mL) | F/mol | Reaction Times (min) | Time (h) |
|---|---|---|---|---|---|
| 1 | 10 | 10 | 4 | 643.233 | 10.7 |
| 2 | 100 | 100 | 10 | 1608.083 | 26.8 |
| 3 | 10 | 10 | 4 | 643.233 | 10.7 |
| 4 | 10 | 10 | 4 | 643.233 | 10.7 |
| 5 | 25 | 10 | 4 | 257.293 | 4.3 |
| 6 | 25 | 10 | 4 | 257.293 | 4.3 |
| 7 | 25 | 10 | 4 | 257.293 | 4.3 |
| 8 | 10 | 10 | 4 | 643.233 | 10.7 |
| 9 | 25 | 10 | 4 | 257.293 | 4.3 |
| 10 | 25 | 10 | 4 | 257.293 | 4.3 |
| 11 | 10 | 10 | 4 | 643.233 | 10.7 |
| 12 | 100 | 100 | 10 | 1608.083 | 26.8 |
| 13 | 10 | 10 | 4 | 643.233 | 10.7 |
| 14 | 10 | 10 | 4 | 643.233 | 10.7 |
| 15 | 25 | 10 | 4 | 257.293 | 4.3 |
| 16 | 25 | 10 | 4 | 257.293 | 4.3 |
| 17 | 25 | 10 | 4 | 257.293 | 4.3 |
| 18 | 10 | 10 | 4 | 643.233 | 10.7 |
| 19 | 25 | 10 | 4 | 257.293 | 4.3 |
| 20 | 25 | 10 | 4 | 257.293 | 4.3 |

**Output Values for Pump and Sampler Timing**

| Experiment | Time (min) | Pump 1 | Pump 2 | Pump 3 | Potentiostat | Autosampler | Notes |
|---|---|---|---|---|---|---|---|
| 1 | 0.000 | on | | | off | | Start initial fill with reaction solution |
| | 0.500 | off | | | | | |
| | 0.510 | | | | on | | potentiostat engaged |
| | 0.593 | | | | off | | potentiostat trigger reset |
| | 0.603 | | | | | 1 | Move to sample collection position |
| | 643.837 | | on | | | | Post reaction emptying |
| | 645.337 | | off | | | | |
| | 645.347 | | | | | 0 | move to waste collection position |
| | 646.847 | | | on | | | 3x rinse with solvent |
| | 647.347 | | | off | | | |
| | 647.357 | | on | | | | |
| | 648.857 | | off | | | | |
| | 648.867 | | | on | | | |
| | 649.367 | | | off | | | |
| | 649.377 | | on | | | | |
| | 650.877 | | off | | | | |
| | 650.887 | | | on | | | |
| | 651.387 | | | off | | | |
| | 651.397 | | on | | | | |
| | 652.897 | | off | | | | |
| 2 | 652.907 | on | | | | | Start initial fill with reaction solution |
| | 657.907 | off | | | | | |
| | 657.917 | | | | on | | potentiostat engaged |
| | 658.000 | | | | off | | potentiostat trigger reset |
| | 658.010 | | | | | 2 | Move to sample collection position |
| | 2266.093 | | on | | | | Post reaction emptying |
| | 2281.093 | | off | | | | |
| | 2281.103 | | | | | 0 | move to waste collection position |
| | 2296.103 | | | on | | | 3x rinse with solvent |
| | 2301.103 | | | off | | | |
| | 2301.113 | | on | | | | |
| | 2316.113 | | off | | | | |
| | 2316.123 | | | on | | | |
| | 2321.123 | | | off | | | |
| | 2321.133 | | on | | | | |
| | 2336.133 | | off | | | | |
| | 2336.143 | | | on | | | |
| | 2341.143 | | | off | | | |
| | 2341.153 | | on | | | | |
| | 2356.153 | | off | | | | |
| 3 | 2356.163 | on | | | | | Start initial fill with reaction solution |

**Figure S8** Example screenshot of the Excel Interface

## 2.9 Automated electrolysis workflow and efficiency

### 2.9.1 Flowchart illustrating the automated electrolysis process

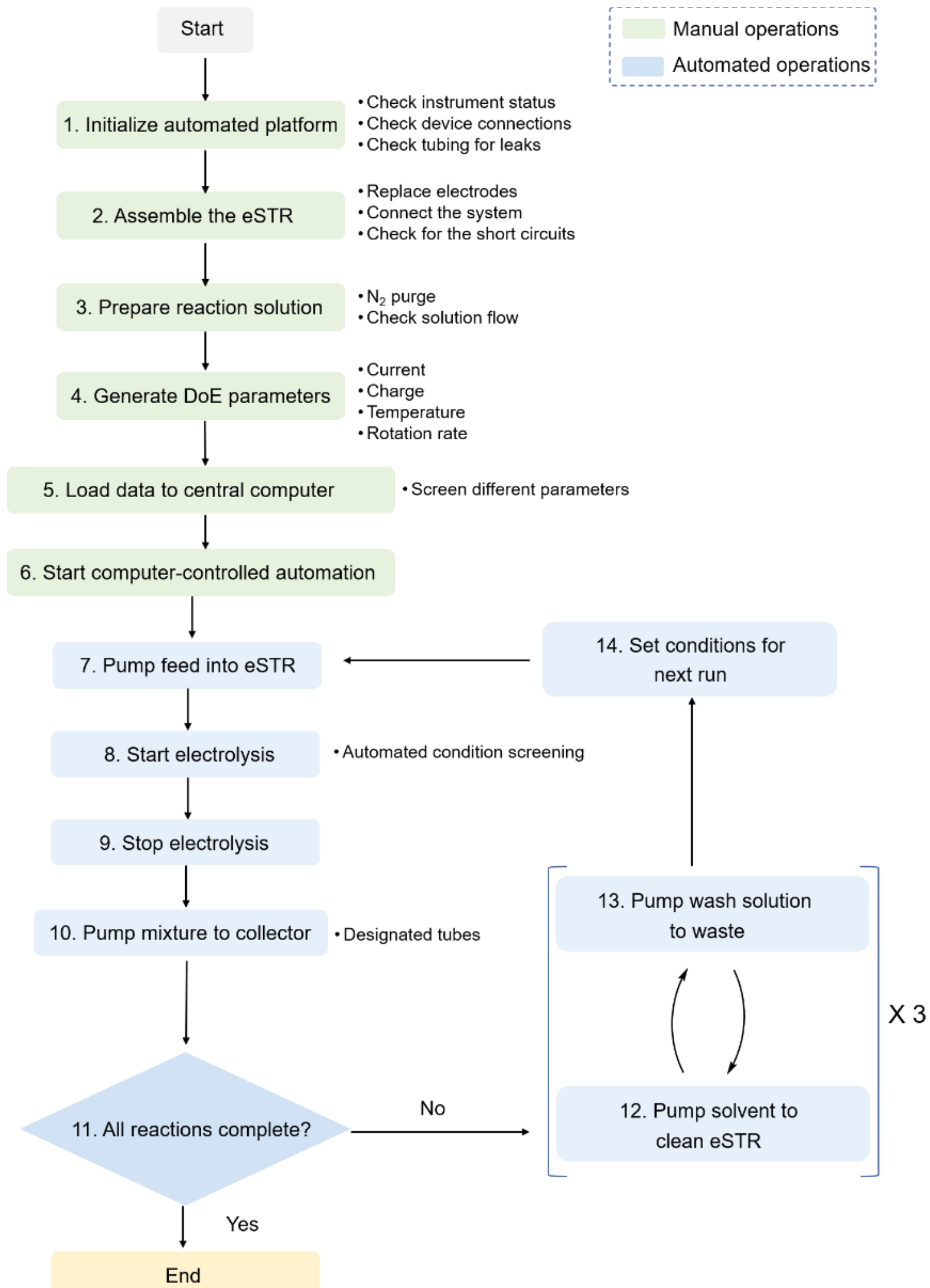


**Figure S9** Automated electrolysis workflow

### 2.9.2 Efficiency comparison of automated and manual optimization

To evaluate the practical efficiency of the automated platform, we compared the experimental workflow for a 14-run DoE campaign with that of an equivalent screening campaign conducted manually. The primary advantage of automation was a substantial reduction in repetitive experimental set-up and hands-on operation. Automation reduces the hands-on time required for a multi-experiment screening campaign by allowing the reactor and electrode assembly to be used throughout the experimental sequence. Preparation of the electrodes, including cleaning and polishing of rod electrodes and shaping and assembly of Ni foam electrodes where applicable, was estimated to require approximately 1 h for each manually conducted experiment. Reactor assembly and preparation require an additional approximately 0.5 h. Applied to the 14 experiments used in a typical DoE campaign in this study, these operations correspond

to approximately 14 h of electrode preparation and 7 h of reactor set-up, or 21 h of hands-on time. The automated campaign requires a single electrode preparation and reactor assembly, corresponding to approximately 1.5 h. Initial configuration of the pumps, fraction collector, potentiostat control, and associated software require an additional approximately 1.5 h, giving a total set-up time of approximately 3 h for the 14-experiment sequence. The automated workflow therefore reduces the estimated hands-on preparation and set-up time by 18 h, from 21 to 3 h, corresponding to an 86% reduction. This difference is equivalent to a decrease from approximately 90 min to 13 min of operator set-up time per experiment when averaged over the 14-run campaign. Following set-up, reaction initiation, product collection, reactor washing, and advancement through the programmed sequence proceed without routine operator intervention. This comparison quantifies hands-on operator time rather than total electrolysis time, which is governed primarily by the reaction conditions and is similar for the automated and manual workflows.

**Table S1** Efficiency comparison of automated and manual optimization

| Metric | Manual Optimization | Automated electrochemical platform |
|---|---|---|
| Operator intervention | Frequent | Minimal |
| Hands on time | High | Low |
| Unattended operation | Limited | Enabled |
| Experimental throughput | Low | High |
| Reproducibility | Low | High |
| Condition control | Manual | Automated |
| Parameter screening | Often one-factor-at-a-time | Multivariable DoE |
| Human error | Higher | Reduced |
| Scalability of optimization | Labor-intensive | More efficient |

### 2.9.3 Validation of the washing procedure

To assess the efficacy of solution washing steps for removing potential contaminants between reactions, a wash control experiment was performed. A 10 mL aliquot of 100 mM trimethoxybenzene solution in acetonitrile, matching the reaction volume used for batch electrochemical experiments within the study, was discharged into an electrochemical CSTR reactor assembled using 1/4" graphite and stainless steel rod electrodes. Temperature was controlled at 22 °C and the overhead stirrer was set to 300 rpm. The aliquot of solution was allowed to stir for one minute, after which the solution was withdrawn using the automated system. Three washes, each of 10 mL acetonitrile, were then sequentially introduced and withdrawn using the automated platform. Samples were taken from each effluent stream and the residual concentration of trimethoxybenzene was assessed by $^{1}$H NMR spectroscopy, with results described in Table S2 below. The results suggest significantly better than 99% substrate removal after three washes. This washing procedure was adopted as standard between each electrolysis in the optimization campaigns.

**Table S2** Residual tracer concentration after sequential washes

| Wash | [Trimethoxybenzene] (mM) | Remainder |
|---|---|---|
| 0 | 100 | 100.0% |
| 1 | 4.6 | 4.6% |
| 2 | 0.22 | 0.2% |
| 3 | < 0.05 | <0.05% |

### 2.9.4 Overcoming challenges with insolubility

Poorly soluble reaction mixtures can be accommodated through several complementary engineering strategies. In the fourth case study, the feed mixture was divided among four continuously stirred reservoirs to maintain uniformity and prevent clogging during automated transfer. Additional extensions could include temperature-controlled feed reservoirs and heat-traced tubing, recirculating feed loops, larger-bore tubing paired with slurry-tolerant pumps, and inline filtration coupled with flow or differential-pressure monitoring. These additional features were not incorporated into the present platform owing to their added cost and complexity, but they provide practical options for future applications involving low-solubility feeds.

## 3. Experimental procedures for reactions on the automated electrochemical platform

### 3.1 General procedure for optimizing the nickel-catalyzed electrochemical C($sp^2$)–C($sp^3$) reductive cross-coupling of aryl bromides and alkyl bromides

### 3.1.1 Reproduction of the reported procedure

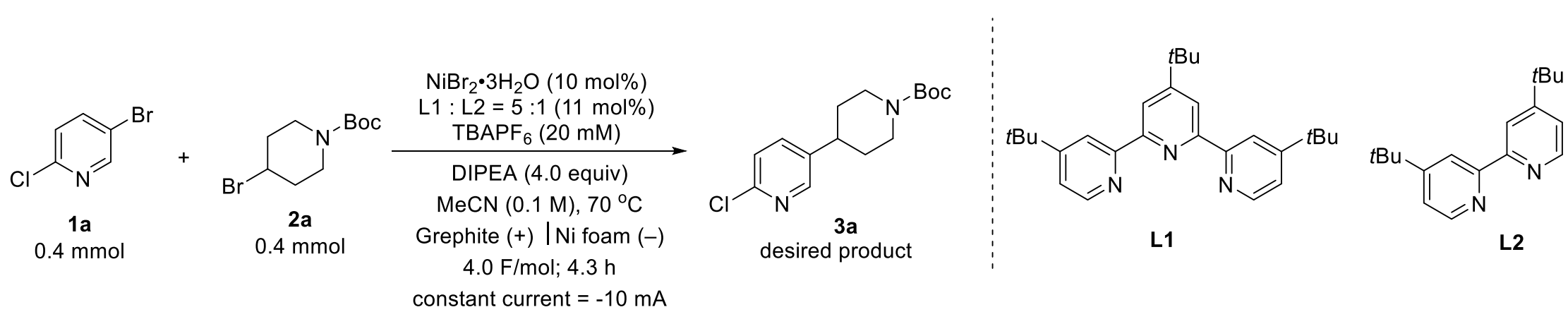


The reaction was reproduced following the reported procedure with minor modifications.[1] A test tube (16 x 100 mm) equipped with a 10 mm cross-shaped magnetic stir bar was dried overnight in an oven at 110 ºC. The hot test tube was then transferred to the glovebox antechamber, evacuated, allowed to cool to room temperature, and subsequently brought into the glovebox. $NiBr_2 \cdot 3H_2O$ (11.2 mg, 0.04 mmol, 10 mol%), **L1** (14.7 mg, 0.037 mmol, 9.2 mol%), **L2** (2.0 mg, 0.007 mmol, 1.8 mol%), and MeCN (2.0 mL) were added to an oven-dried 10 mL test tube and stirred for 10 min to afford a homogeneous emerald-green solution. $TBAPF_6$ (15.5 mg, 0.04 mmol, 10 mol%), aryl bromide **1a** (76.8 mg, 0.40 mmol, 1.0 equiv), alkyl bromide **1b** (105.6 mg, 0.40 mmol, 1.0 equiv), and DIPEA (278.7 µL, 1.6 mmol, 4.0 equiv) were then added sequentially. The reaction vessel was sealed with the electrode cap assembly. The reaction mixture was electrolyzed at 70 ºC under vigorous stirring (1500 rpm) at a constant current of −10 mA for 4.3 h using Ni foam as the cathode and graphite as the anode. Upon completion, the reaction vessel was removed from the

glovebox and quenched with $H_2O$ (200 μL). A solution of 1,3,5-trimethoxybenzene in $CHCl_3$ (100 μL, 1.0 M) was added as an internal standard, and the mixture was stirred for an additional 5 min. An aliquot (100 μL) of the reaction mixture was withdrawn and passed through a short pad of silica gel, which was subsequently washed with $CDCl_3$ (3 × 200 μL). The combined filtrates were transferred to an NMR tube, and the crude yield was determined by $^1H$ NMR spectroscopy using 1,3,5-trimethoxybenzene as the internal standard. Under these conditions, the desired product **3a** was obtained in 62% NMR yield.

### 3.1.2 Preparation of the reaction solution for condition screen

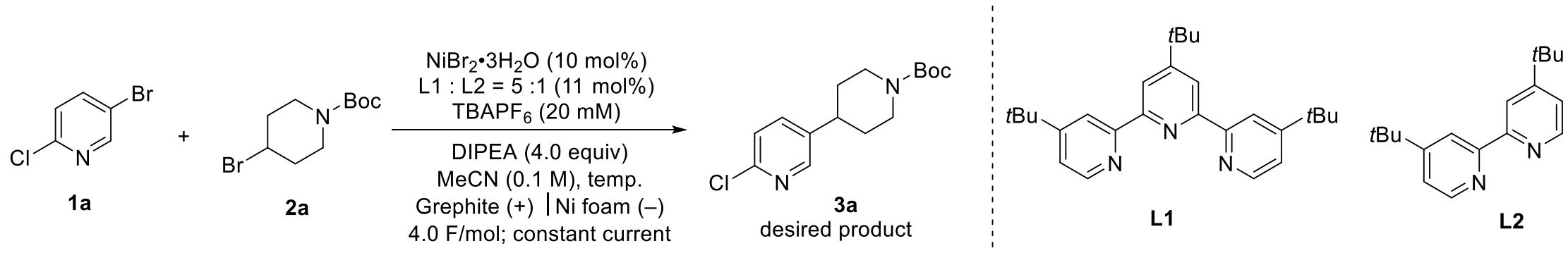


A 500 mL round-bottom flask equipped with a 20 mm cross-shaped magnetic stir bar was dried in an oven at 110 ºC overnight. The hot round-bottom flask was placed in the glove box antechamber, evacuated, and allowed to cool before being transferred into the glove box. $NiBr_2 \cdot 3H_2O$ (2.0 mmol, 542.3 mg), 4,4',4"-tri-*tert*-butyl-2,2':6',2"-terpyridine (1.83 mmol, 735.2 mg), 4,4'-di-*tert*-butyl-2,2'-bipyridine (0.37 mmol, 98.3 mg), and MeCN (0.1 M; 185 mL) were sequentially added to the flask. The resulting suspension was stirred at r.t. for 30 min to ensure full coordination of the ligands to $NiBr_2$. Subsequently, $TBAPF_6$ (4.0 mmol, 1.55 g), 5-bromo-2-chloropyridine (20.0 mmol, 3.80 g), *tert*-butyl 4-bromopiperidine-1-carboxylate (20.0 mmol, 5.28 g), 1,3,5-trimethoxybenzene (internal standard, 6.67 mmol; 1.12 g), and DIPEA (80.0 mmol, 14.0 mL) were added in sequence. The solution appeared dark gray prior to the addition of DIPEA, after which the solution turned dark green. The flask was sealed with a rubber stopper and put into the purge box (see Section 3.1.4 for description of the purge box), and a stir plate was used to mix the solution and ensure homogeneity throughout the reaction.

### 3.1.3 Set-up of reaction conditions

Three parameters—current, temperature, and rotation rate—were selected to evaluate their effect on the reaction. The ranges of these variables were chosen based on the condition screening reported in Weix's publication.[1] Design of Experiments (DoE) approach was employed to generate 14 reaction runs conditions, as summarized below. The conditions were slightly adjusted during the actual experiments to accommodate operational requirements.

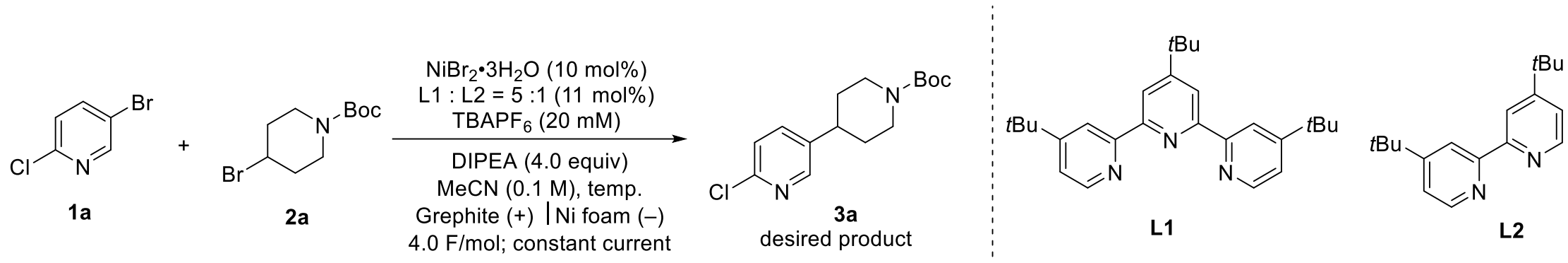


**Table S3** Variable ranges of the factors

| Factor | Lower | Higher |
|---|---|---|
| Temperature | 40 ºC | 70 ºC |
| Current | 15 mA | 35 mA |
| Rotation rate | 100 rpm | 400 rpm |

**Note**: Since the reaction is highly sensitive to both current density (Data from Weix's paper[1]: 1.3 mA/cm$^2$: 89% yield; 2.0 mA/cm$^2$: 78% yield) and temperature (70 ºC: 89% yield; 45 ºC: 35% yield), these parameters were carefully optimized. The applied current was varied from 15 to 35 mA, corresponding to a current density of 0.77–1.79 mA/cm$^2$. For reference, in a 10 mL reaction volume, the immersed geometric outer surface area of the Ni foam electrode was 19.6 cm$^2$ (W = 1.4 × 14 cm, H = 1 cm); thus, the current density was calculated as X/19.6 mA/cm$^2$. The reaction temperature was examined between 40 ºC and 70 ºC. Temperatures below 40 ºC were not explored due to limited relevance, while 70 ºC was selected as the upper limit to remain below the solvent's boiling point (81–82 ºC). The rotation rate was varied from 100 to 400 rpm. Mechanical stirring offers more efficient mass transfer than magnetic stirring; therefore, 400 rpm was chosen as the highest practical rotation rate.

**Table S4** Theoretical design of condition screening

| Run order | Temperature (°C) | Current (mA) | Rotation rate (rpm) |
|---|---|---|---|
| 1 | 40 | 15 | 100 |
| 2 | 70 | 15 | 100 |
| 3 | 40 | 35 | 100 |
| 4 | 70 | 35 | 100 |
| 5 | 40 | 15 | 400 |
| 6 | 70 | 15 | 400 |
| 7 | 40 | 35 | 400 |
| 8 | 70 | 35 | 400 |
| 9 | 80.23 | 25 | 250 |
| 10 | 29.77 | 25 | 250 |
| 11 | 55 | 41.82 | 250 |
| 12 | 55 | 8.18 | 250 |
| 13 | 55 | 25 | 502.27 |
| 14 | 55 | 25 | 0 |

**Note**: A three-factor central composite design comprising eight factorial and six axial points, without center-point replicates, was generated using NumIQO. Impractical axial conditions were adjusted before

execution. The DoE-generated table provides an organized framework for positioning data points, which facilitates the subsequent application of Gaussian process regression to construct a more accurate 3D response surface plot.

**Table S5** Modified condition screening table

| Run order | Temperature (°C) | Current (mA) | Rotation rate (rpm) |
|---|---|---|---|
| 1 | 70 | 25 | 250 |
| 2 | 40 | 15 | 100 |
| 3 | 70 | 15 | 100 |
| 4 | 40 | 35 | 100 |
| 5 | 70 | 35 | 400 |
| 6 | 40 | 15 | 400 |
| 7 | 70 | 15 | 400 |
| 8 | 40 | 35 | 400 |
| 9 | 70 | 35 | 250 |
| 10 | 80.23 | 25 | 250 |
| 11 | 29.77 | 25 | 250 |
| 12 | 55 | 42 | 250 |
| 13 | 55 | 8 | 502 |
| 14 | 55 | 25 | 50 |

**Note**: This table represents the actual screening conditions of the reaction, closely reflecting the practical experiments. It ensures that the data points are sufficiently distributed to facilitate the construction of 3D surface plot and 2D contour map.

### 3.1.4 Set-up of stirred-tank electrochemical reactor

Electrolysis was performed in a stirred tank electrochemical reactor. The reactor utilized a precedented design and was operated within a EasyMax$^{TM}$ workstation. Briefly, the reactor constituted a 100 mL glass Mettler-Toledo reaction vessel, a PTFE electrode holder, and a custom annular circuit board. Seven working electrodes (Ni foam) and seven counter electrodes (graphite) with a diameter of 0.60 cm were distributed evenly within the reaction vessel.

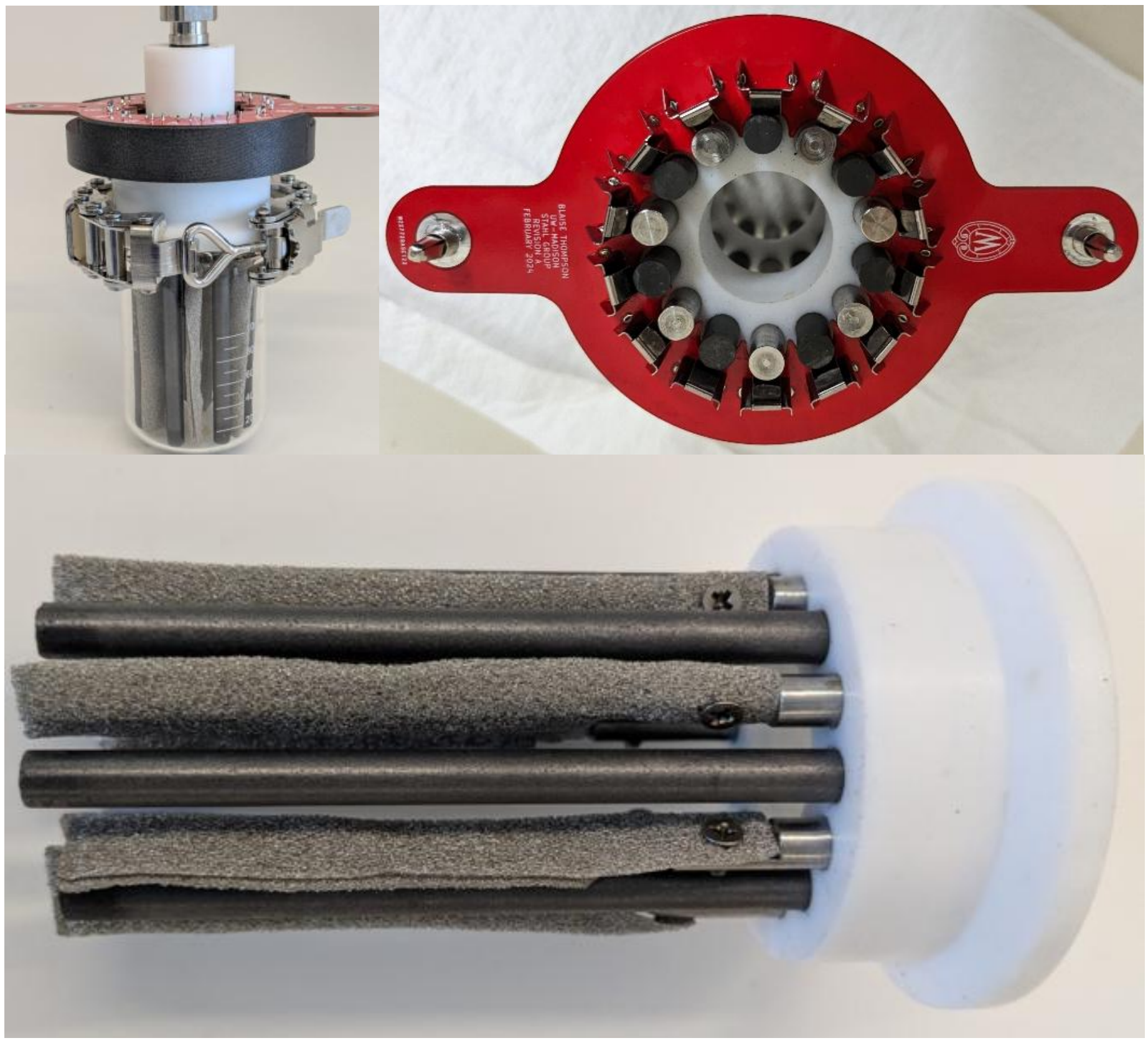

**Figure S10** Schematic diagram of the eSTR

**Note**: When Ni foam was used as the cathode, the electrode required careful fixation. Owing to the intrinsic softness of Ni foam and the short inter-electrode distance, any impact between the impeller and the electrodes (due to misalignment in the overhead stirrer) could plausibly lead to electrode shorting.

### 3.1.5 Set-up of purge box

Briefly, a purge box equipped with nitrogen inlets and tubing ports was designed. The nitrogen line was connected to the lab supply to maintain continuous purging and ensure the reaction remained under an inert atmosphere. The EasyMax$^{TM}$ workstation, a reagent delivery peristaltic pump, and the potentiostat were placed inside the box to prevent them from air, and the reactor with assembled electrodes was mounted onto the EasyMax$^{TM}$ workstation, as shown in the figure.

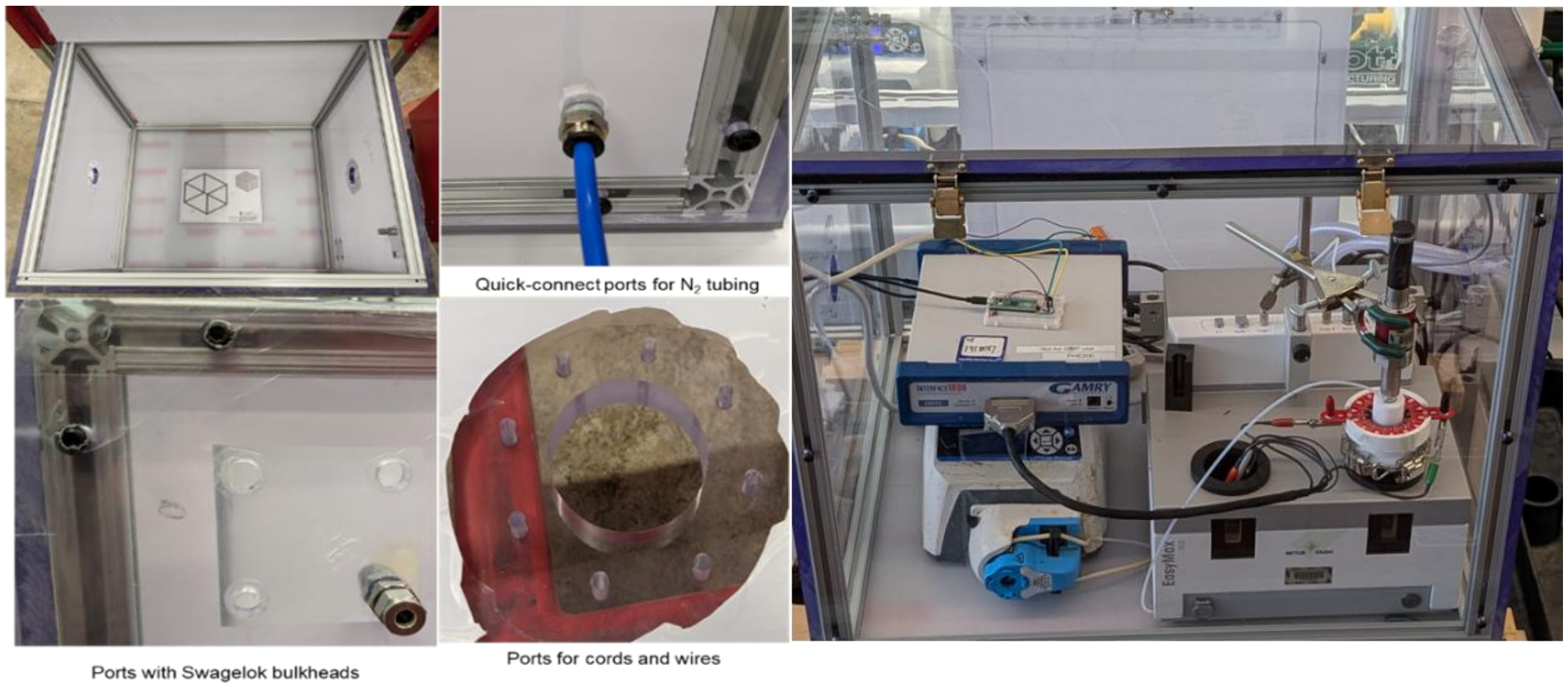


**Figure S11** Schematic purge box

### 3.1.6 Set-up of peristaltic pumps

Pump 1 (feed pump) has one end of its tubing immersed in a Schlenk flask to draw the pre-prepared solution, while the other end extends into the stirred tank. This pump is referred to as the feed pump. Pump 2 (product output pump) has one end of its tubing fixed at the bottom of the stirred tank, allowing the complete withdrawal of the crude reaction mixture after the reaction is complete. The other end is connected to the collector sampler to deliver the reaction mixture into designated test tubes. This pump is referred to as the product pump. Pump 3 (cleaning pump) has one end of its tubing immersed in a solvent reservoir used for cleaning the stirred tank, with the other end extending into the stirred tank. This pump is referred to as the cleaning pump.

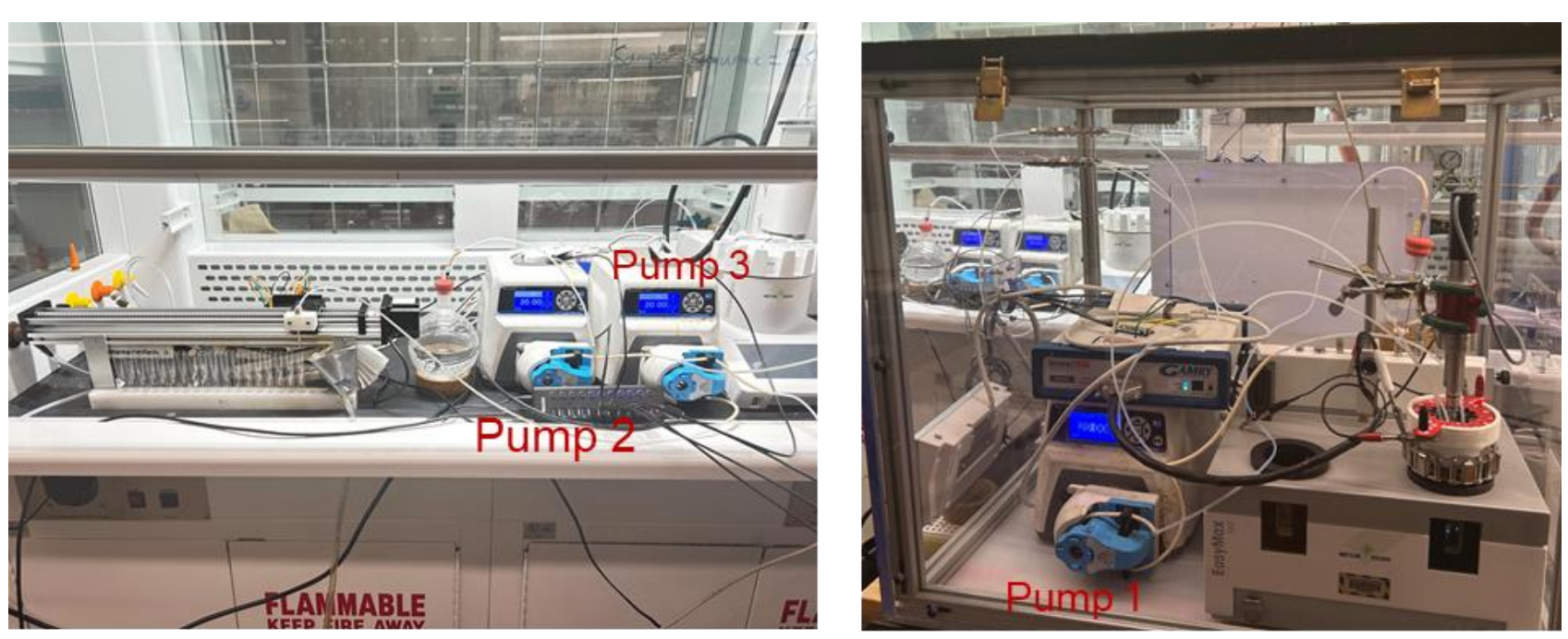


**Figure S12** Schematic pumps

**Note*:*** The rotation rate and direction of each pump were adjusted according to the specific operational requirements.

### 3.1.7 Set-up of fraction collector

The product collection module is a programmable stepper motor–driven sampling capable of controlled motion. The automated collector is first positioned at Tube 1. After the first reaction is complete, pump 2

withdraws the reaction mixture, which is collected in Tube 1. Subsequently, the stirred tank is washed three times with solvent, and the resulting waste is directed into the waste port labeled Tube 0. The collector then moves to Tube 2, and after the second reaction is complete, Pump 2 transfers the reaction mixture into Tube 2. This sequence continues in a cyclic manner until all reaction mixtures have been collected.

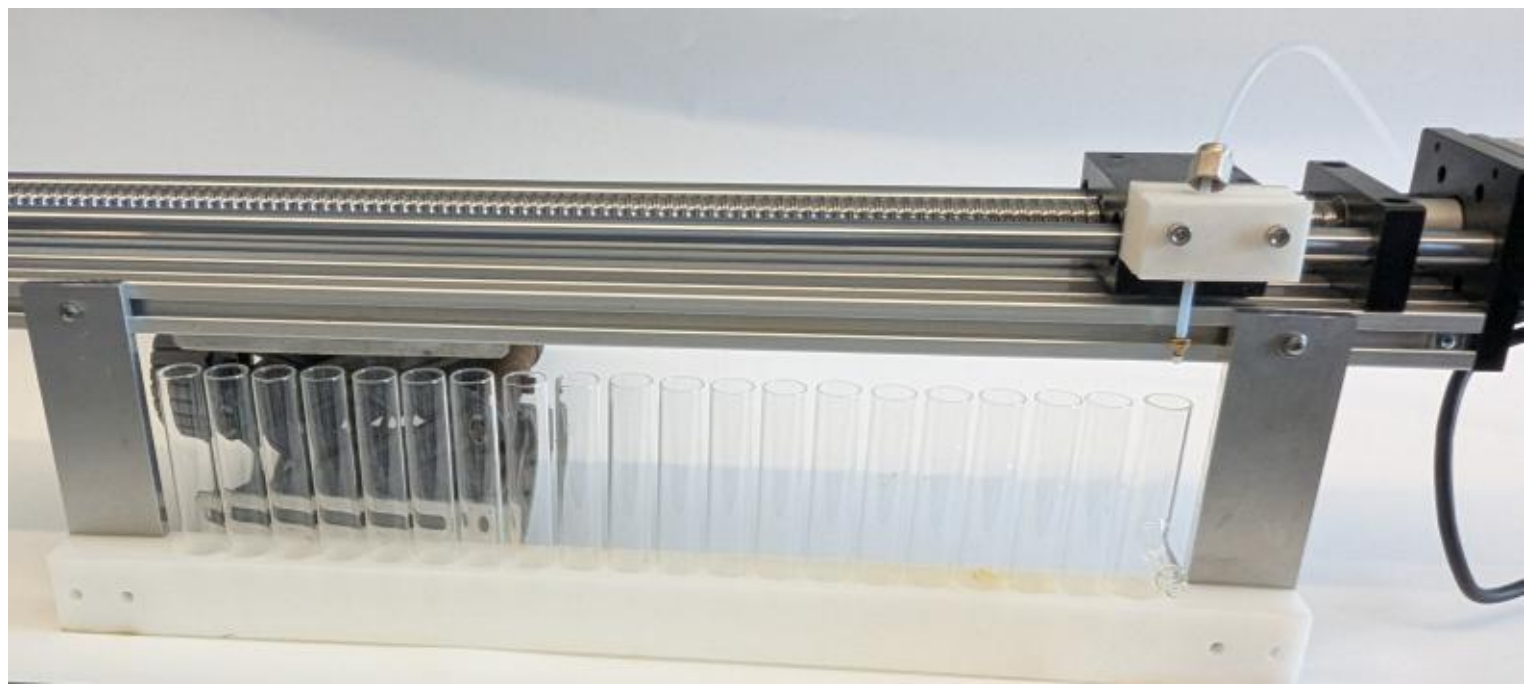

**Figure S13** Automated fraction collector

**Note:** The automated collector adjusts its travel distance based on the spacing between the two tubes, and this distance can be controlled programmatically.

### 3.1.8 Pre-operation test of the automated electrochemical platform

First, all instruments were connected to the central computer via the PCB interface, allowing the GUI to control the three pumps, the fraction collector, and the potentiostat. We verified proper operation of each component, including pump flow rate and flow direction (drawing versus dispensing), solvent/solution transfer performance, fraction collector travel distance and positioning accuracy, and potentiostat functionality (confirming no short-circuiting or abnormal behavior). After confirming that all devices responded as expected, we conducted an initial trial reaction to evaluate whether the system could operate continuously and reliably under actual reaction conditions. Because the eXEC reaction is air-sensitive, the purge box was flushed with $N_2$ for several hours prior to the reaction to ensure an inert environment.

### 3.1.9 Operational procedure for automated electrochemical screening

As described above, once all devices were verified to be properly controlled by the central computer, the prepared reaction solutions were placed inside the $N_2$-purged box, and the prepared reaction solutions was stirred using a standard magnetic stirring plate. The screening conditions were entered into an Excel sheet (see Section 2.7), which was then uploaded to the GUI to automatically generate the corresponding operational commands for initiating each reaction in sequence. At the same time, a potentiostat method file was generated and subsequently opened directly in the Gamry software to initiate electrolysis at the specified current for the designated duration. Because the central control system does not currently support automated reading or programming of the EasyMax™ workstation, the temperature, rotation rate, and reaction time for each experiment were manually entered into the EasyMax™ workstation interface prior

to starting the run. Once all devices were programmed and synchronized, the reactions were initiated, after which the system discharged the pre-set experimental sequence. No manual intervention was required during normal operation, though user oversight was provided during reactions in which solids generation was considered plausible, to ensure lack of flow interruption. In systems without this concern, fully automatic operation was observed. After all reactions were completed, the reaction mixtures were collected from the test tubes and work-up for further analysis.

### 3.1.10 Results analysis

After the reaction completed, 75 µL aliquot of the product solution was taken from the corresponding tube and passed through a short silica gel column in a pipette, eluting with $CDCl_3$ (3 × 300 µL). The resulting filtrate was then subjected to $^1H$ NMR spectroscopic analysis, and the results are shown below.

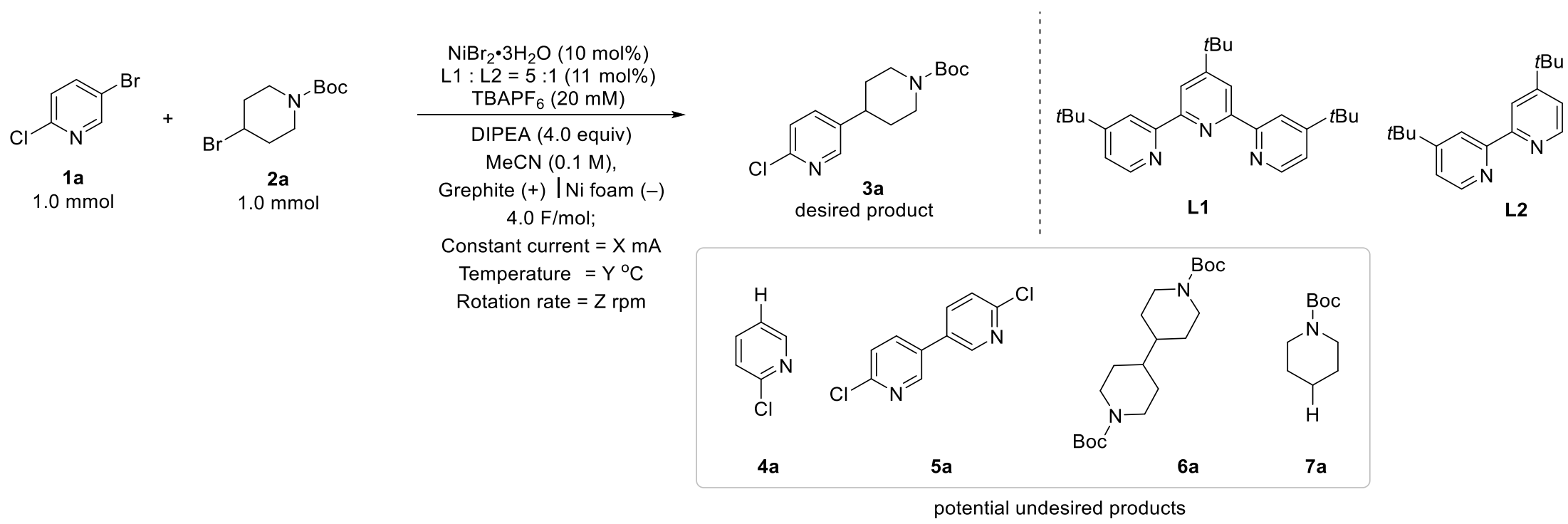


**Table S6** The crude $^1H$ NMR spectroscopic data of reaction

| Expt. | Temperature (°C) | Current (mA) | Rotation rate (rpm) | **3a** (%) | **1a/2a** recovery | **4a/5a/6a/7a** Side products |
|---|---|---|---|---|---|---|
| 1 | 70 | 25 | 250 | 76 | 5/23 | 3/-/-/2 |
| 2 | 40 | 15 | 100 | 21 | 70/47 | 4/-/-/14 |
| 3 | 70 | 15 | 100 | 53 | 26/32 | 11/1/-/2 |
| 4 | 40 | 35 | 100 | 29 | 39/0 | 23/2/5/24 |
| 5 | 70 | 35 | 400 | 74 | 0/0 | 9/3/6/8 |
| 6 | 40 | 15 | 400 | 56 | 19/26 | 5/3/-/10 |
| 7 | 70 | 15 | 400 | 67 | 14/33 | 12/2/-/2 |
| 8 | 40 | 35 | 400 | 30 | 51/13 | 9/2/3/19 |
| 9 | 70 | 35 | 250 | 71 | 7/15 | 6/3/1/4 |
| 10 | 80.23 | 25 | 250 | 71 | 7/6 | 8/2/7/- |
| 11 | 29.77 | 25 | 250 | 19 | 65/5 | 11/-/7/25 |
| 12 | 55 | 42 | 250 | 42 | 36/14 | 12/2/4/16 |
| 13 | 55 | 8 | 502 | 49 | 22/40 | 4/1/-/- |
| 14 | 55 | 25 | 50 | 41 | 26/0 | 18/-/10/14 |

**Note:** Temperature (70 °C) and lower current (25 mA) could facilitate the major transformation. Inefficient formation of **3a** leads to side reactions: Het–Br **1a** undergoes hydrodehalogenation, while alkyl–Br **2a** may

undergo homo-coupling. Balancing the yield of the desired product (**3a**) and the by-products (**4a–5a**) is critical, as these two pathways compete with each other.

### 3.1.11 3D representation model set-up: Predicted reaction yield of 3a

Reaction yields across the explored parameter space were predicted using Gaussian process regression and visualized in a 3D plot. Gaussian process regression was used to generate approximately 20,000 predicted data points based on 14 sets of experimental data. The predicted data were then used to construct the corresponding predicted plot. The code is provided in the accompanying file (3D_plot_code.ipynb). Points on the surface represent the variation in yield with respect to two parameters while the third parameter is held at its optimal value, providing insight into yield trends across the examined variable ranges. By integrating the electrochemical set-up with 3D visualization, we can predict reaction yields and optimize the condition screening process, thereby minimizing unnecessary experimental efforts. A 3D model was constructed based on data obtained from the DoE screening to analyze and predict trends in reaction yield. The model explores the relationships between pairs of key variables, including current and temperature, current and rotation rate, and temperature and rotation rate. As shown in the 3D plot, the reaction is particularly sensitive to current and temperature. Yields decrease when the current is too low or too high, or when the temperature is too low. The decline at high temperatures may be due to solvent evaporation near its boiling point, which alters concentrations and affects the reaction, while low temperatures may slow the rate of nickel oxidative addition procedure. An intermediate current density optimizes the reduction rate, maximizing the reaction yield. In contrast, the rotation rate has a relatively minor effect, likely because the mechanical stirring employed already provides sufficient agitation to enhance mass transfer.

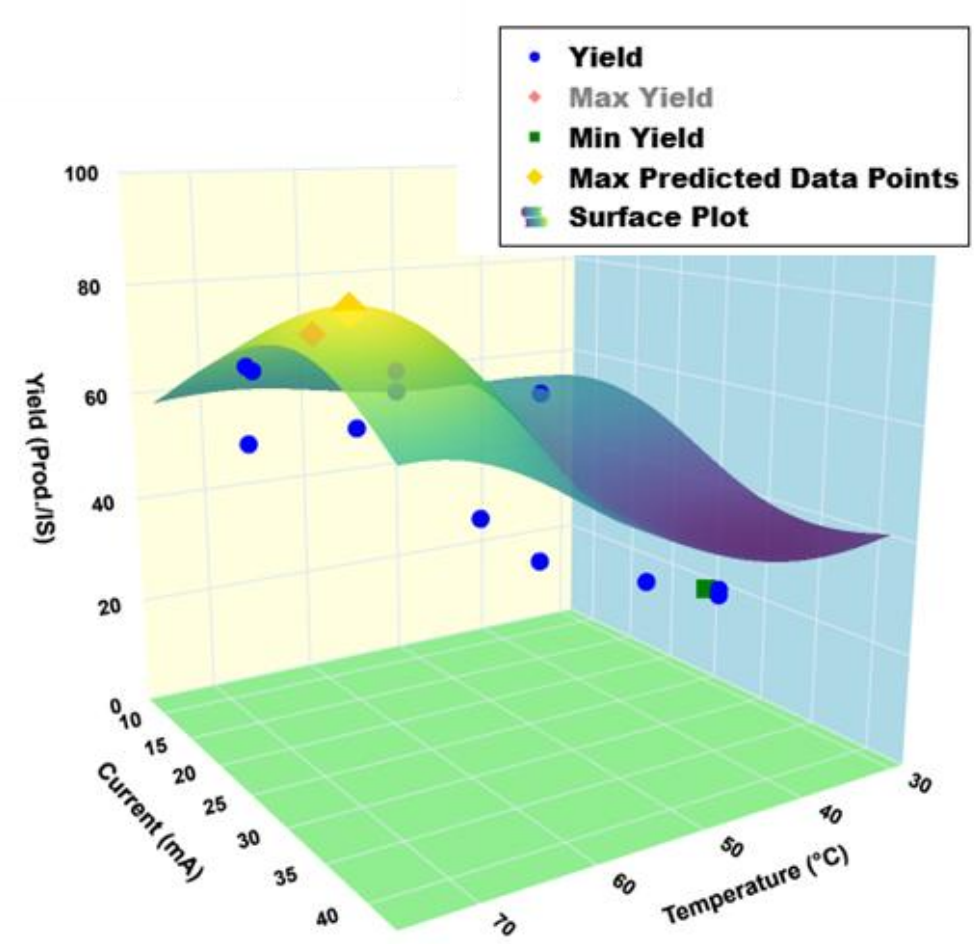


**Figure S14** 3D surface plot of yield vs temperature and current

**Note:** The 3D plot indicates that the optimal conditions—30.3 mA and 71.9 ºC—afford the target product in 79% yield. The plot also demonstrates that the reaction is sensitive to both temperature and current, with lower current and higher temperature favoring the reaction yield, consistent with the results reported by Weix[1].

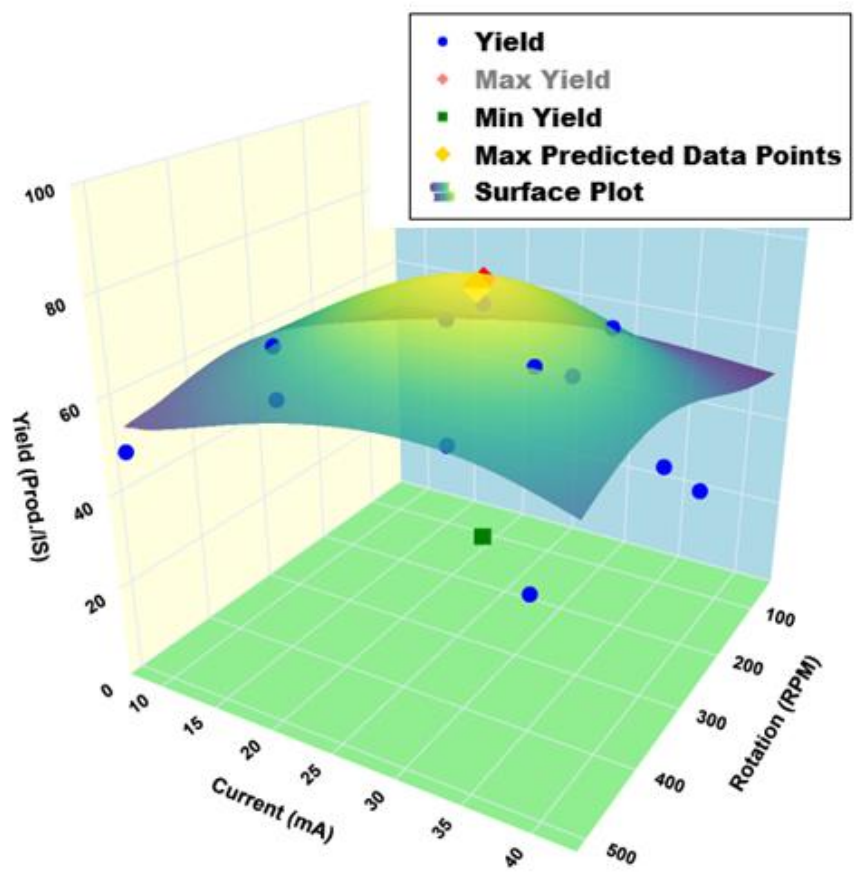


**Figure S15** 3D surface plot of yield vs current and rotation rate

**Note:** The 3D plot indicates that the rotation rate has only a minor effect on the reaction yield, with approximately 250 rpm giving the best results.

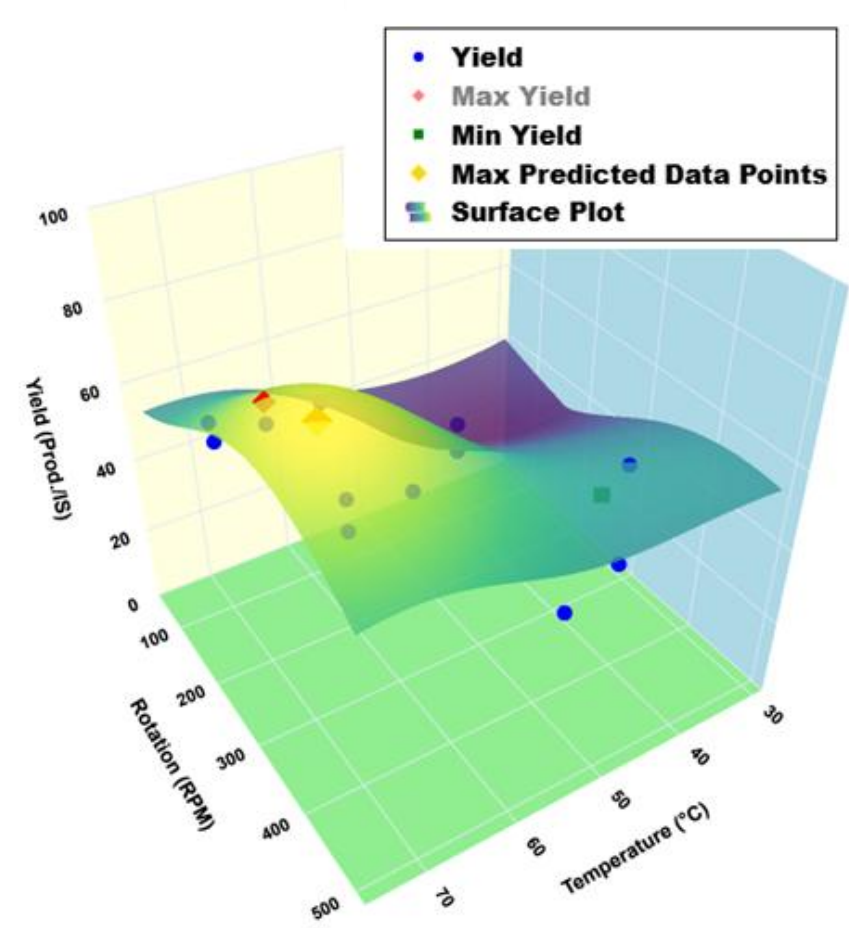


**Figure S16** 3D surface plot of yield vs temperature and rotation rate

### 3.1.12 2D contour map representation model set-up: Predicted reaction yield of 3a

To more clearly illustrate the relationship between the two variables under the third optimized condition, a 2D contour map was also constructed. Gaussian process regression was used to generate approximately 20,000 predicted data points based on 14 sets of experimental data. Among the 20,000 predicted data sets, the data corresponding to the other two variables were selected under the optimized condition of the third

variable, and a 2D contour map was constructed. The code is provided in the accompanying file (2D_contour map.ipynb). Points on the contour map represent the variation in yield with respect to two parameters while the third parameter is held at its optimal value, providing insight into yield trends across the examined variable ranges. By integrating the electrochemical set-up with a 2D contour map, the changes in yield with variation of the reaction variables can be clearly observed.

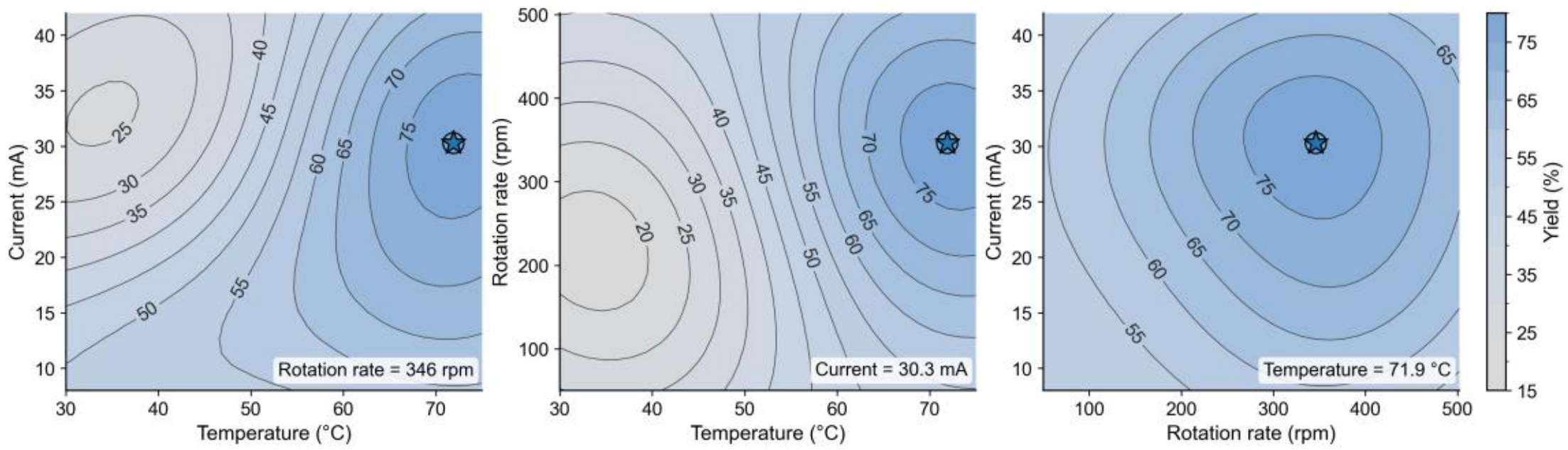


**Figure S17** 2D contour map

### 3.1.13 Accuracy assessment of 3D predicted data via experimental testing

We randomly selected five data points from the 3D predicted surface plot, as shown in the **Scheme 3.6**. These conditions were then tested using a eSTR set-up, and the reaction outcomes were analyzed to obtain the corresponding experimental data (The reaction was stirred at 250 rpm; The preparation of the reaction solution and the operating procedure were the same as described in Section 3.1.1). As shown, the experimental results exhibited only a small deviation (~7%) from the values predicted by the 3D model, demonstrating that the reaction can be effectively analyzed and predicted using 3D modeling.

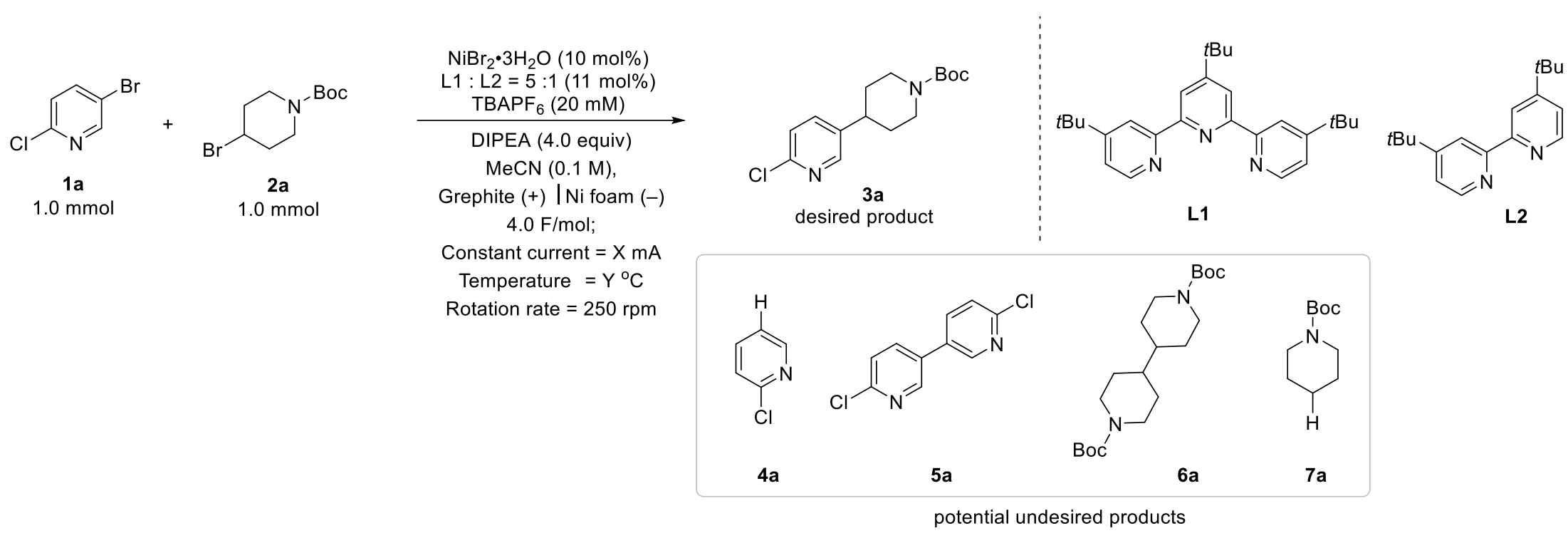

**Table S7** Assessment of 3D surface plot predicted data via experimental testing

| Run order | Temperature (°C) | Current (mA) | **3a** (%) Predicted data | **3a** (%) Experimental data | **1a/2a** recovery | **4a/7a/5a/6a** Side products |
|---|---|---|---|---|---|---|
| 1 | 65 | 32 | 75 | 71 | 5/21 | 3/-/9/- |
| 2 | 58 | 33 | 63 | 60 | 16/33 | 11/2/3/- |
| 3 | 42 | 17 | 57 | 51 | 22/45 | 4/3/-/- |
| 4 | 49 | 34 | 45 | 42 | 35/15 | 11/3/15/6 |
| 5 | 34 | 25 | 46 | 43 | 25/- | 17/2/12/13 |

### 3.1.14 System stability evaluation using electrochemical reactions of varying volumes

To assess the stability and reproducibility of the automated electrochemical platform, several parallel reactions were performed under the predicted optimal conditions. Specifically, three reactions were conducted using 10 mL of reaction solution, and one reaction each was performed with 20 mL, 40 mL, and 60 mL of solution. A total of 200 mL of reaction solution was prepared following the same protocol described above, and the reactions were carried out sequentially on the automated platform. Product collection followed the same sampling procedure as previously described. However, instead of a single outlet tube, a branched tubing system was used to collect the final reaction mixtures. Excess solution was uniformly withdrawn through the branching lines to prevent reaction products from overflowing from the test tubes.

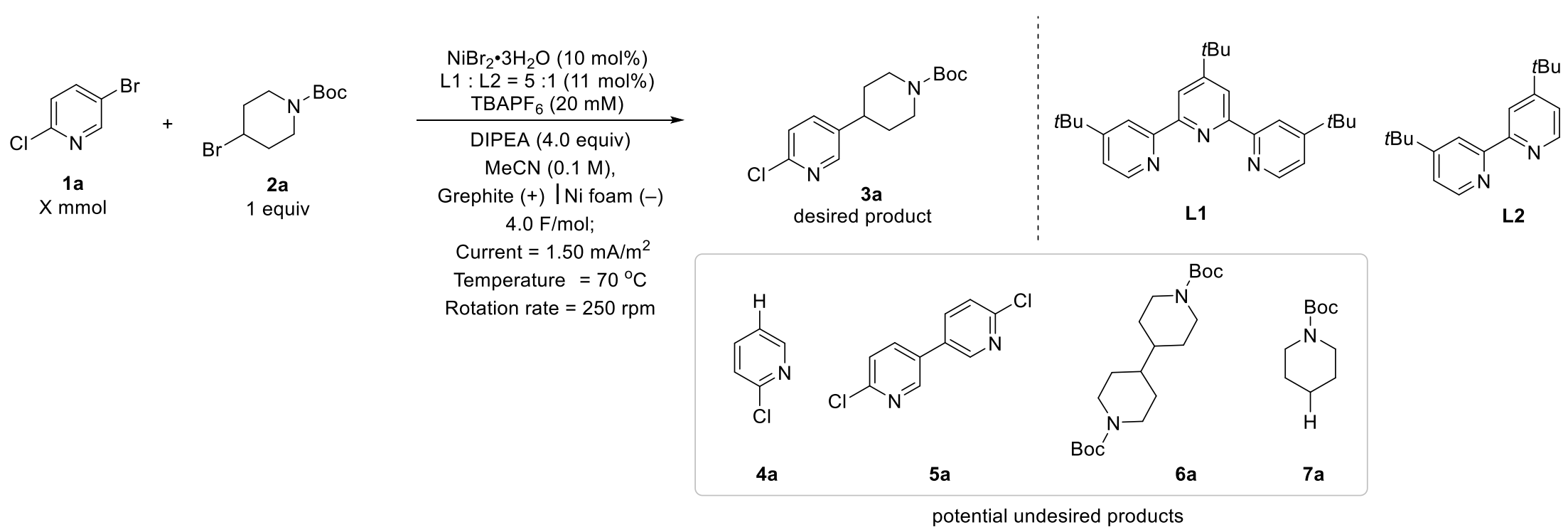


**Table S8** Electrochemical reactions of varying volumes

| Expt. | Reaction Volume (mL) | **3a** (%) | **1a/2a** recovery | **4a/5a/6a/7a** Side products |
|---|---|---|---|---|
| 1 | 10 | 71 | 16/8 | 13/-/6/4 |
| 2 | 10 | 75 | 16/7 | 13/-/4/6 |
| 3 | 10 | 72 | 18/4 | 13/-/2/4 |
| 4 | 20 | 82 | -/- | 11/-/3/- |
| 5 | 40 | 78 | -/- | 11/-/5/- |
| 6 | 60 | 76 | -/- | 13/-/5/- |

### 3.1.15 System stability evaluation in 80 mL-scale electrochemical reactions

To evaluate the stability and scale-up capability of the automated electrochemical platform, four consecutive 80 mL electrochemical reactions were conducted using the device. A total of 400 mL of reaction solution was prepared following the previously described protocol, and each reaction was carried out sequentially on the platform. The four batches afforded yields of 78%, 75%, 72%, and 72%, respectively. Although a slight decrease in yield was observed over time—likely due to the high-intensity electrolysis process—the overall decline was modest (only ~6%). These results demonstrate that the device exhibits good operational stability and is capable of maintaining consistent performance under scaled-up reaction conditions. After completing the four reaction batches, all reaction solutions were combined, filtered, and concentrated to remove the solvent. The crude product was then purified by column chromatography, affording 6.84 g (totally 71% isolated yield) of the desired product.

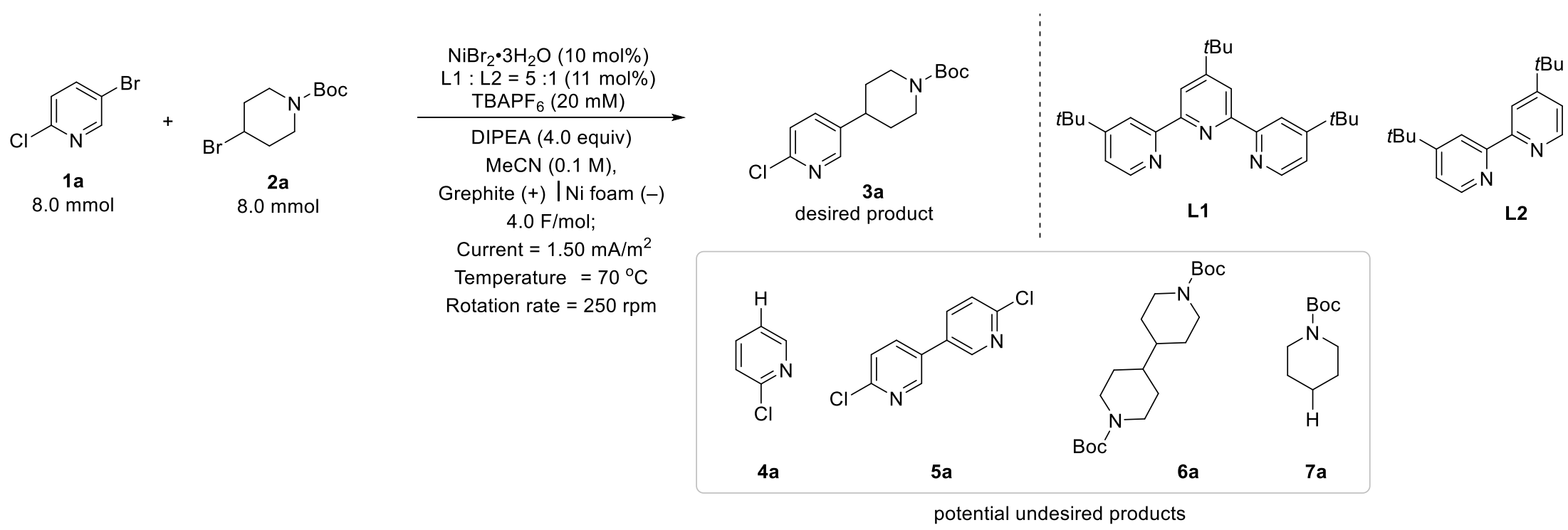


**Table S9** System stability evaluation

| Expt. | Reaction Volume (mL) | **3a** (%) | **1a/2a** recovery | **4a/5a/6a/7a** Side products |
|---|---|---|---|---|
| 1 | 80 | 78 | 10/7 | 13/-/5/2 |
| 2 | 80 | 75 | 9/8 | 10/-/4/2 |
| 3 | 80 | 72 | 12/11 | 12/-/3/6 |
| 4 | 80 | 72 | 10/14 | 13/-/5/3 |

### 3.1.16 System stability test using electrochemical reactions of large scale (40 mmol)

After demonstrating that the platform could successfully perform four consecutive 80 mL-scale (8 mmol) reactions, we next evaluated its capability for larger-scale synthesis to further demonstrate its practical applicability. A 400 mL reaction solution (40 mmol substrate, 0.1 M) was prepared in a round-bottom flask according to the procedure described in section 3.1.2 with 1,3,5-trimethoxybenzene (3.34 g, 20 mmol, 0.5 equiv relative to the substrate **1a**) was added as internal standard. Two peristaltic pumps were employed to enable continuous circulation: one delivered the reaction mixture into the eSTR, while the other removed it, maintaining the reactor volume at approximately 76 mL (The reactor set-up is shown below). In this set-up, the reaction solution continuously cycled between the round-bottom flask and the eSTR. Electrolysis

was carried out at a constant current of 150 mA (1.45 mA/cm$^2$) for 28 hours, with the reaction solution in the flask maintained at 68 ºC. After completion, the reaction mixture was quenched with $H_2O$. The reaction mixture was filtered through a pad of silica gel which was then washed with a 5:1 mixture of pentane/EtOAc. The combined filtrates were transferred to an NMR tube, and the crude yield was determined by $^1$H NMR spectroscopy using 1,3,5-trimethoxybenzene as the internal standard. Under these conditions, the desired product **3a** was obtained in 75% NMR yield. The combined filtrates were concentrated under reduced pressure , and the product was isolated by silica gel flash chromatography (hexane:ethyl acetate = 85:15). The purified product was obtained in 71% isolated yield (8.40 g): $^1$H NMR: (400.0 MHz, $CDCl_3$) $\delta$ 8.25 (d, *J* = 2 Hz, 1H), 7.48 (dd, *J* = 2.5; 8.2 Hz, 1H), 7.30-7.25 (m, 1H), 4.35-4.15 (m, 2H), 2.88-2.74 (m, 2H), 2.72-2.64 (m, 1H), 1.88-1.77 (m, 2H), 1.65-1.53 (m, 2H), 1.48 (s, 9H); $^{13}$C NMR: (100.0 MHz, $CDCl_3$) $\delta$ 154.7, 149.5, 148.5, 139.8, 137.0, 124.1, 79.7, 44.4, 39.6, 32.8, 28.4; The spectral NMR are consistent with those reported in the literature.[1]

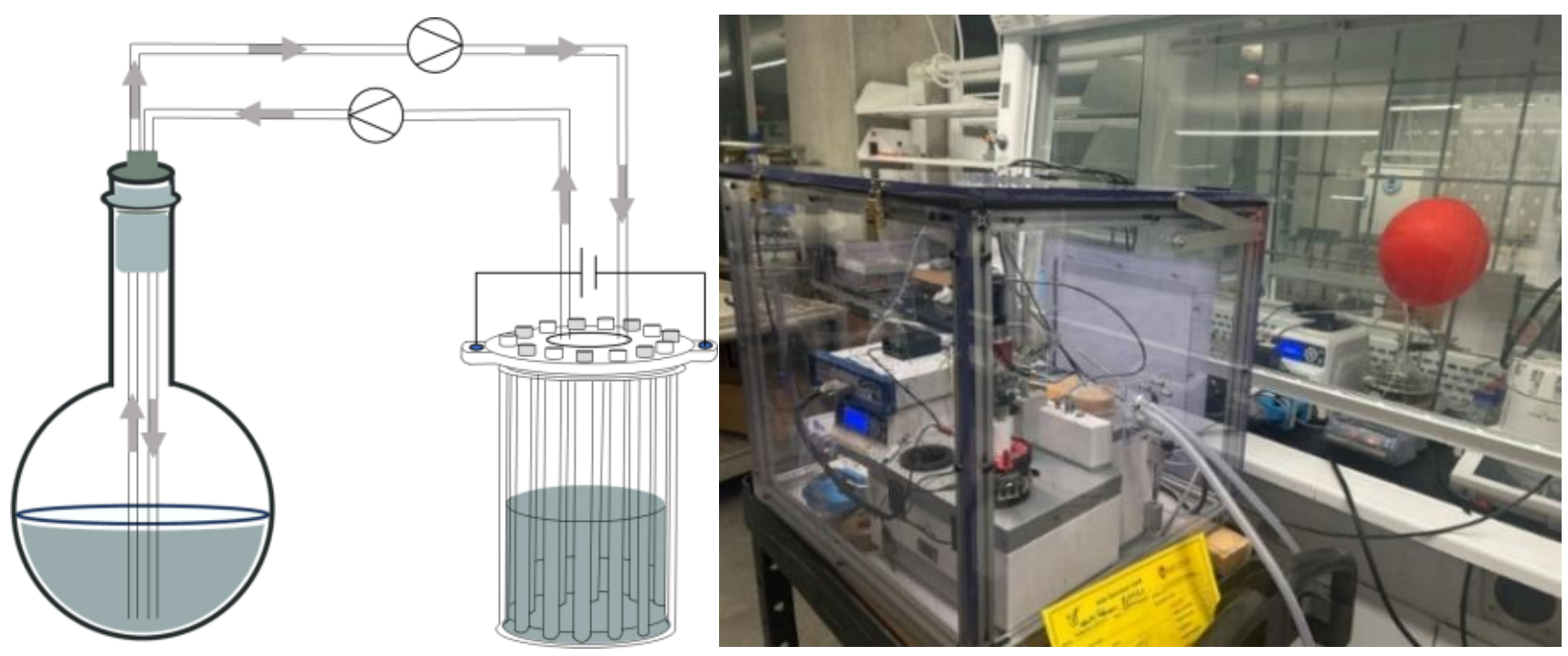

**Figure S18** Schematic illustration based on theory (left); Schematic of the actual set-up (right).

**3.1.17 System stability test using electrochemical reactions of large scale (100 mmol)**

A 1000 mL reaction solution (100 mmol substrate, 0.1 M) was prepared in a round-bottom flask according to the procedure described in section 3.1.2 with 1,3,5-trimethoxybenzene (8.40 g, 50 mmol, 0.5 equiv relative to the substrate **1a**) was added as internal standard. Two peristaltic pumps were employed to enable continuous circulation: one delivered the reaction mixture into the eSTR, while the other removed it, maintaining the reactor volume at approximately 110 mL. In this set-up, the reaction solution continuously cycled between the round-bottom flask and the eSTR. Electrolysis was carried out at a constant current of 250 mA (1.48 mA/cm$^2$) for 43 hours, with the reaction solution in the flask maintained at 68 ºC. After completion, the reaction mixture was worked up, as described in 3.1.16. The combined filtrates were transferred to an NMR tube, and the crude yield was determined by $^1$H NMR spectroscopy using 1,3,5-trimethoxybenzene (0.5 equiv relative to the substrate **1a**) as the internal standard. Under these conditions, the desired product **3a** was obtained in 71% NMR yield (see $^1$H NMR spectrum of the crude reaction product in Section 5). The combined filtrates were concentrated under reduced pressure, and the product

was isolated by silica gel flash chromatography (hexane:ethyl acetate = 85:15). The purified product was obtained in 66% isolated yield (19.52 g).

### 3.2 General procedure for optimizing the nickel-catalyzed electrochemical C($sp^2$)–C($sp^3$) reductive relay cross-coupling of alkyl halides to aryl halides

#### 3.2.1 Reproduction of the reported procedure

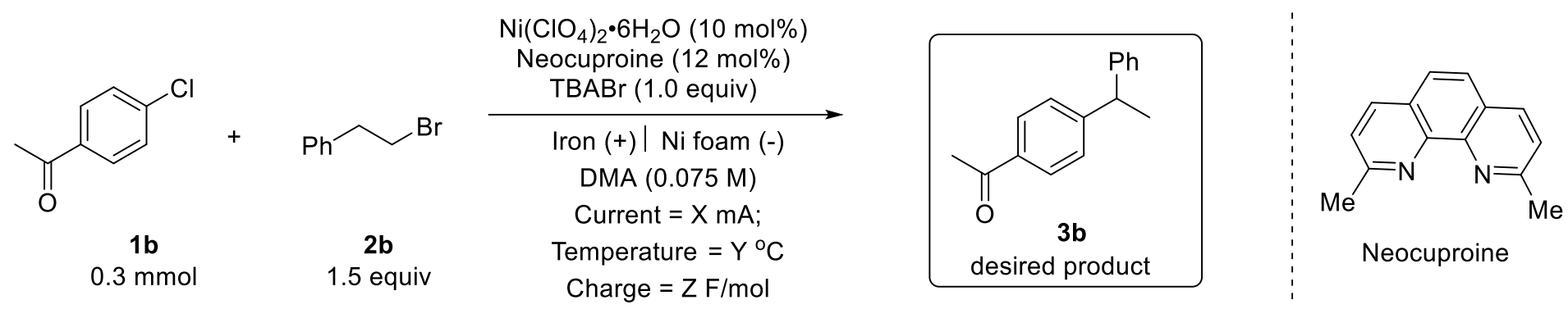


The reaction was reproduced following the reported procedure with minor modifications.[2] A test tube (16 x 100 mm) equipped with a 10 mm cross-shaped magnetic stir bar was dried overnight in an oven at 110 ºC. The hot test tube was then transferred to the glovebox antechamber, evacuated, allowed to cool to room temperature, and subsequently brought into the glovebox. $Ni(ClO_4)_2 \cdot 6H_2O$ (11.0 mg, 10 mol%), Neocuproine (7.5 mg, 12 mol%), *n*$Bu_4NBr$ (96.7 mg, 1.0 equiv), and DMA (4 mL) were added to an oven-dried 10 mL test tube and stirred for 30 min to afford a homogeneous solution. 4'-Chloroacetophenone **1b** (46.2 mg, 0.30 mmol, 1.0 equiv) and (2-Bromoethyl)benzene **2b** (62 µL, 0.45 mmol, 1.5 equiv) were then added sequentially. The reaction vessel was sealed with the electrode cap assembly. The reaction mixture was electrolyzed at 70 ºC under vigorous stirring (1500 rpm) at a constant current of −6 mA for 10 h using Ni foam as the cathode and Iron plate as the anode. Upon completion, the reaction vessel was removed from the glovebox and quenched with saturated aqueous $NaHCO_3$ (200 µL). A solution of 1,3,5-trimethoxybenzene in $CHCl_3$ (100 µL, 1.0 M) was added as an internal standard, and the mixture was stirred for an additional 5 min. An aliquot (100 µL) of the reaction mixture was withdrawn and passed through a short pad of silica gel, which was subsequently washed with $CDCl_3$ (3 × 200 µL). The combined filtrates were transferred to an NMR tube, and the crude yield was determined by $^1H$ NMR spectroscopy using 1,3,5-trimethoxybenzene as the internal standard. Under these conditions, the desired product **3b** was obtained in 69% NMR yield.

#### 3.2.2 Preparation of the reaction solution for condition screen

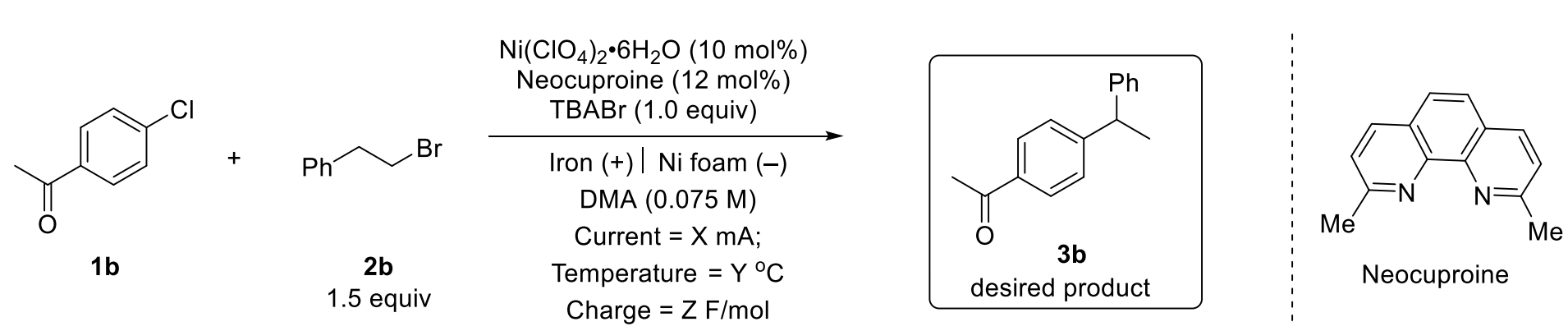


A 500 mL round-bottom flask equipped with a cross-shaped magnetic stir bar was dried in an oven at 110 ºC overnight. The hot round-bottom flask was placed in the glove box antechamber, evacuated, and allowed

to cool before being transferred into the glove box. $Ni(ClO_4)_2·6H_2O$ (2.0 mmol, 731.4 mg), neocuproine (2.40 mmol, 499.2 mg), and DMA (0.75 M; 250 mL) were sequentially added. The resulting suspension was stirred at r.t. for 30 min. Subsequently, TBABr (20.0 mmol, 6.44 g), (2-bromoethyl)benzene (30.0 mmol, 4.10 mL), 1-(4-chlorophenyl)ethan-1-one (20.0 mmol, 3.09 g), and 1,3,5-trimethoxybenzene (internal standard, 6.67 mmol; 1.12 g) were added in that order. The flask was then sealed with a rubber stopper and put into the purge box, and a simple magnetic stirring device was used to maintain uniform stirring of the solution and ensure homogeneous mixing throughout the reaction.

**Note:** Compared with Mei's small scale reaction[2], this reaction employs a much larger volume of solution, making the pre-stirring of the ligand and precatalyst particularly important.

### 3.2.3 Set-up of reaction conditions

Three key parameters—current, temperature, and rotation rate—were selected to investigate their influence on the reaction. The ranges of these variables were determined based on the conditions reported in Mei's work. Because the reaction is highly sensitive to current density, the applied current was varied from 10 to 25 mA, corresponding to a current density of 0.60–1.49 $mA/cm^2$. For reference, in a 10 mL reaction volume, the immersed surface area of the Ni foam electrode was 1.2 cm × 14 cm, giving a total area of 16.8 $cm^2$; therefore, an applied current of X mA corresponds to a current density of X/16.8 $mA/cm^2$. The reaction temperature was varied between 5 ºC and 20 ºC due to the sensitivity of the relay process, and the effect of total charge was also evaluated in the range of 3–6 F/mol. A Design of Experiments (DoE) strategy was implemented to generate 14 reaction conditions, as summarized below. Minor adjustments were made to the conditions during the actual experiments to meet practical operational requirements.

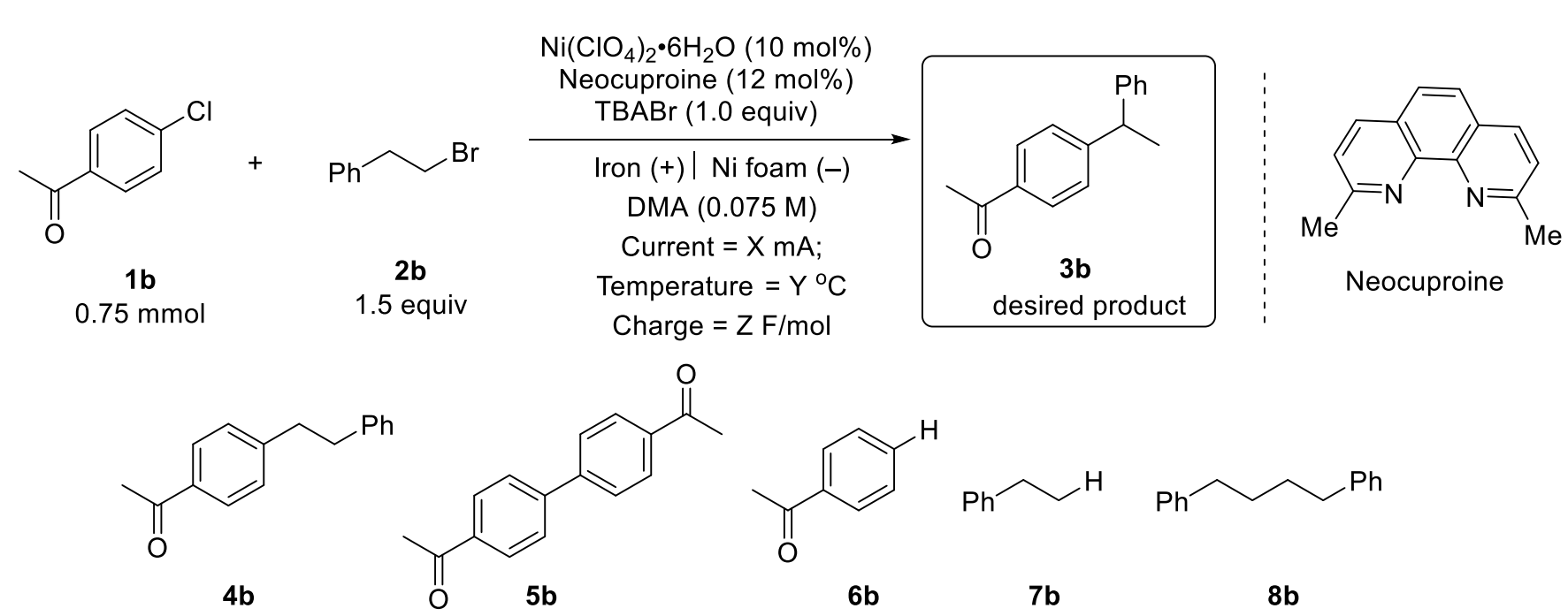

**Table S10** Variable ranges of the factors

| Factor | Lower | Higher |
|---|---|---|
| Temperature | 5 °C | 20 °C |
| Current | 10 mA | 25 mA |
| Charge | 3 F/mol | 6 F/mol |

**Table S11** Theoretical design of condition screening via DoE

| Run order | Temperature (°C) | Current (mA) | Charge (F/mol) |
|---|---|---|---|
| 1 | 5 | 10 | 3 |
| 2 | 20 | 10 | 3 |
| 3 | 5 | 25 | 3 |
| 4 | 20 | 25 | 3 |
| 5 | 5 | 10 | 6 |
| 6 | 20 | 10 | 6 |
| 7 | 5 | 25 | 6 |
| 8 | 20 | 25 | 6 |
| 9 | 25.11 | 17.5 | 4.5 |
| 10 | -0.11 | 17.5 | 4.5 |
| 11 | 12.5 | 30.11 | 4.5 |
| 12 | 12.5 | 4.89 | 4.5 |
| 13 | 12.5 | 17.5 | 7.02 |
| 14 | 12.5 | 17.5 | 1.98 |

**Table S12** Modified condition screening table

| Run order | Temperature (°C) | Current (mA) | Charge (F/mol) |
|---|---|---|---|
| 1 | 5 | 10 | 3 |
| 2 | 20 | 10 | 3 |
| 3 | 5 | 25 | 3 |
| 4 | 20 | 25 | 3 |
| 5 | 5 | 10 | 6 |
| 6 | 20 | 10 | 6 |
| 7 | 5 | 25 | 6 |
| 8 | 20 | 25 | 6 |
| 9 | 25 | 18 | 4.5 |
| 10 | 0 | 18 | 4.5 |
| 11 | 13 | 30 | 4.5 |
| 12 | 13 | 5 | 4.5 |
| 13 | 13 | 18 | 7.02 |
| 14 | 13 | 18 | 1.98 |

**Note:** The condition table was generated using the Design of Experiments tool on the numiqo website (https://numiqo.com/statistics-calculator/design-of-experiments).

### 3.2.4 Results analysis

After the reaction completed, a 75 µL aliquot of the product solution was taken and passed through a short silica gel column in a pipette, eluting with $CDCl_3$ (3 × 300 µL). The resulting filtrate was then subjected to NMR analysis, and the results are shown below.

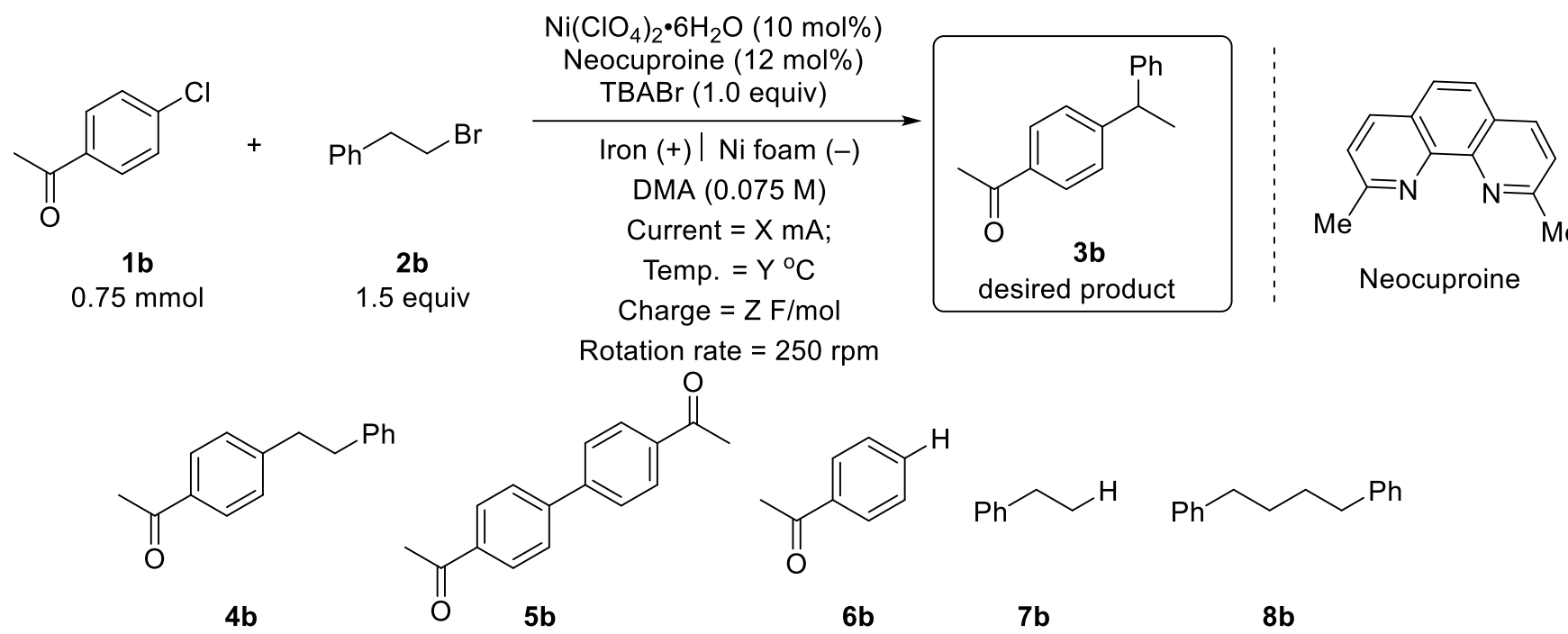


**Table S13** The crude $^1$H NMR spectroscopic data of reaction

| Expt. | Temperature (°C) | Current (mA) | Charge (F/mol) | **3a** (%) | **1a/2a** Recovery (%) | Yield of **5a/6a/8a** (%) |
|---|---|---|---|---|---|---|
| 1 | 5 | 10 | 3 | 2 | 88/85 | -/6/5 |
| 2 | 20 | 10 | 3 | 40 | 33/16 | 2/7/10 |
| 3 | 5 | 25 | 3 | 2 | 84/67 | 1/5/13 |
| 4 | 20 | 25 | 3 | 66 | 10/2 | 2/10/13 |
| 5 | 5 | 10 | 6 | 4 | 78/69 | 1/10/9 |
| 6 | 20 | 10 | 6 | 40 | 31/13 | 3/8/12 |
| 7 | 5 | 25 | 6 | 2 | 76/61 | 3/10/13 |
| 8 | 20 | 25 | 6 | 44 | 12/1 | 4/11/15 |
| 9 | 25 | 18 | 4.5 | 48 | 11/4 | 3/10/14 |
| 10 | 0 | 18 | 4.5 | 1 | 78/49 | 6/7/16 |
| 11 | 13 | 30 | 4.5 | 38 | 22/15 | 10/9/17 |
| 12 | 13 | 5 | 4.5 | 34 | 35/29 | 4/11/11 |
| 13 | 13 | 18 | 7.02 | 2 | 47/29 | 11/17/27 |
| 14 | 13 | 18 | 1.98 | 2 | 69/52 | 4/10/15 |

### 3.2.5 3D representation model set-up: Predicted reaction yield of 3b

Reaction yields across the reaction space were predicted via Gaussian process regression and visualized in a 3D plot. The detailed code for generating the 3D plot is provided in an accompanying file (3D plot code.ipynb). A 3D model was constructed based on data obtained from the DoE screening to analyze and predict trends in reaction yield. The model examines the potential relationships between pairs of key variables, including current and temperature, current and charge, and temperature and charge.

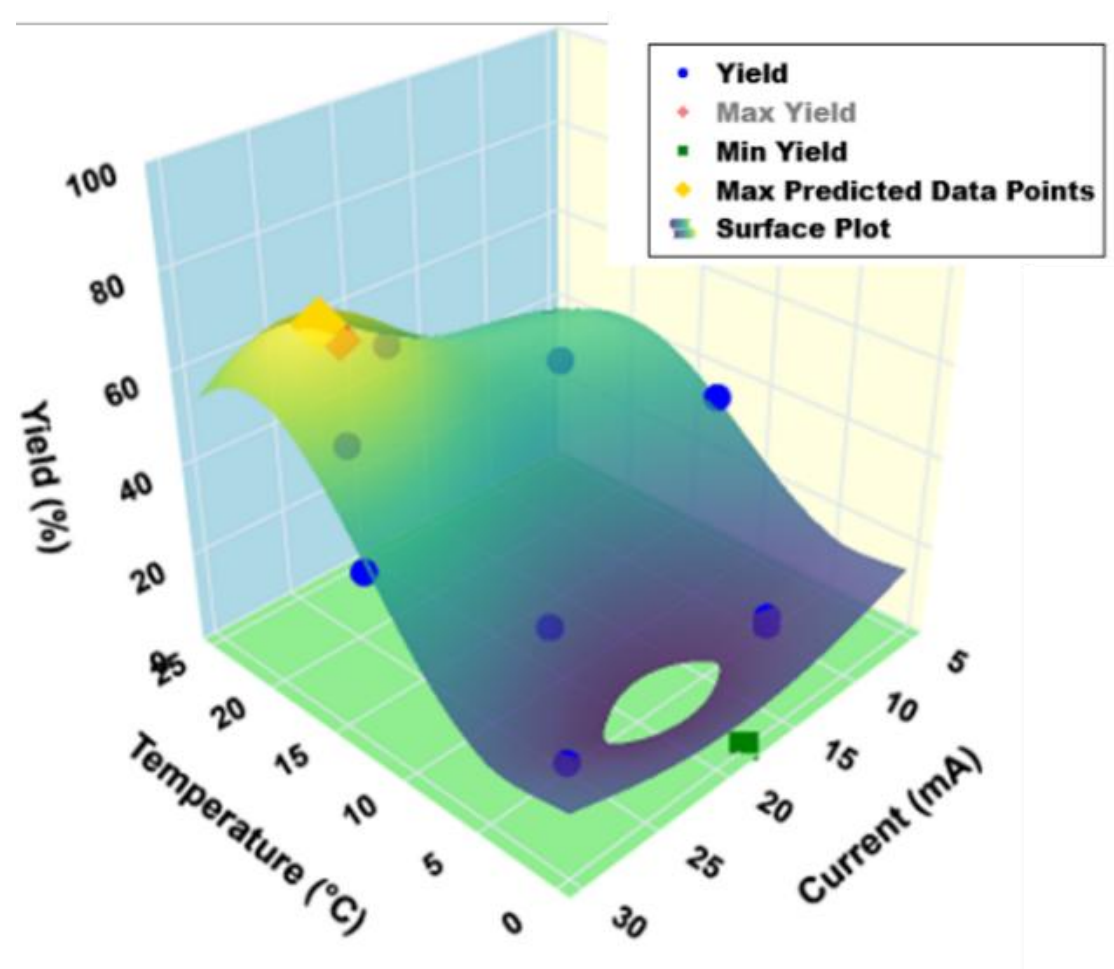


**Figure S19** 3D surface plot of yield vs temperature and current

**Note:** As shown in the 3D plot, the reaction is particularly sensitive to temperature. According to the mechanism proposed in Mei's paper,[2] the reductive relay process is highly sensitive to temperature. Elevated temperatures promote both the oxidation pathway and the dissociation of the alkene from the Ni catalyst center. However, this dissociation is detrimental to the desired catalytic cycle. Therefore, an optimal balance must be maintained between the oxidation process and the dissociation step. As a result, temperatures that are too high or too low both lead to decreased reaction yields.

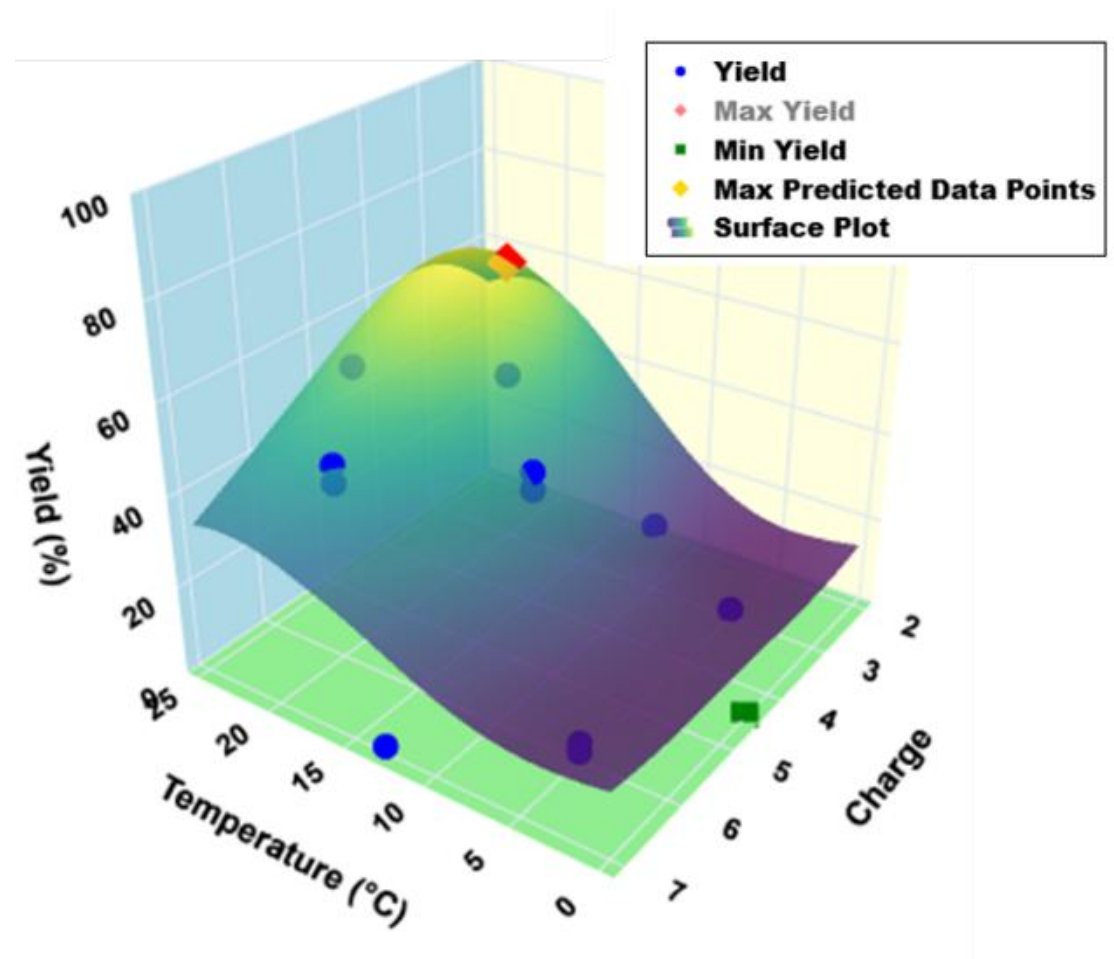


**Figure S20** 3D surface plot of yield vs temperature and charge

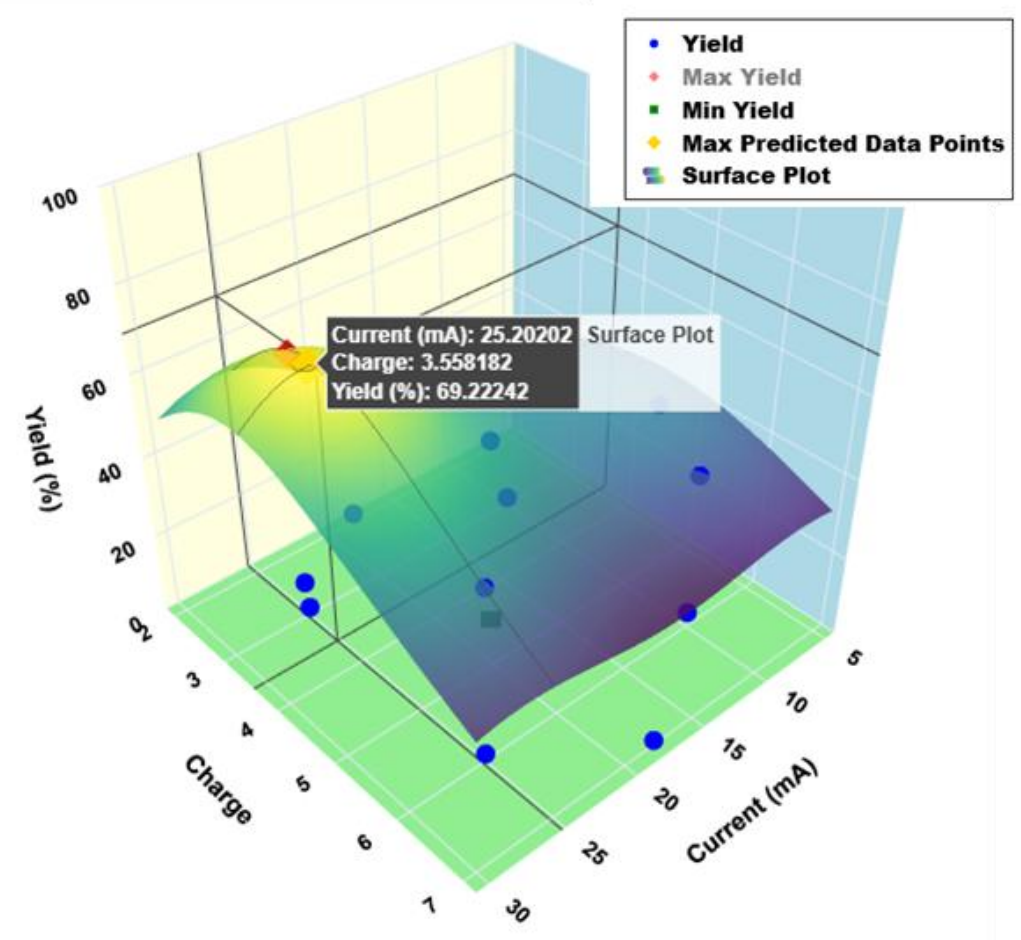


**Figure S21** 3D surface plot of yield vs current and charge

**Note:** As shown in the 3D plot, the optimized charge is 3.58 F/mol, which is very close to the best practical value of 3.7 F/mol reported in Mei's paper.[3] This strong agreement indicates that our 3D plot can reliably predict optimized parameters and serve as a useful guide for reaction condition screening.

### 3.2.6 2D contour map representation model set-up: Predicted reaction yield of 3b

To more clearly illustrate the relationship between the two variables under the third optimized condition, a 2D contour map was also constructed. Gaussian process regression was used to generate approximately 20,000 predicted data points based on 14 sets of experimental data. Among the 20,000 predicted data sets, the data corresponding to the other two variables were selected under the optimized condition of the third variable, and a 2D contour map was constructed. The code is provided in the accompanying file (2D_contour map.ipynb). Points on the contour map represent the variation in yield with respect to two parameters while the third parameter is held at its optimal value, providing insight into yield trends across the examined variable ranges. By integrating the electrochemical set-up with a 2D contour map, the changes in yield with variation of the reaction variables can be clearly observed.

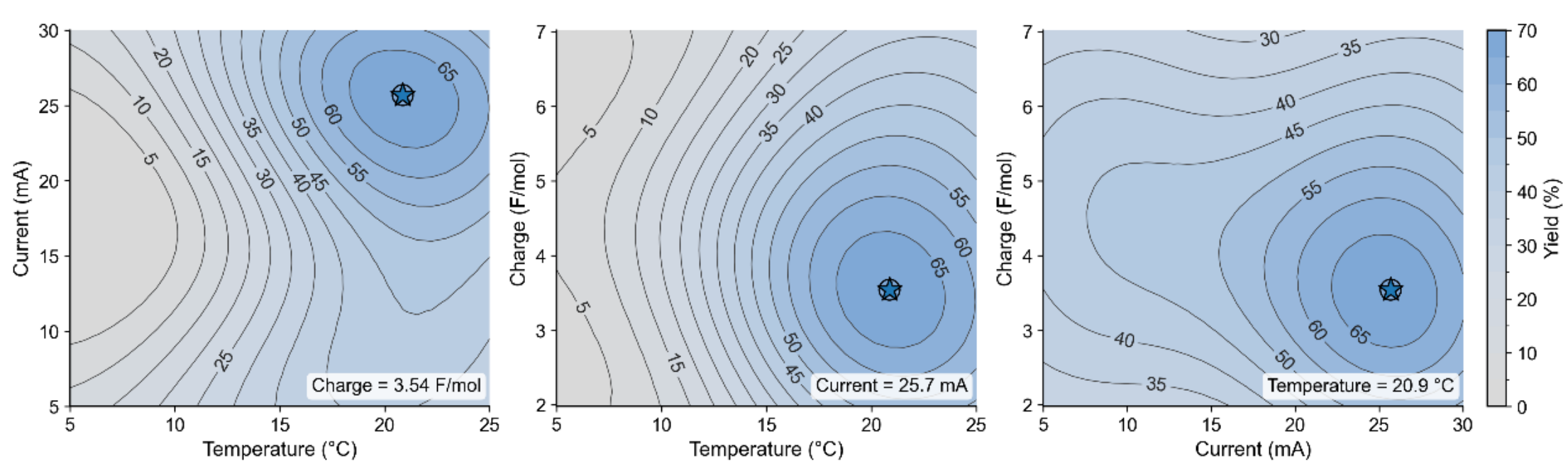


**Figure S22** 2D contour map

### 3.2.7 System stability test using electrochemical reactions of large scale

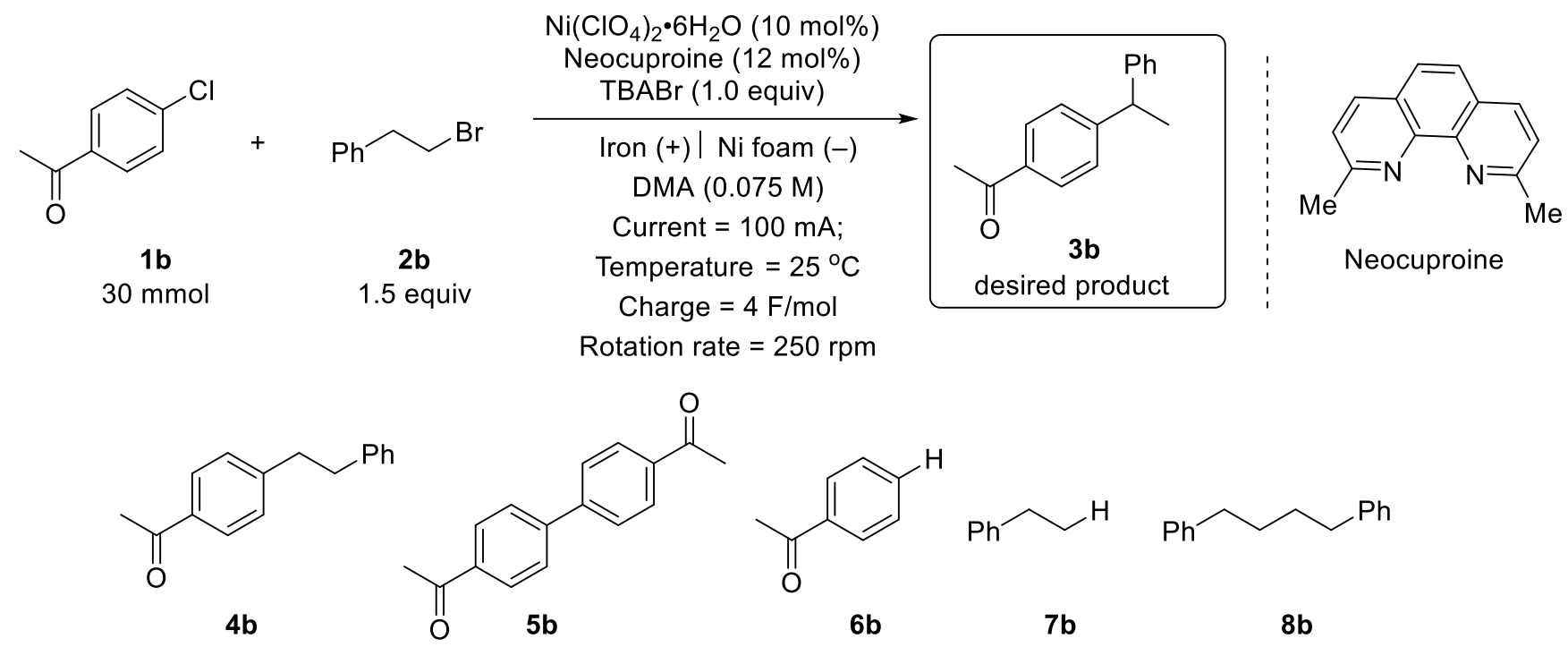


After demonstrating that the platform could successfully perform condition screening, we next evaluated its capability for larger-scale synthesis to further showcase its practical applicability. A 400 mL reaction solution (30 mmol substrate, 0.075 M) was prepared in a round-bottom flask according to the procedure described in section 3.2.2 with 1,3,5-trimethoxybenzene (2.52 g, 15 mmol, 0.5 equiv relative to the substrate **1b**) added as internal standard. Two peristaltic pumps were employed to enable continuous circulation: one delivered the reaction mixture into the eSTR, while the other removed it, maintaining the reactor volume at approximately 40 mL. In this set-up, the reaction solution continuously cycled between the round-bottom flask and the eSTR. After completion, the reaction mixture was quenched with saturated aqueous $NaHCO_3$. The reaction mixture was filtered through a pad of silica gel which was then washed with a 5:1 mixture of pentane/EtOAc. The combined filtrates were transferred to an NMR tube, and the crude yield was determined by $^1$H NMR spectroscopy using 1,3,5-trimethoxybenzene as the internal standard. The desired product **3b** was obtained in 70% NMR yield (see $^1$H NMR spectrum of the crude reaction product in Section 5). The combined filtrates were concentrated under reduced pressure, and the product was isolated by silica gel flash chromatography (hexane:ethyl acetate = 90:10). The purified product was obtained in 64% isolated yield (4.30 g): $^1$H NMR: (400.0 MHz, $CDCl_3$) $\delta$ 7.89 (d, $J$ = 8.1 Hz, 2H), 7.30 (dd, $J$ = 10.5, 7.8 Hz, 4H), 7.25 – 7.16 (m, 3H), 4.21 (q, $J$ = 7.2 Hz, 1H), 2.57 (s, 3H), 1.66 (d, $J$ = 7.2 Hz, 3H).$^{13}$C NMR: (100.0 MHz, $CDCl_3$) $\delta$ 197.8, 152.0, 145.3, 135.2, 128.6, 128.5, 127.8, 127.6, 126.4, 44.8, 26.6, 21.5. The spectral NMR are consistent with those reported in the literature.[2]

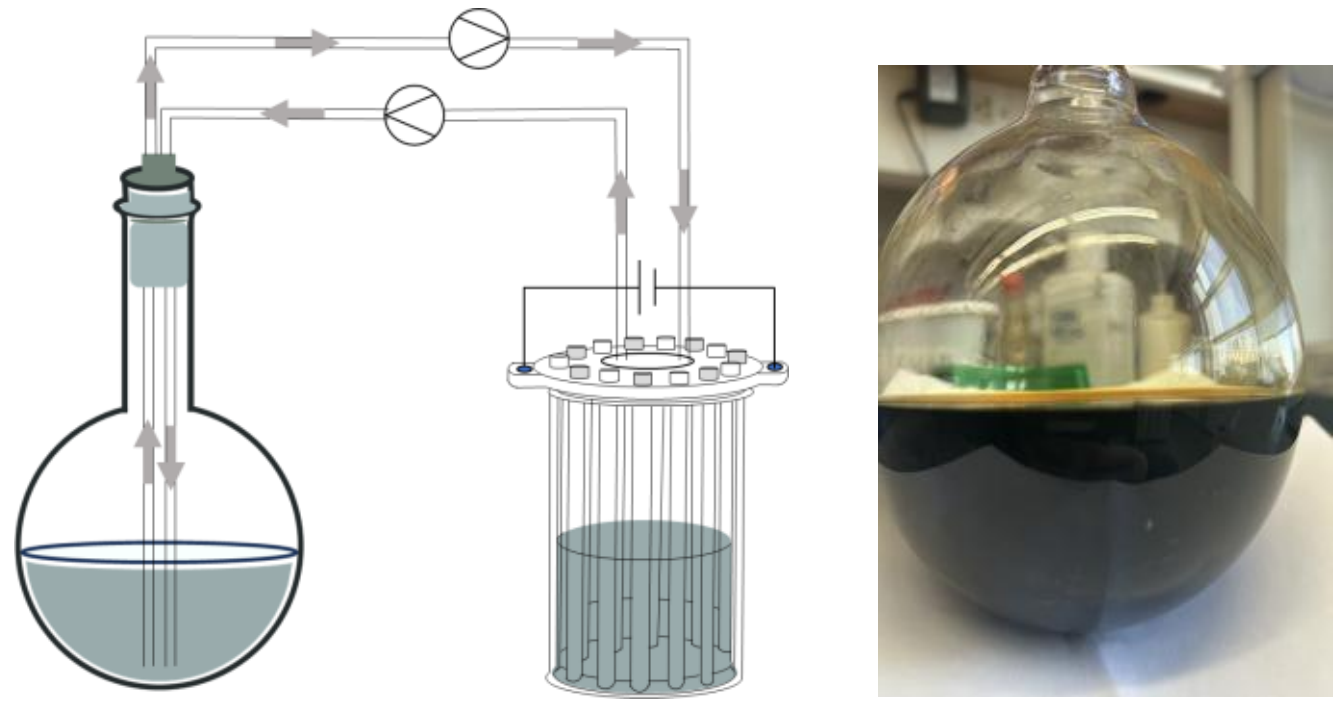

**Figure S23** Schematic illustration based on theory (left); The reaction solution (right).

### 3.3 General procedure for optimizing the nickel-catalyzed electrochemical dehydroxylative cross-coupling of alcohols with aryl halides

### 3.3.1 Reproduction of the reported procedure

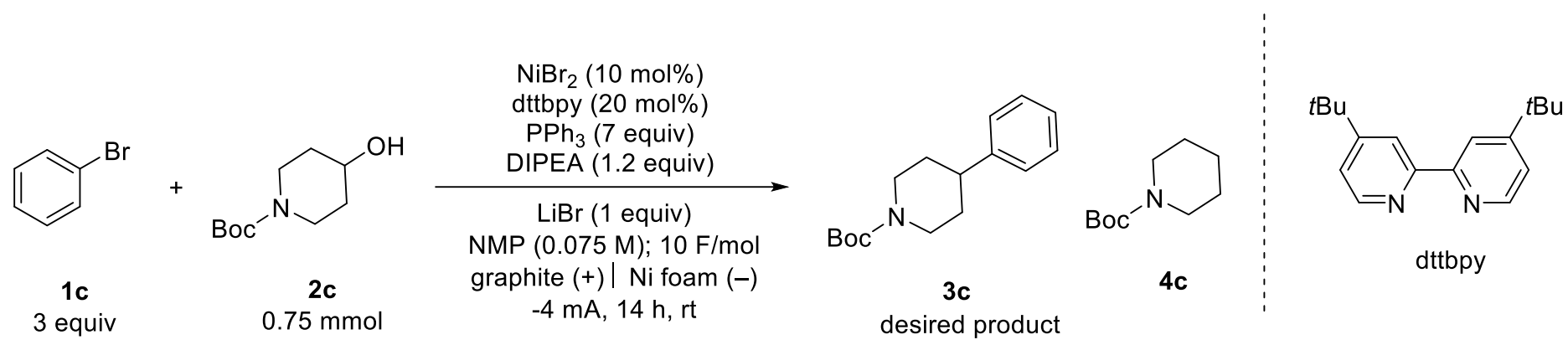


The reaction was reproduced following the reported procedure with minor modifications.[3] A test tube (16 x 100 mm) equipped with a 10 mm cross-shaped magnetic stir bar was dried overnight in an oven at 110 ºC. The hot test tube was then transferred to the glovebox antechamber, evacuated, allowed to cool to room temperature, and subsequently brought into the glovebox. $NiBr_2$ (4.4 mg, 0.02 mmol), dttbpy (10.7 mg, 0.04 mmol), and anhydrous NMP (3 mL) were added. The resulting mixture was stirred at 100 ℃ until a clear green solution was afforded. LiBr (17.4 mg, 0.2 mmol, 1.0 equiv), $PPh_3$ (370.0 mg, 7.0 equiv), DIPEA (40 μL, 0.24 mmol, 1.2 equiv), Bromobenzene **1c** (64 μL, 0.6 mmol, 3.0 equiv), and alcohol **2c** (40.0 mg, 0.2 mmol, 1.0 equiv) were added. The resulting mixture was stirred until all reactants were completely dissolved (ca. 5 minutes). The reaction vessel was sealed with the electrode cap assembly. The reaction mixture was electrolyzed at rt under vigorous stirring (1500 rpm) at a constant current of −4 mA for 14 h using Ni foam as the cathode and graphite as the anode. Upon completion, the reaction vessel was removed from the glovebox and quenched with $H_2O$ (200 μL). A solution of 1,3,5-trimethoxybenzene in $CHCl_3$ (100 μL, 1.0 M) was added as an internal standard, and the mixture was stirred for an additional 5 min. An aliquot (100 μL) of the reaction mixture was withdrawn and passed through a short pad of silica gel, which was subsequently washed with $CDCl_3$ (3 × 200 μL). The combined filtrates were transferred to an NMR tube, and the crude yield was determined by $^1H$ NMR spectroscopy using 1,3,5-trimethoxybenzeneas the internal standard. Under these conditions, the desired product **3c** was obtained in 52% NMR yield

### 3.3.2 Preparation of the reaction solution for condition screen

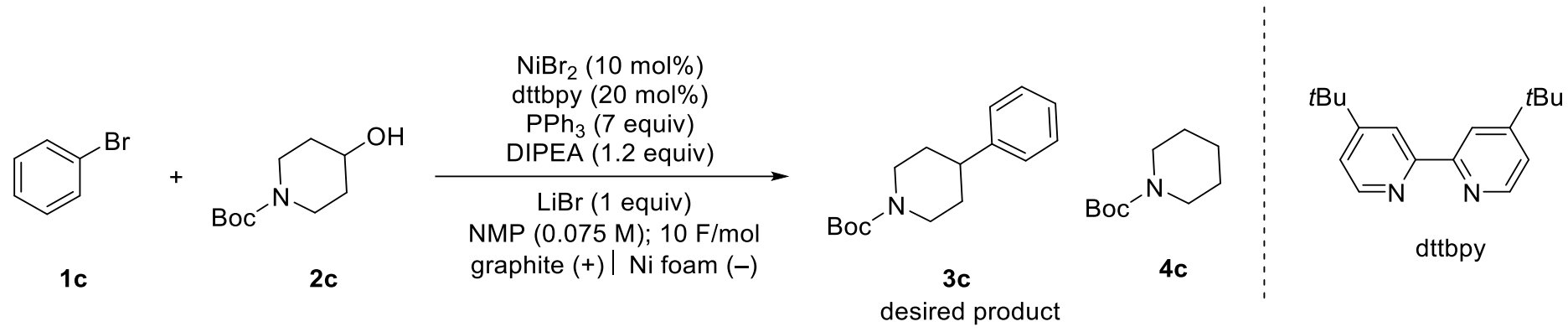


A 500 mL round-bottom flask equipped with a cross-shaped magnetic stir bar was dried in an oven at 110 ºC overnight. The hot round-bottom flask was placed in the glove box antechamber, evacuated, and allowed to cool before being transferred into the glove box. $NiBr_2$ (2.0 mmol, 0.44 g), 4,4'-di-tert-butyl-2,2'-bipyridine (4.0 mmol, 1.07 g), and NMP (0.075 M; 250 mL) were sequentially added. The resulting suspension was stirred at r.t. for 30 min. Subsequently, LiBr (20.0 mmol, 1.55 g), triphenylphosphine (140.0 mmol, 36.81 g), bromobenzene (60.0 mmol, 6.40 mL), *tert*-butyl 4-hydroxypiperidine-1-carboxylate (20.0 mmol, 4.02 g), 1,3,5-trimethoxybenzene (internal standard, 6.67 mmol; 1.12 g), and DIPEA (24.0 mmol, 4.2 mL) were added in that order. The flask was then sealed with a rubber stopper and put into the purge box, and a simple magnetic stirring device was used to maintain uniform stirring of the solution and ensure homogeneous mixing throughout the reaction.

**Note:** Compared with Li's small scale reaction[3], this reaction employs a much larger volume of solution, making the pre-stirring of the ligand and precatalyst particularly important (Insufficient stirring may lead to coordination differences).

### 3.3.3 Set-up of reaction conditions

Three parameters—current, temperature, and rotation rate—were selected to evaluate their effect on the reaction. The ranges of these variables were chosen based on the condition screening reported in Li's paper.[2] Since the reaction is sensitive to current density, the current density was varied from 0.89 to 2.98 $mA/cm^2$ (When the reaction volume was 10 mL, the immersed surface area of the Ni foam electrode was 1.2 $cm^2$ × 14. Therefore, the applied current corresponds to a current density of X mA/16.8 $cm^2$. and currents of 15–50 mA). The temperature range was set from 15 ºC to 45 ºC, and the rotation rate was varied from 100 to 300 rpm. A Design of Experiments (DoE) approach was employed to generate 14 reaction runs, as summarized below. The conditions were slightly adjusted during the actual experiments to accommodate operational requirements.

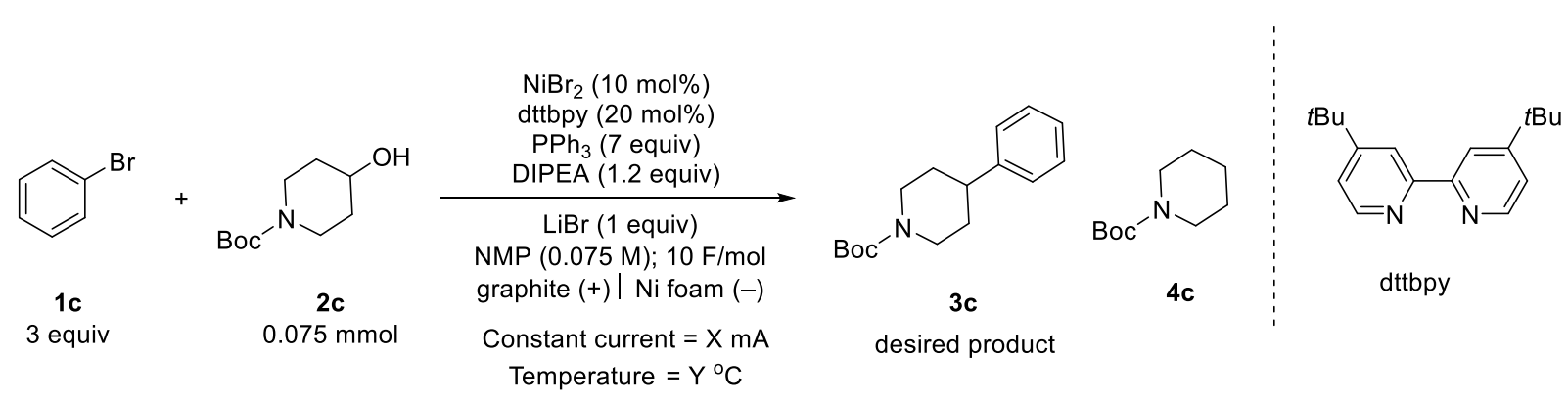

**Table S14** Variable ranges of the factors

| Factor | Lower | Higher |
|---|---|---|
| Temperature | 15 °C | 45 °C |
| Current | 15 mA | 50 mA |
| Rotation rate | 100 rpm | 300 rpm |

**Table S15** Theoretical design of condition screening via DoE

| Run order | Temperature (°C) | Current (mA) | Rotation rate (rpm) |
|---|---|---|---|
| 1 | 15 | 15 | 100 |
| 2 | 45 | 15 | 100 |
| 3 | 15 | 50 | 100 |
| 4 | 45 | 50 | 100 |
| 5 | 15 | 15 | 300 |
| 6 | 45 | 15 | 300 |
| 7 | 15 | 50 | 300 |
| 8 | 45 | 50 | 300 |
| 9 | 55.23 | 32.5 | 200 |
| 10 | 4.77 | 32.5 | 200 |
| 11 | 30 | 61.93 | 200 |
| 12 | 30 | 3.07 | 200 |
| 13 | 30 | 32.5 | 368.18 |
| 14 | 30 | 32.5 | 31.82 |

**Note:** This table was automatically generated by the DoE based on the three selected parameters (https://numiqo.com/statistics-calculator/design-of-experiments). Some values in the table may be unrealistic and require adjustment to align with practical screening conditions. The DoE-generated table provides an organized framework for positioning data points, which facilitates the subsequent application of Gaussian process regression to construct a more accurate 3D response surface plot.

**Table S16** Modified condition screening table

| Run order | Temperature (°C) | Current (mA) | Rotation rate (rpm) |
|---|---|---|---|
| 1 | 15 | 15 | 100 |
| 2 | 45 | 15 | 100 |
| 3 | 15 | 50 | 100 |
| 4 | 45 | 50 | 100 |
| 5 | 15 | 15 | 300 |
| 6 | 45 | 15 | 300 |
| 7 | 15 | 50 | 300 |
| 8 | 45 | 50 | 300 |
| 9 | 55 | 33 | 200 |
| 10 | 10 | 33 | 200 |
| 11 | 30 | 62 | 200 |
| 12 | 30 | 10 | 200 |
| 13 | 30 | 33 | 368 |
| 14 | 30 | 33 | 50 |

**Note***:* This table represents the actual screening conditions of the reaction, closely reflecting the practical experiments. It ensures that the data points are sufficiently distributed to facilitate the construction of a 3D plot.

### 3.3.4 Results analysis

After the reaction completed, 75 µL aliquot of the product solution was taken from the corresponding tube and passed through a short silica gel column in a pipette, eluting with $CDCl_3$ (3 × 300 µL). The resulting filtrate was then subjected to $^1$H NMR analysis, and the results are shown below.

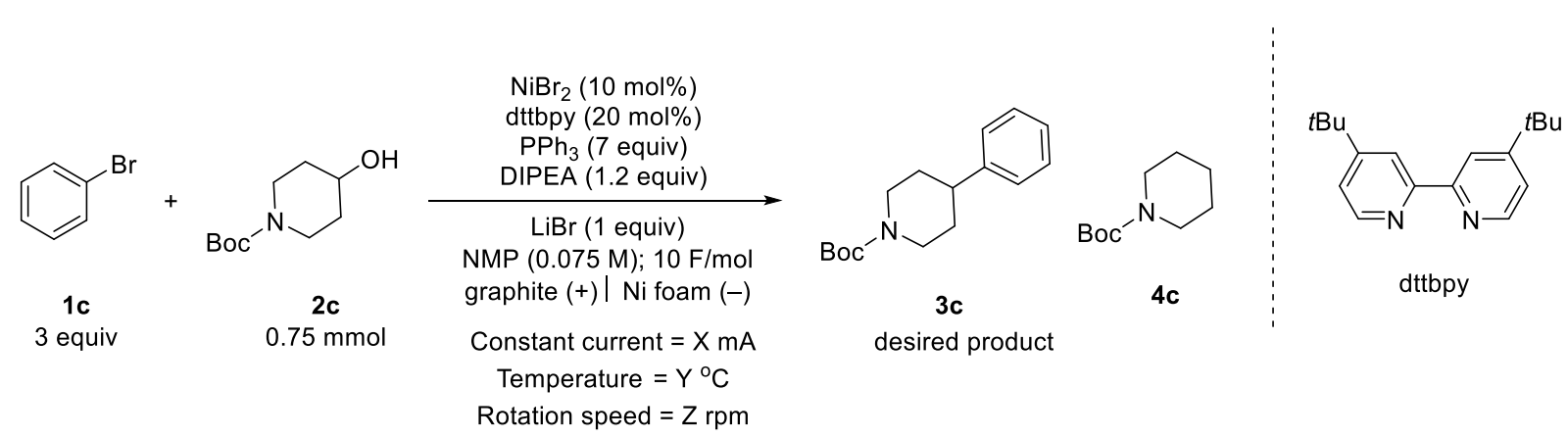

**Table S17** The crude $^{1}$H NMR spectroscopic yield of reaction

| Run order | Temperature (°C) | Current (mA) | Rotation rate (rpm) | **3c** (%) |
|---|---|---|---|---|
| 1 | 15 | 15 | 100 | 56 |
| 2 | 45 | 15 | 100 | 67 |
| 3 | 15 | 50 | 100 | 58 |
| 4 | 45 | 50 | 100 | 35 |
| 5 | 15 | 15 | 300 | 38 |
| 6 | 45 | 15 | 300 | 58 |
| 7 | 15 | 50 | 300 | 29 |
| 8 | 45 | 50 | 300 | 30 |
| 9 | 55 | 33 | 200 | 16 |
| 10 | 10 | 33 | 200 | 2 |
| 11 | 30 | 62 | 200 | 35 |
| 12 | 30 | 10 | 200 | 27 |
| 13 | 30 | 33 | 368 | 19 |
| 14 | 30 | 33 | 50 | 8 |

**Note:** Due to the high viscosity of the system, the trend in yield with respect to temperature and current appears irregular. The highest yield (67%) was obtained at 15 mA and 45 ºC. Additionally, a lower stirring speed was found to be beneficial for improving the reaction yield.

### 3.3.5 3D representation model set-up: Predicted reaction yield of 3c

Reaction yields across the reaction space were predicted via Gaussian process regression and visualized in a 3D plot. The detailed code for generating the 3D plot is provided in an accompanying file (3D plot code.ipynb). Points on the surface represent the variation in yield with respect to two parameters while the third parameter is held at its optimal value, providing insight into yield trends across the examined variable ranges. By integrating the electrochemical set-up with 3D visualization, we can predict reaction yields and optimize the condition screening process, thereby minimizing unnecessary experimental efforts. A 3D model was constructed based on data obtained from the DoE screening to analyze and predict trends in reaction yield. The model explores the relationships between pairs of key variables, including current and temperature, current and rotation rate, and temperature and rotation rate.

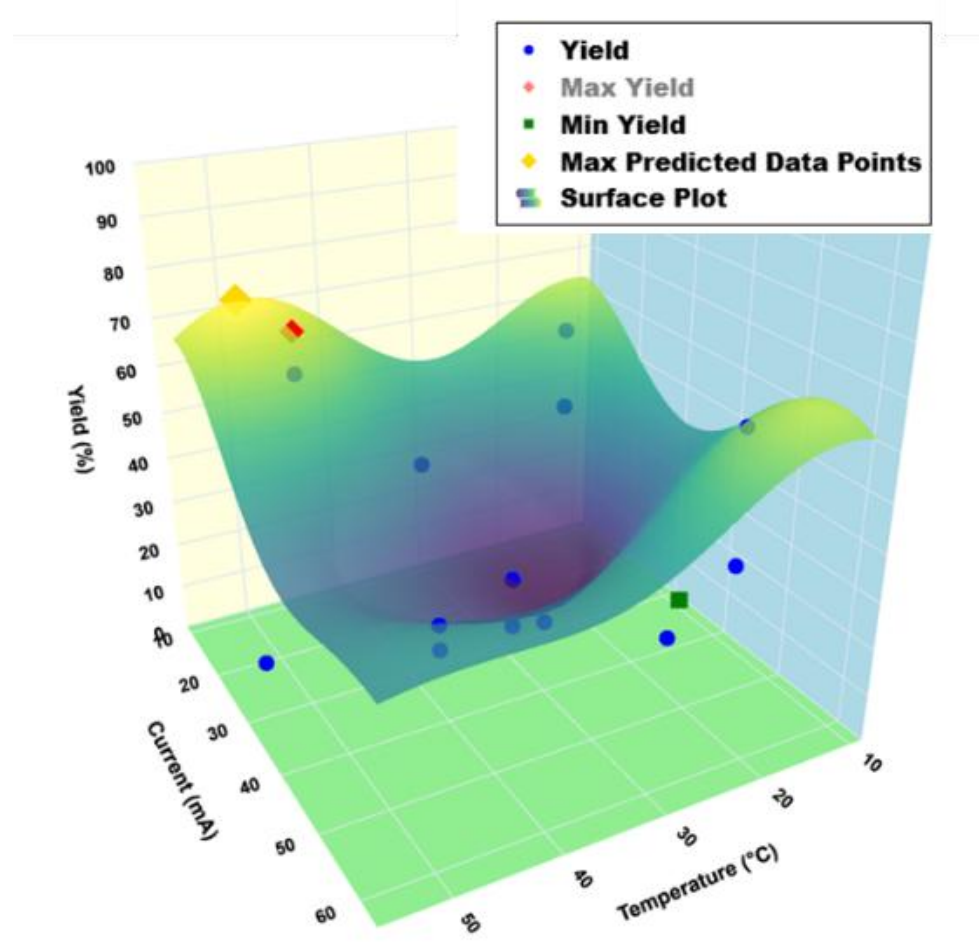


**Figure S24** 3D surface plot of yield vs current and temperature

**Note:** From the 3D plot, it can be seen that the reaction does not show a strong dependence on current, although slightly lower current appears to be favorable. Similarly, the reaction is not strongly affected by temperature, suggesting that the rate-determining step may not be temperature-dependent.

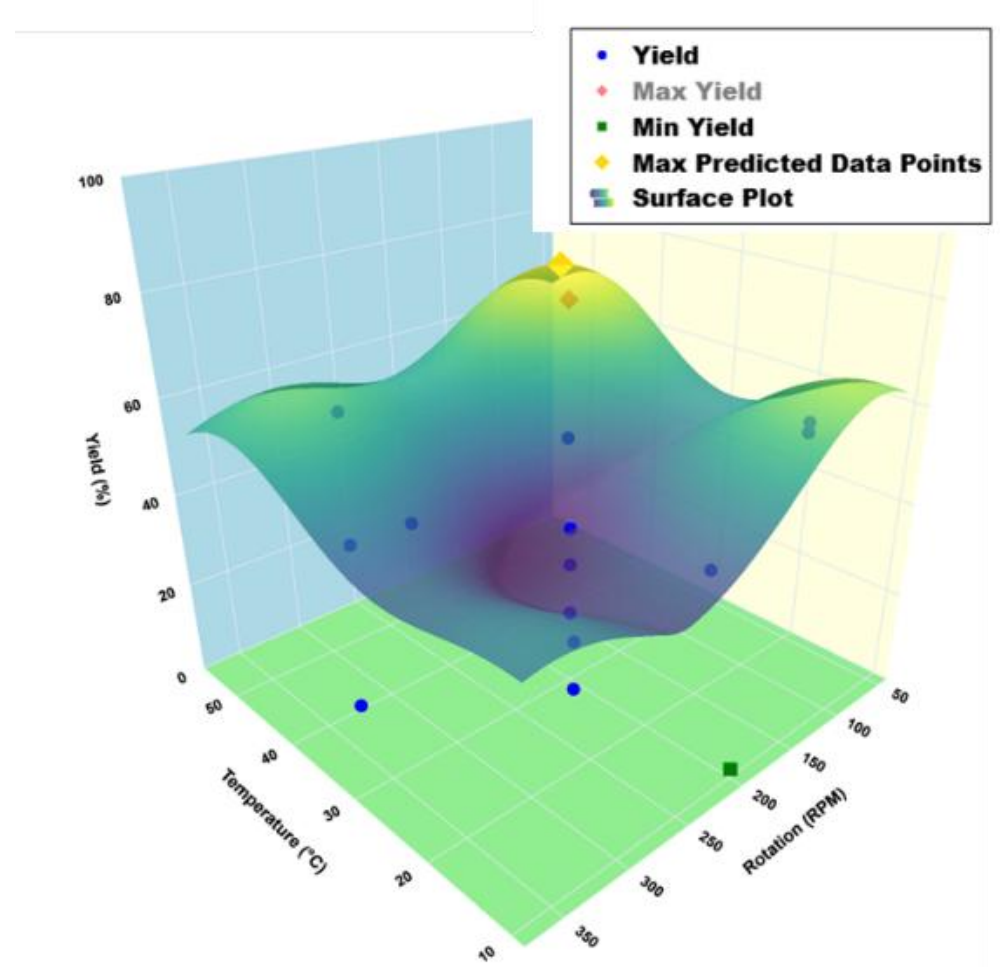


**Figure S25** 3D surface plot of yield vs temperature and rotation rate

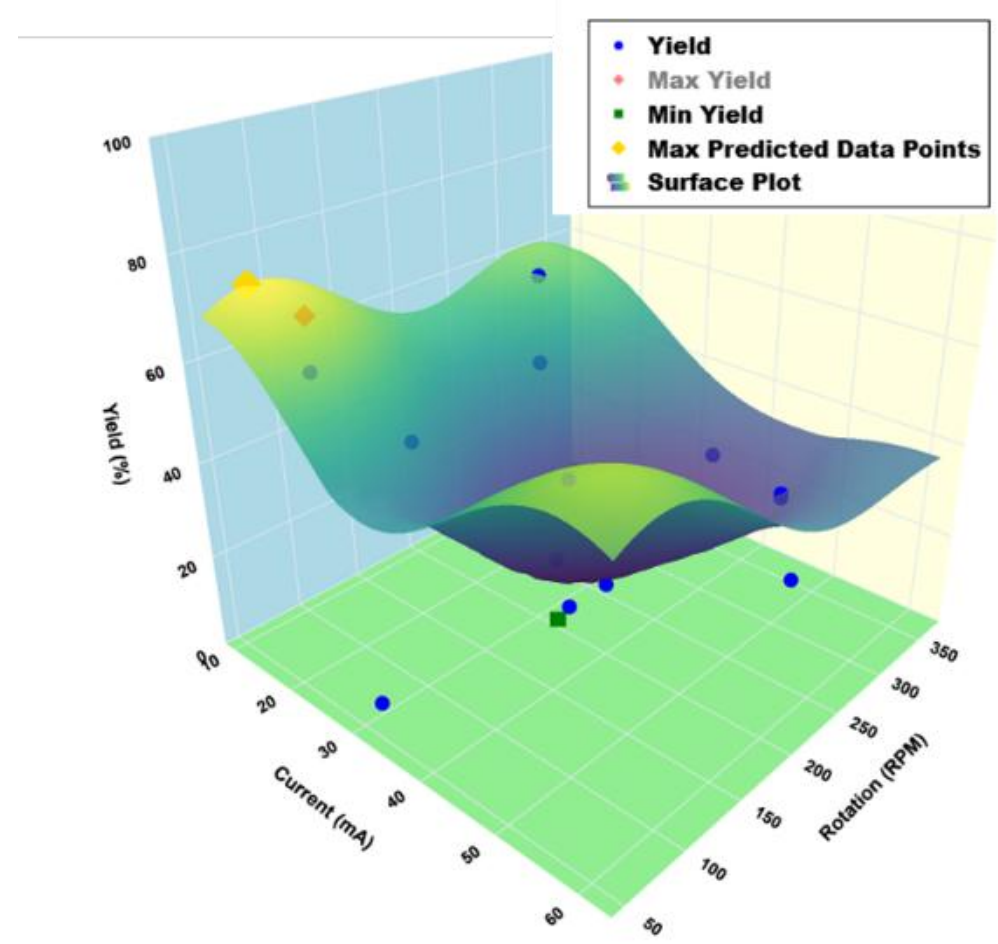


**Figure S26** 3D surface plot of yield vs current and rotation rate

**Note:** A lower stirring speed is beneficial for the reaction, which we attribute to the high viscosity of the solution caused by the presence of 7 equivalents of $PPh_3$. Reduced stirring likely facilitates the formation of the intermediates at the anode and in the reaction solution before their reduction at the cathode, consistent with the mechanism reported in Li's paper.[3]

### 3.3.6 2D contour map representation model set-up: Predicted reaction yield of 3c

To more clearly illustrate the relationship between the two variables under the third optimized condition, a 2D contour map was also constructed. Gaussian process regression was used to generate approximately 20,000 predicted data points based on 14 sets of experimental data. Among the 20,000 predicted data sets, the data corresponding to the other two variables were selected under the optimized condition of the third variable, and a 2D contour map was constructed. The code is provided in the accompanying file (2D_contour map.ipynb). Points on the contour map represent the variation in yield with respect to two parameters while the third parameter is held at its optimal value, providing insight into yield trends across the examined variable ranges. By integrating the electrochemical set-up with a 2D contour map, the changes in yield with variation of the reaction variables can be clearly observed.

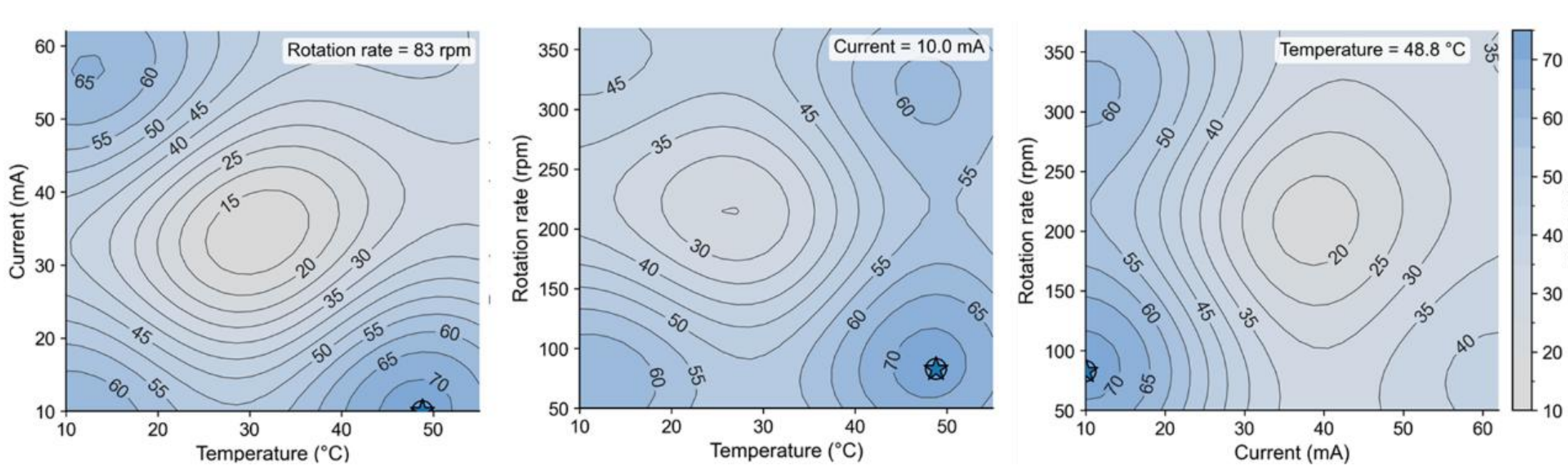


**Figure S27** 2D contour map

### 3.3.7 2D contour map representation model set-up: Predicted productivity of 3c

To more clearly illustrate the relationship between productivity with two variables under the third optimized condition, a 2D contour map was also constructed. Gaussian process regression was used to generate approximately 20,000 predicted data points based on 14 sets of experimental data. Among the 20,000 predicted data sets, the data corresponding to the other two variables were selected under the optimized condition of the third variable, and a 2D contour map was constructed. The code is provided in the accompanying file (2D_contour map.ipynb). Points on the contour map represent the variation in yield with respect to two parameters while the third parameter is held at its optimal value, providing insight into yield trends across the examined variable ranges. By integrating the electrochemical set-up with a 2D contour map, the changes in yield with variation of the reaction variables can be clearly observed. Productivity was estimated from the predicted yield according to the following equation:

$$P = \frac{j \times Y \times M}{5 \times n \times F}$$

where $P$ is the productivity (kg/day m$^2$), $j$ is the current density (A/m$^2$), $Y$ is the predicted yield, $M$ is the molecular weight of the product (g/mol), $F$ is the Faraday constant (96485 C/mol), and n is the number of electrons involved in the transformation, the equation was derived by approximating the Faradaic efficiency as (FE = Y/5), based on the passage of 10 F/mol of charge in all experiments.

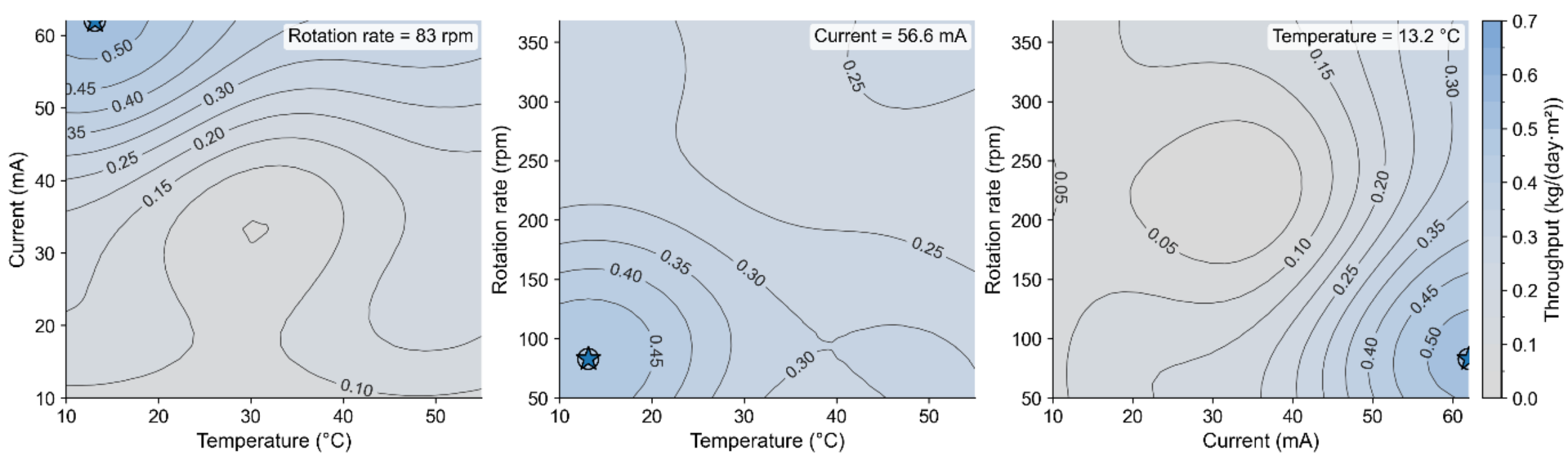


**Figure S28** 2D contour map showing the relationship between throughput and reaction variables

### 3.3.8 System stability test using electrochemical reactions of 80 mL scale

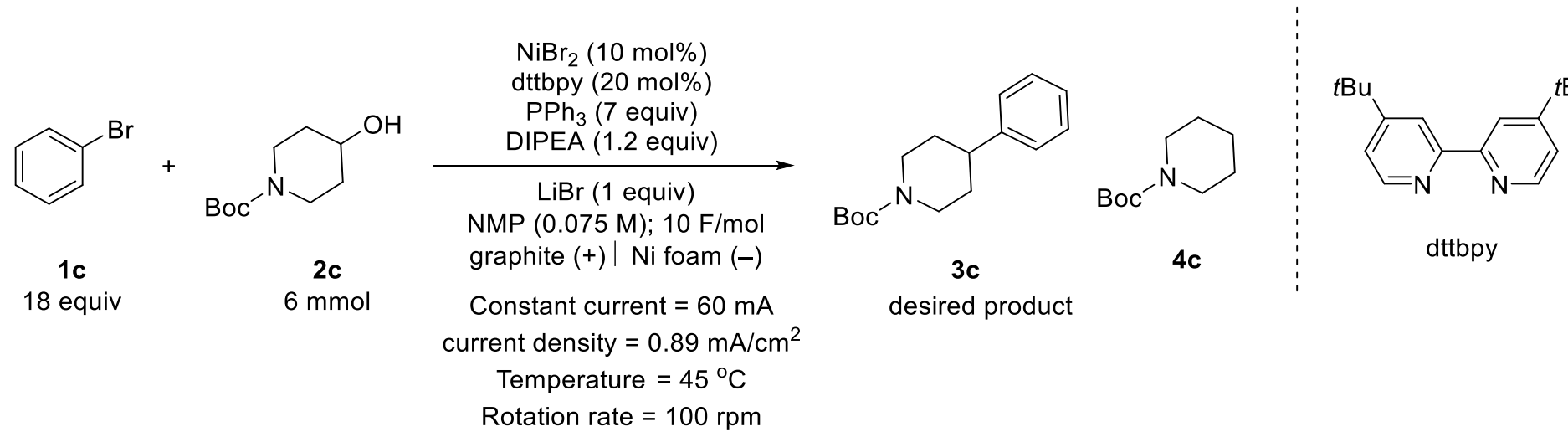

After demonstrating that the platform could successfully perform condition screening, we next evaluated its capability for larger-scale synthesis to further showcase its practical applicability. An 80 mL reaction solution (6.0 mmol substrate, 0.075 M) was prepared in a round-bottom flask according to the procedure described in section 3.3.2 with 1,3,5-trimethoxybenzene (0.50 g, 3.0 mmol, 0.5 equiv relative to the substrate **2c**) was added as internal standard. The flask was sealed with a rubber stopper and placed in the purge box, after which the solution was transferred into the eSTR via a peristaltic pump under an $N_2$ atmosphere. Electrolysis was carried out at a constant current of 60 mA (0.89 mA/cm$^2$) for 26.8 h, while the reaction solution in the flask was maintained at 45 ºC. After completion, the reaction mixture was quenched with $H_2O$. The reaction mixture was filtered through a pad of silica gel which was then washed with a 5:1 mixture of pentane/EtOAc. The combined filtrates were transferred to an NMR tube, and the crude yield was determined by $^1$H NMR spectroscopy using 1,3,5-trimethoxybenzene as the internal standard. The desired product **3c** was obtained in 65% NMR yield (see $^1$H NMR spectrum of the crude reaction product in Section 5). The combined filtrates were concentrated under reduced pressure, and the product was isolated by silica gel flash chromatography (hexane:ethyl acetate = 85:15). The purified product was obtained in 62% isolated yield (1.02 g): $^1$H NMR (400.0 MHz, $CDCl_3$) $\delta$ 7.35–7.28 (m, 2H), 7.24–7.16 (m, $J$ = 8.0 Hz, 3H), 4.24 (br, 2H), 2.82 (d, $J$ = 13.7 Hz, 2H), 2.64 (t, $J$ = 3.5 Hz, 1H), 1.82 (d, $J$ = 13.1 Hz, 2H), 1.64 (td, $J$ = 12.8, 4.2 Hz, 2H), 1.48 (s, 9H); $^{13}$C NMR (100.0 MHz, $CDCl_3$) $\delta$ 154.9, 145.8, 128.5, 126.8, 126.4, 79.4, 42.8, 33.2, 28.5. The spectral NMR are consistent with those reported in the literature.[3]

### 3.4 General procedure for optimizing the nickel-catalyzed electrochemical asymmetric C($sp^2$)–C($sp^3$) reductive cross-coupling of *α*-Cl esters and aryl bromides

#### 3.4.1 Reproduction of the reported procedure

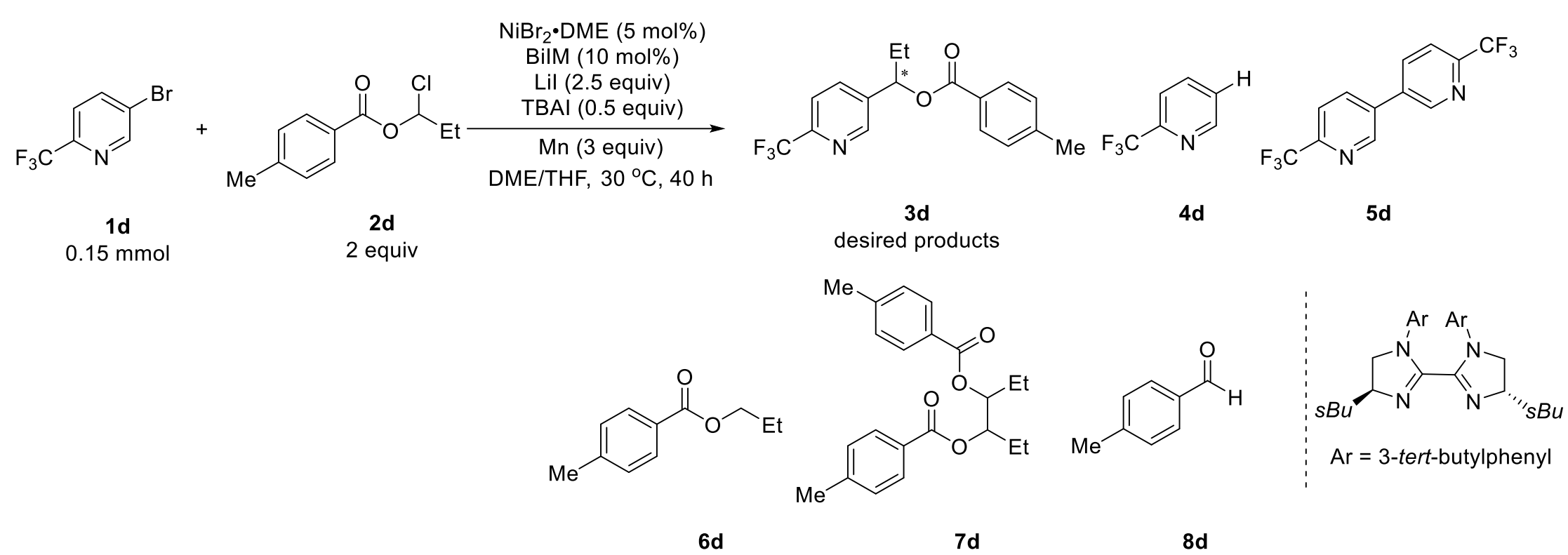


The reaction was reproduced following the reported procedure with minor modifications.[4] A 4 mL vial equipped with a 7 mm magnetic stir bar was dried overnight in an oven at 110 ºC. The hot vial was then transferred to the glovebox antechamber, evacuated, allowed to cool to room temperature, and subsequently brought into the glovebox. $NiBr_2{\cdot}DME$ (2.3 mg, 0.0075 mmol, 5 mol%), ligand (7.7 mg, 0.015 mmol, 9.2

mol%), and 0.9 mL THF/DME (2:1) were added to an oven-dried vial and stirred for 30 min to afford a homogeneous solution. Mn (24.7 mg, 0.45 mmol, 3.0 equiv), LiI (50.2 mg, 2.5 equiv), and TBAI (27.7 mg, 0.5 equiv) were added. After 5 min, 1-chloropropyl 4-methylbenzoate (63.8 mg, 2.0 equiv) was added to the tube by a syringe over 1 min. The mixture was stirred vigorously at 30 ºC for 40 h. A solution of $CH_2Br_2$ in $CHCl_3$ (100 μL, 1.0 M) was added as an internal standard, and the mixture was stirred in air for an additional 5 min. An aliquot (100 μL) of the reaction mixture was withdrawn and passed through a short pad of silica gel, which was subsequently washed with $CDCl_3$ (3 × 200 μL). The combined filtrates were transferred to an NMR tube, and the crude yield was determined by $^1$H NMR spectroscopy using $CH_2Br_2$ as the internal standard. Under these conditions, the desired product **3a** was obtained in 56% NMR yield.

### 3.4.2 Batch condition screening for Ni-catalyzed electrochemical asymmetric C($sp^2$)–C($sp^3$) reductive cross-coupling of *α*-chloro esters with aryl bromides

**General Procedure**: A test tube (16 × 100 mm) equipped with a 10 mm cross-shaped magnetic stir bar was dried overnight in an oven at 110 ºC. The hot test tube was then transferred to the glovebox antechamber, evacuated, allowed to cool to room temperature, and subsequently brought into the glovebox. $NiBr_2$·DME (24.6 mg, 0.08 mmol, 20 mol%), $^{iPr}$BiIM (46.7 mg, 0.096 mmol, 24 mol%) or $^{sBu}$BiIM ligand (49.3 mg, 0.096 mmol, 24 mol%), and 1.8 mL THF/DME (2:1) were added to an oven-dried 10 mL test tube and stirred for 30 min to afford a homogeneous solution. LiI (134.0 mg, 1.0 mmol, 2.5 equiv), TBAI (74.0 mg, 0.20 mmol), $TBAPF_6$ (154.8 mg), aryl bromide **1d** (89.6 mg, 0.40 mmol, 1.0 equiv), and *α*-chloro esters **2d** (170.0 mg, 0.8 mmol, 2.0 equiv) were then added sequentially. The reaction vessel was sealed with the electrode cap assembly. The reaction mixture was electrolyzed at rt under vigorous stirring (1500 rpm) at a constant current of −2 mA for 16 h using Ni foam as the cathode and Zn plate as the anode. Upon completion, the reaction vessel was removed from the glovebox and quenched with $H_2O$ (200 μL). A solution of $CH_2Br_2$ in $CHCl_3$ (100 μL, 1.0 M) was added as an internal standard, and the mixture was stirred for an additional 5 min. An aliquot (100 μL) of the reaction mixture was withdrawn and passed through a short pad of silica gel, which was subsequently washed with $CDCl_3$ (3 × 200 μL). The combined filtrates were transferred to an NMR tube, and the crude yield was determined by $^1$H NMR spectroscopy using 1,3,5-trimethoxybenzene as the internal standard.

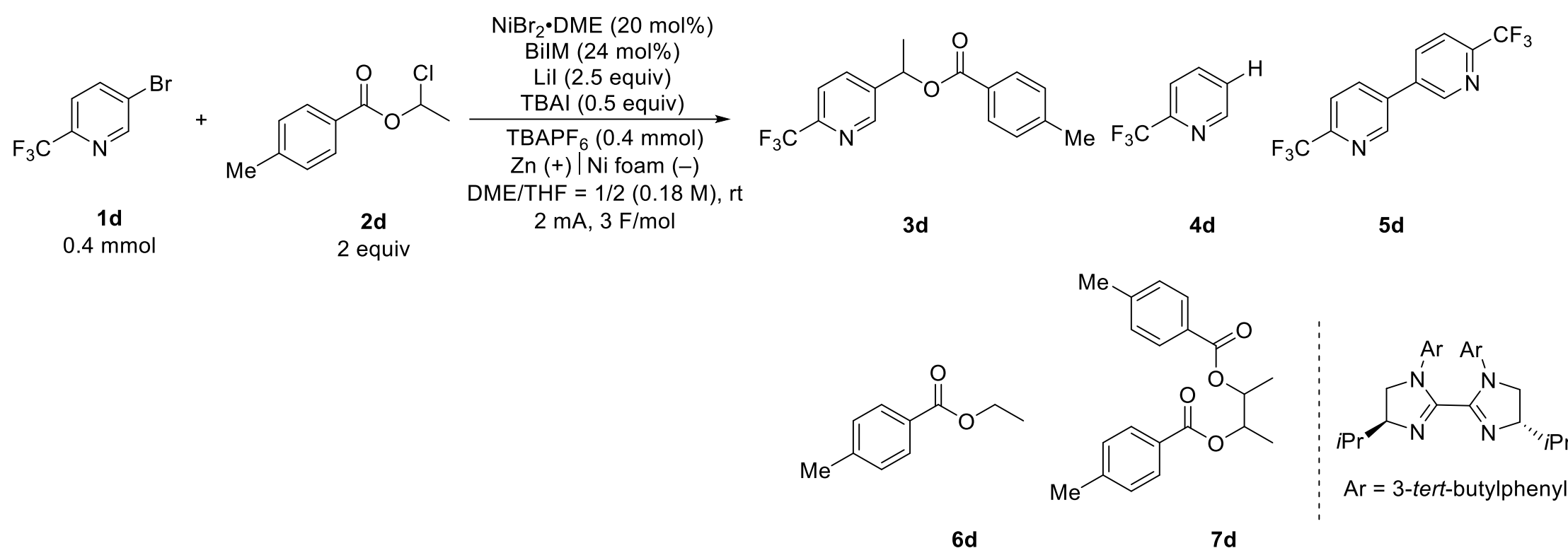


**Table S18** Condition optimization of the electrochemical asymmetric reductive coupling (Part 1)

| Entry | Deviation from Standard Conditions | Yield of **3d**[a] (%) | Yield of **4d/5d/6d** (%) | Recovery of **1d/2d** (%) |
|---|---|---|---|---|
| 1 | None | 27 | 5/34/20 | -/116 |
| 2 | 3 mA instead of 2 mA | 35 | 3/28/10 | 10/117 |
| 3 | $ZnCl_2$ (0.5 equiv) as additive | 18 | 15/22/25 | -/127 |
| 4 | DME/THF = 1/2 (0.1 M) instead of 0.2 M | 8 | -/33/15 | -/80 |
| 5 | DME/THF = 1/2 (0.06 M) instead of 0.2 M | - | -/46/12 | -/88 |
| 6 | 10 ºC instead of rt | - | -/45/11 | -/150 |
| 7 | Mg (+) instead of Zn (+) | - | - | - |
| 8 | 3 mA; 4 F/mol and 0.2 M | 47 | 10/23/14 | -/30 |

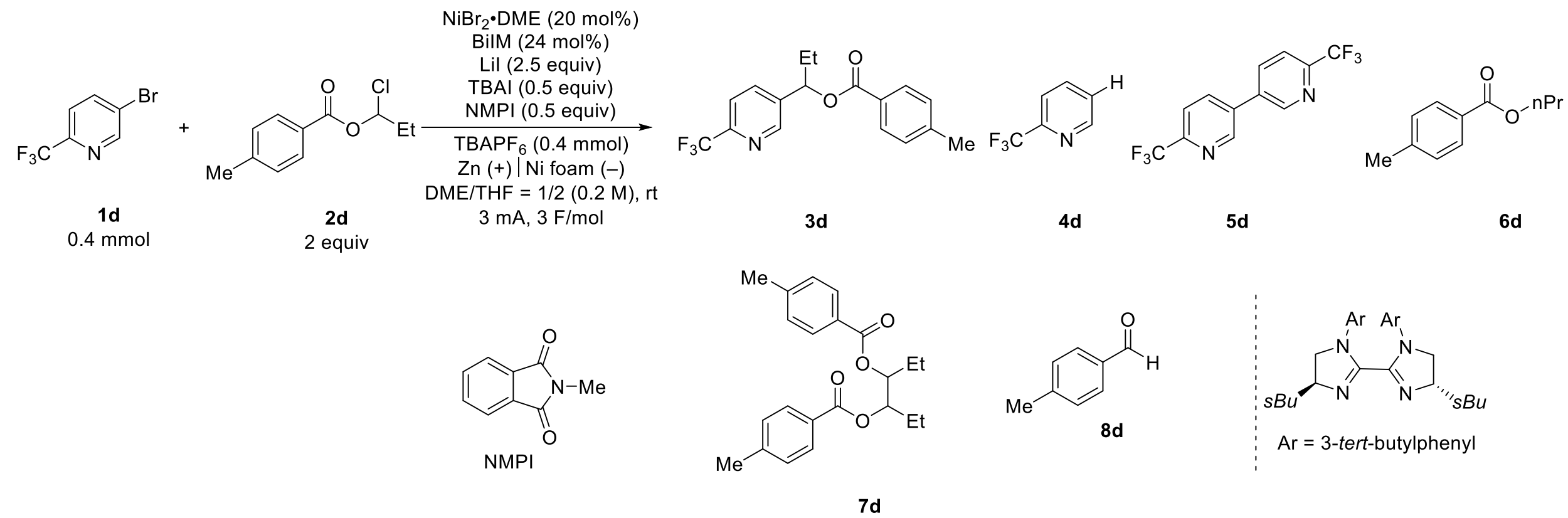

**Table S19** Condition optimization of the electrochemical asymmetric reductive coupling (Part **2**)

| Entry | Deviation from Standard Conditions | Yield of **3d**[a] (%) | Yield of **4d/5d/6d/8d** (%) | Recovery of **1d/2d** (%) |
|---|---|---|---|---|
| 1[a] | None | 54 | 5/18/12/7 | -/28 |
| 2 | 0.6 mmol **1a;** w/o NMPI | 57 | 6/11/17/21 | 3/49 |
| 3 | 0.8 mmol **1a;** w/o NMPI | 39 | 6/17/12/20 | 12/34 |

### 3.4.3 Preparation of the reaction solution for condition screen in the eSTR

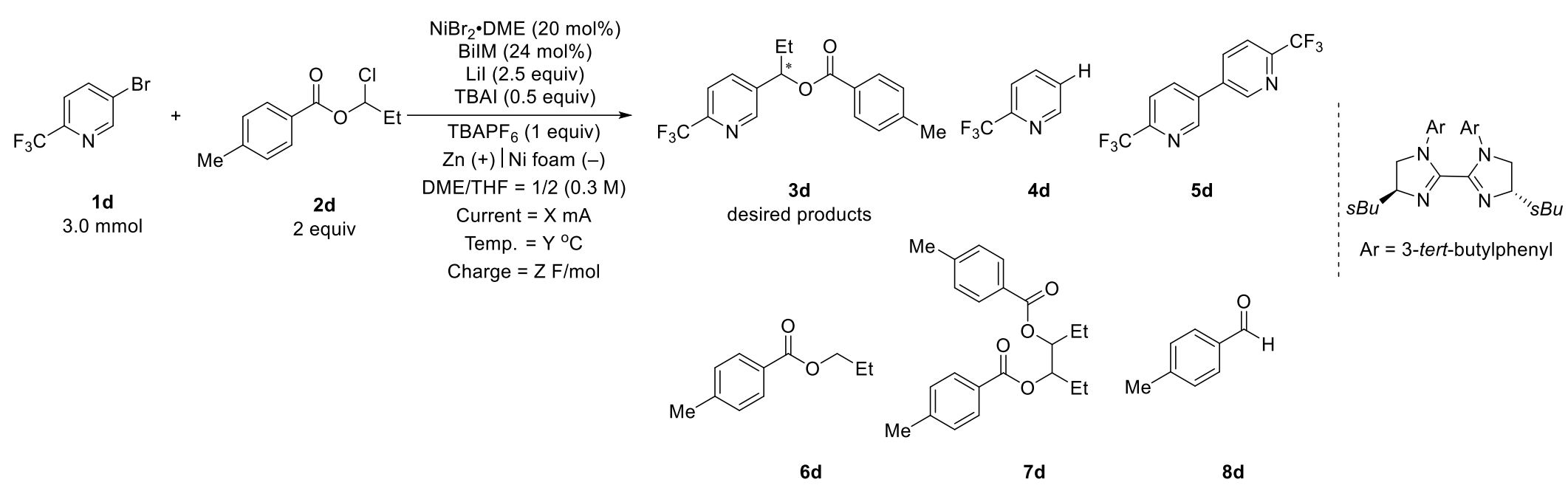

A 250 mL round-bottom flask equipped with a cross-shaped magnetic stir bar was dried in an oven at 110 ºC overnight. The hot flask was transferred to the glovebox antechamber, evacuated, and allowed to cool to room temperature before being brought into the glovebox. $NiBr_2 \cdot$DME (6.0 mmol, 1.85 g), $^{sBu}$BiIM (7.2 mmol, 3.71 g), and THF (60 mL) were added sequentially. The resulting suspension was stirred at 50 ºC for 30 min. Subsequently, $TBAPF_6$ (30.0 mmol, 11.62 g), 5-bromo-2-(trifluoromethyl)pyridine (30.0 mmol, 6.75 g), 1-chloropropyl 4-methylbenzoate (60.0 mmol, 11.88 g), TBAI (15.0 mmol, 5.52 g), LiI (75.0 mmol, 9.98 g), and DME (30 mL) were added in order. Because of the limited solubility of the reaction mixture, the solution was divided evenly into four 40 mL vials. Each vial was sealed with a rubber stopper, transferred into the purge box, and placed on a simple magnetic stirring device to maintain uniform agitation and ensure homogeneous mixing throughout the reaction.

**Note***:* Due to the poor solubility of the starting material, the reaction mixture was divided into four portions and kept under continuous stirring to prevent clogging when being pumped into the eSTR.

### 3.4.4 Set-up of reaction conditions

Three parameters—current, temperature, and charge—were selected to evaluate their effect on the reaction. The ranges of these variables were chosen based on the condition screening reported in Chen's paper. Since the reaction is sensitive to current density, the current density was varied from 0.89 to 1.49 mA/cm$^2$ (When the reaction volume was 10 mL, the immersed surface area of the Ni foam electrode was 1.2 cm$^2$ × 14. Therefore, the applied current corresponds to a current density of X mA/16.8 cm$^2$. and currents of 15–25 mA). The temperature range was set from 25 ºC to 30 ºC, and the charge was varied from 2.5 to 3 F/mol. A Design of Experiments (DoE) approach was employed to generate 8 reaction runs, as summarized below. The conditions were slightly adjusted during the actual experiments to accommodate operational requirements.

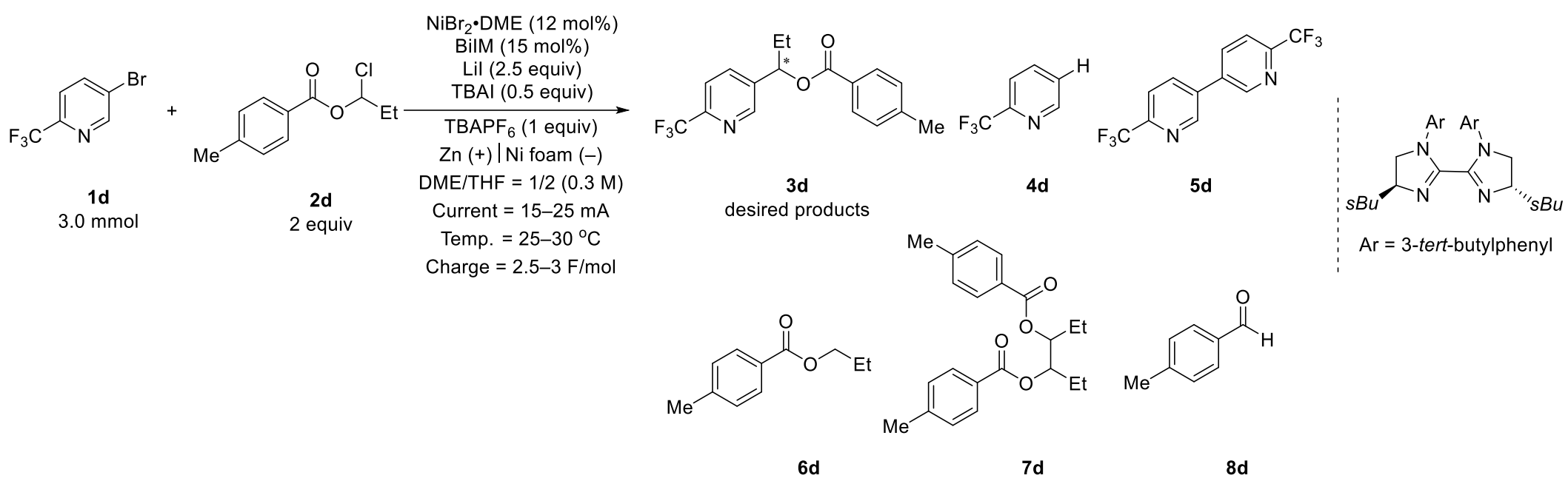


**Table S20** Variable ranges of the factors

| Factor | Lower | Higher |
|---|---|---|
| Temperature | 25 ºC | 30 ºC |
| Current | 15 mA | 25 mA |
| Charge | 2.5 F/mol | 3 F/mol |

**Table S21** Theoretical design of condition screening via DoE

| Run order | Temperature (ºC) | Current (mA) | Charge (F/mol) |
|---|---|---|---|
| 1 | 25 | 15 | 2.5 |
| 2 | 30 | 15 | 2.5 |
| 3 | 25 | 25 | 2.5 |
| 4 | 30 | 25 | 2.5 |
| 5 | 25 | 15 | 3 |
| 6 | 30 | 15 | 3 |
| 7 | 25 | 25 | 3 |
| 8 | 30 | 25 | 3 |

**Note:** this table was automatically generated by the DoE based on the three selected parameters ((https://numiqo.com/statistics-calculator/design-of-experiments). Some values in the table may be unrealistic and require adjustment to align with practical screening conditions. The DoE-generated table provides an organized framework for positioning data points, which facilitates the subsequent application

of Gaussian process regression to construct a more accurate 3D responses. Due to the temperature sensitivity of the asymmetric reaction, we screened only the temperature range of 25–30 ºC based on the conditions reported in Chen's paper for the chemical version of the reaction.[4]

### 3.4.5 Results analysis

After the reaction is completed, 75 µL aliquot of the product solution was taken and passed through a short silica gel column in a pipette, eluting with $CDCl_3$ (3 × 300 µL). The resulting filtrate was then analyzed by NMR. Due to the high viscosity of the system, the trend in yield with respect to temperature and current appears irregular. The highest yield (57%) was obtained at 25 mA, 30 ºC, and 3 F/mol.

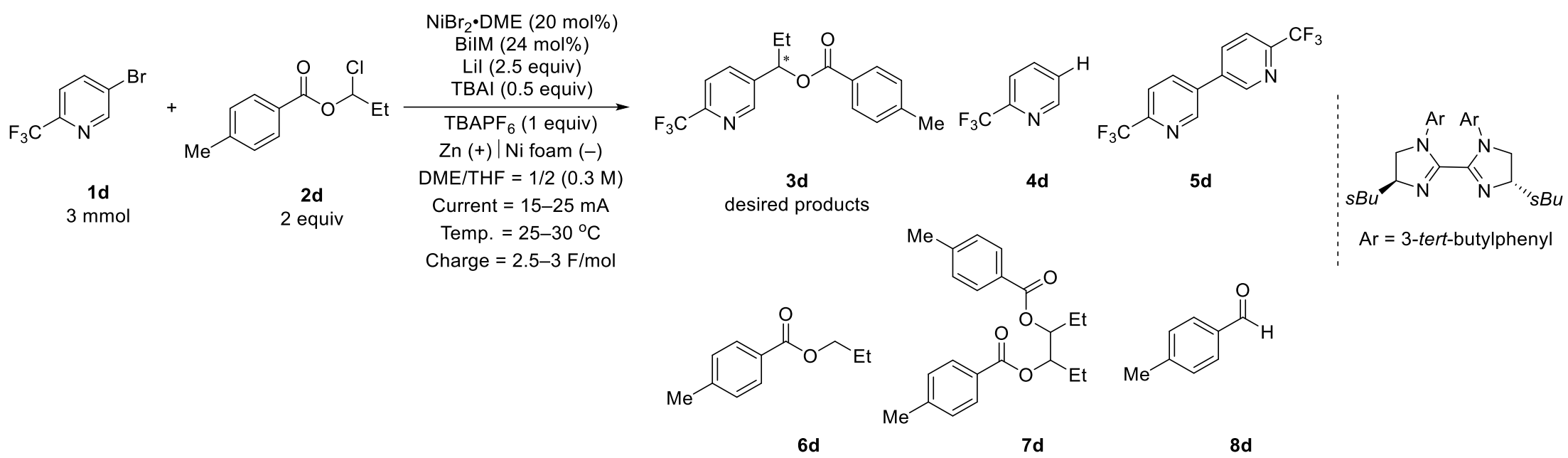


**Table S22** The crude $^{1}$H NMR spectroscopic data of reaction

| Run order | Temperature (ºC) | Current (mA) | Charge (F/mol) | **3d** (%) | ***ee*** (%) |
|---|---|---|---|---|---|
| 1 | 25 | 15 | 2.5 | 35 | 88 |
| 2 | 30 | 15 | 2.5 | 40 | 88 |
| 3 | 25 | 25 | 2.5 | 47 | 88 |
| 4 | 30 | 25 | 2.5 | 52 | 88 |
| 5 | 25 | 15 | 3 | 39 | 89 |
| 6 | 30 | 15 | 3 | 50 | 89 |
| 7 | 25 | 25 | 3 | 46 | 88 |
| 8 | 30 | 25 | 3 | 57 | 88 |

**Note**: the ee value was determined by Chiral SFC: (ID-H, 3.0 mL/min, 10% IPA in $CO_2$, λ = 210 nm): $t_R$ (major) = 1.7 min, $t_R$ (minor) = 2.2 min. Due to the presence of insoluble species that compromised stable operation, the Ni foam electrode was replaced with a new one after the fourth run.

### 3.4.6 System stability test using electrochemical reactions of 80 mL scale

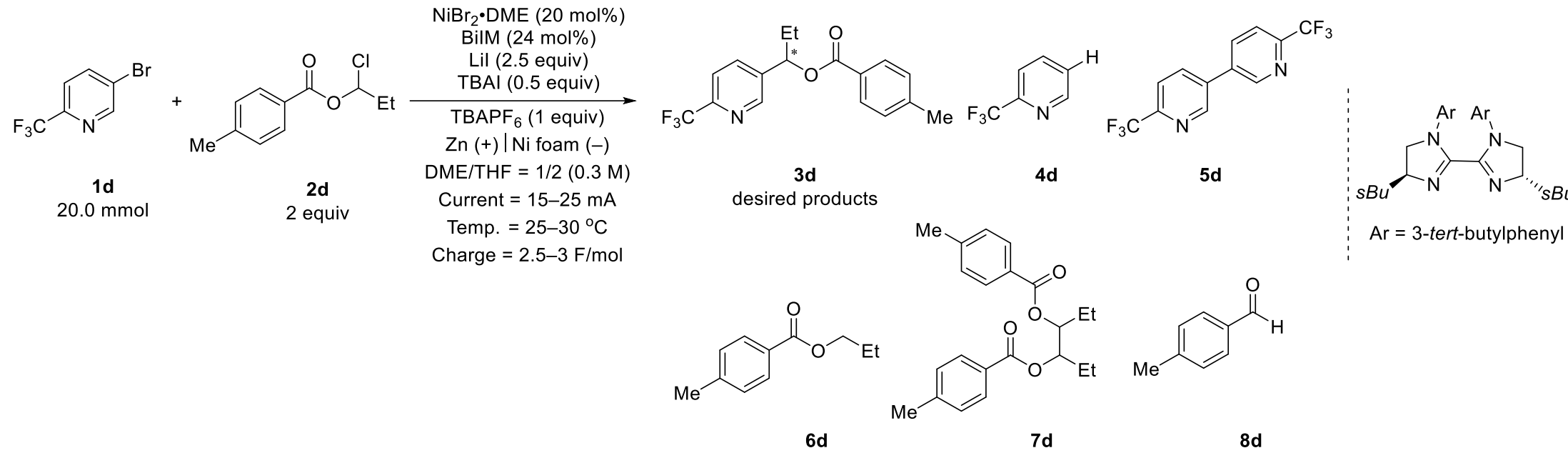


After demonstrating that the platform could successfully perform condition screen, we next evaluated its capability for larger-scale synthesis to further demonstrate its practical applicability. A 70 mL reaction solution (20 mmol substrate, 0.3 M) was prepared in the stir tank under $N_2$ atmosphere according to the procedure described in section 3.4.2. Electrolysis was carried out at a constant current of 120 mA (1.49 mA/cm$^2$) for 13.4 hours, with the reaction solution in the flask maintained at 30 ºC. After completion, the reaction mixture was quenched with $H_2O$, and $CH_2Br_2$ (40 mmol, 700 µL, 2 equiv relative to the substrate **1d**) was added. The reaction mixture was filtered through a pad of silica gel which was then washed with a 5:1 mixture of pentane/EtOAc. The combined filtrates were transferred to an NMR tube, and the crude yield was determined by $^1$H NMR spectroscopy using $CH_2Br_2$ as the internal standard. The desired product **3d** was obtained in 52% NMR yield (see $^1$H NMR spectrum of the crude reaction product in Section 5). The combined filtrates were concentrated under reduced pressure, and the product was isolated by silica gel flash chromatography (hexane:ethyl acetate = 80:20). The purified product was obtained in 50% isolated yield (3.09 g): $^1$H NMR: (400.0 MHz, $CDCl_3$) $\delta$ 8.81 (d, $J$ = 2.1 Hz, 1H), 8.00–7.93 (m, 2H), 7.91 (dd, $J$ = 8.1, 2.1 Hz, 1H), 7.69 (d, $J$ = 8.1, 1H), 7.28 (d, $J$ = 7.9 Hz, 2H), 6.00 (dd, $J$ = 7.5, 6.0 Hz, 1H), 2.44 (s, 3H), 2.19–2.09 (m, 1H), 2.07–1.98 (m, 1H), 1.01 (t, $J$ = 7.4 Hz, 3H). $^{13}$C NMR: (100.0 MHz, $CDCl_3$) $\delta$ 165.7, 148.4, 147.6 (q, $J$ = 34.8 Hz), 144.2, 139.6, 135.3, 129.7, 129.2, 126.9, 121.4 (d, $J$ = 274.1 Hz), 120.3 (q, $J$ = 3.0 Hz), 74.7, 29.4, 21.7, 9.7. Chiral SFC: (AH-H, 3.0 mL/min, 10% IPA in $CO_2$, $\lambda$ = 210 nm): $t_R$ (major) = 1.7 min, $t_R$ (minor) = 2.2 min. The spectral NMR are consistent with those reported in the literature.[4]

## 4. References


(1) Franke, M. C.; Longley, V. R.; Rafiee, M.; Stahl, S. S.; Hansen, E. C.; Weix, D. J. Zinc-Free, Scalable Reductive Cross-Electrophile Coupling Driven by Electrochemistry in an Undivided Cell. *ACS Catal.* **2022**, *12* (20), 12617–12626. https://doi.org/10.1021/acscatal.2c03033.

(2) Jiao, K.; Liu, D.; Ma, H.; Qiu, H.; Fang, P.; Mei, T. Nickel-Catalyzed Electrochemical Reductive Relay Cross-Coupling of Alkyl Halides to Aryl Halides. *Angew Chem Int Ed* **2020**, *59* (16), 6520–6524. https://doi.org/10.1002/anie.201912753.

(3) Li, Z.; Sun, W.; Wang, X.; Li, L.; Zhang, Y.; Li, C. Electrochemically Enabled, Nickel-Catalyzed Dehydroxylative Cross-Coupling of Alcohols with Aryl Halides. *J. Am. Chem. Soc.* **2021**, *143* (9), 3536–3543. https://doi.org/10.1021/jacs.0c13093.

(4) Sun, D.; Tao, X.; Ma, G.; Wang, J.; Chen, Y. Asymmetric Synthesis of Aryl/Vinyl Alkyl Carbinol Esters *via* Ni-Catalyzed Reductive Arylation/Vinylation of 1-Chloro-1-Alkanol Esters. *Chem. Sci.* **2022**, *13* (28), 8365–8370. https://doi.org/10.1039/D2SC02806A.

## 5. NMR Spectra

Note: Crude $^1$H NMR spectra of products from large-scale reactions are included, together with $^1$H and $^{13}$C NMR spectra of isolated products.

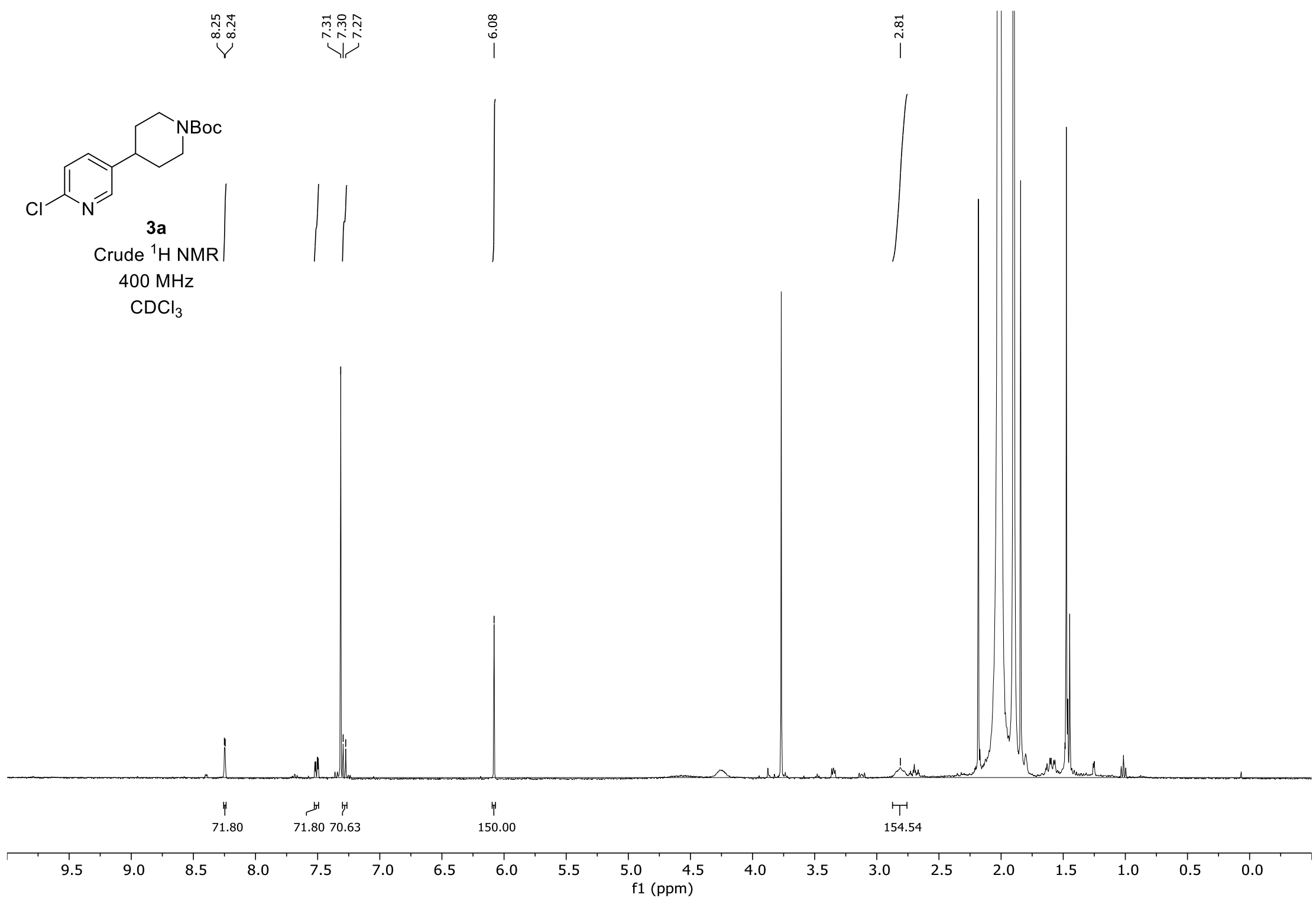

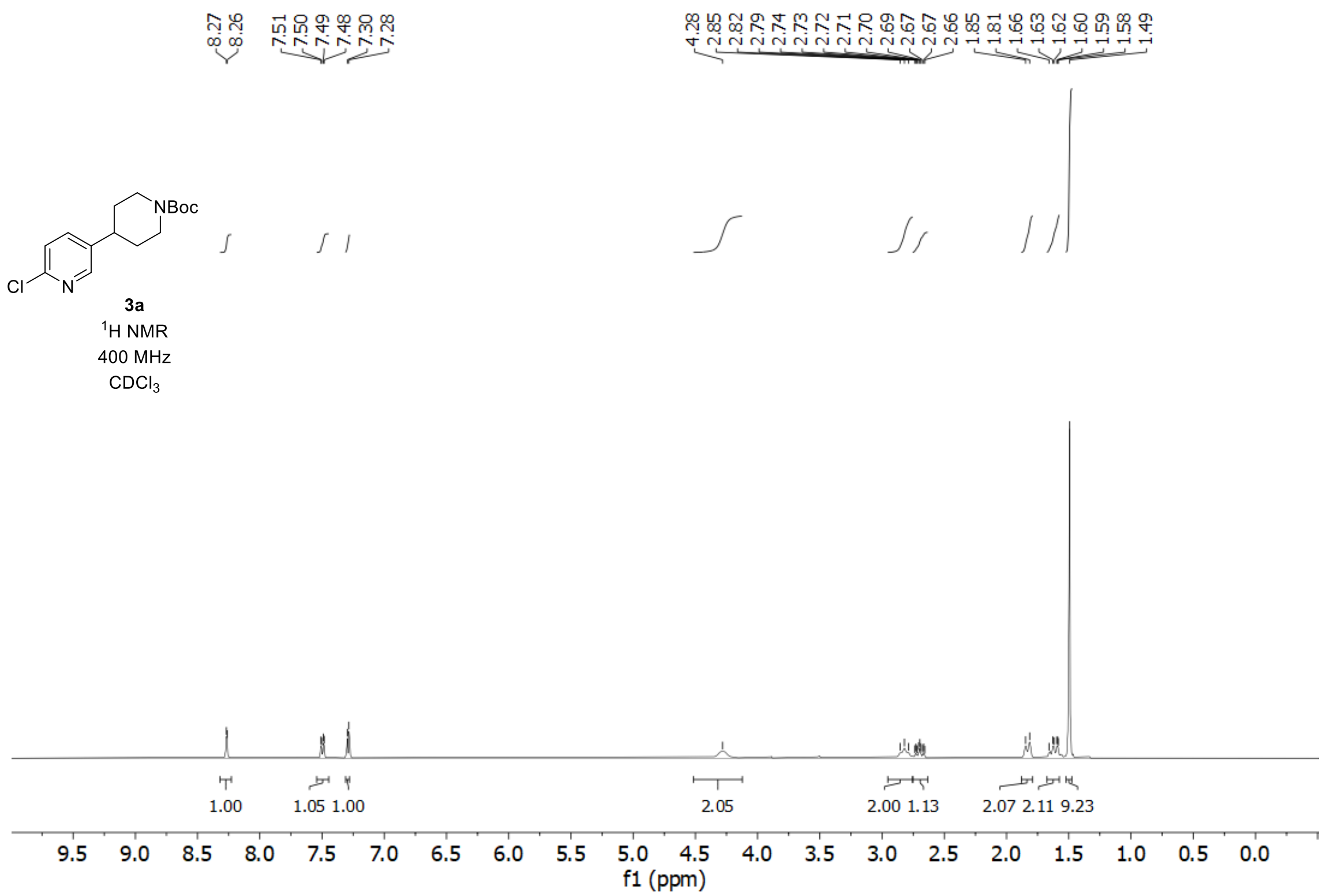
8.27
8.26
7.51
7.50
7.49
7.48
7.30
7.28
4.28
2.85
2.82
2.79
2.74
2.73
2.72
2.71
2.70
2.69
2.67
2.67
2.66
1.85
1.81
1.66
1.63
1.62
1.60
1.59
1.58
1.49
NBoc
Cl
N
3a
1H NMR
400 MHz
CDCl3
1.00
1.05 1.00
2.05
2.00 1.13
2.07 2.11 9.23
9.5 9.0 8.5 8.0 7.5 7.0 6.5 6.0 5.5 5.0 4.5 4.0 3.5 3.0 2.5 2.0 1.5 1.0 0.5 0.0
f1 (ppm)

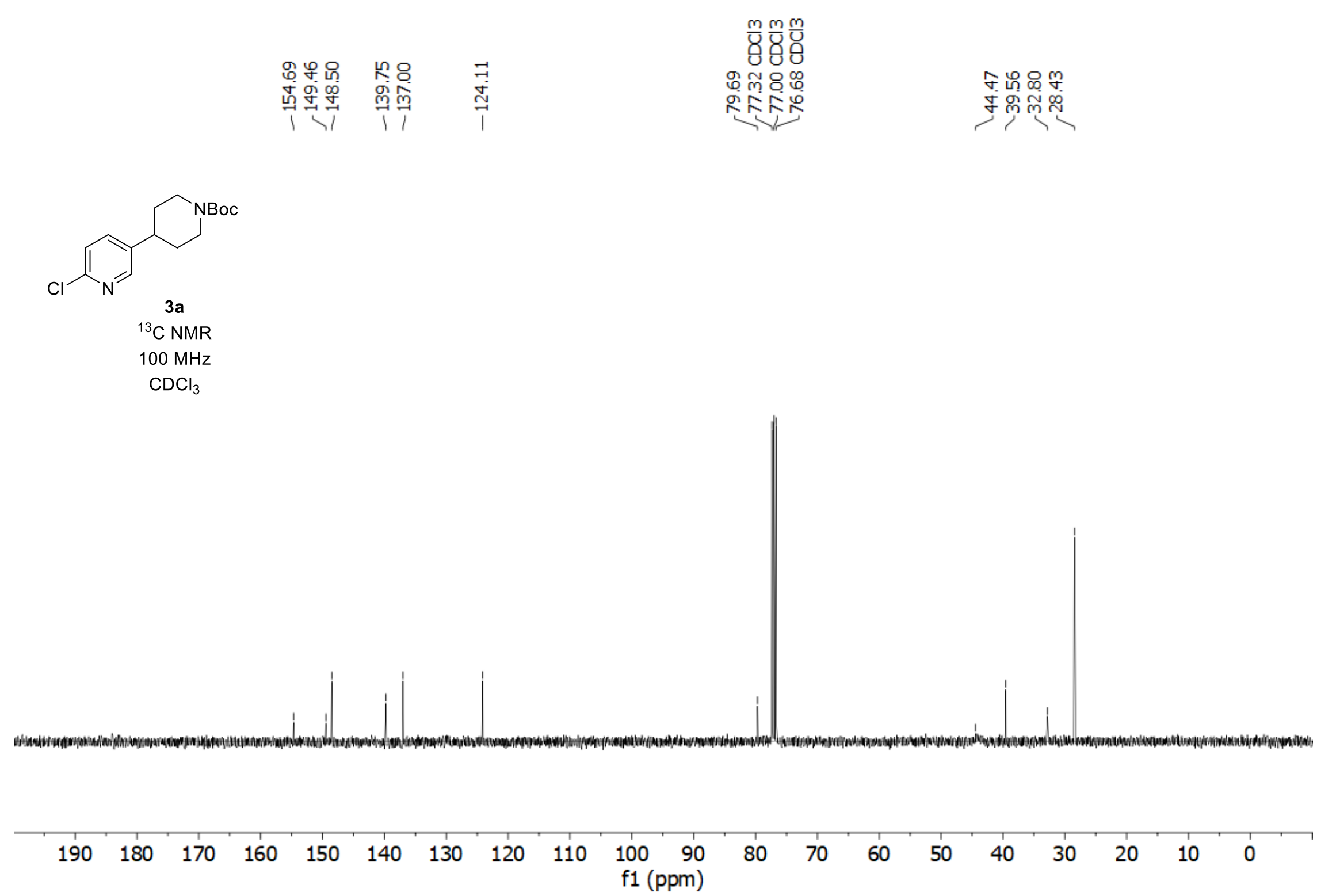
154.69
149.46
148.50
139.75
137.00
124.11
79.69
77.32 CDCl3
77.00 CDCl3
76.68 CDCl3
44.47
39.56
32.80
28.43
NBoc
Cl
N
3a
13C NMR
100 MHz
CDCl3
190 180 170 160 150 140 130 120 110 100 90 80 70 60 50 40 30 20 10 0
f1 (ppm)

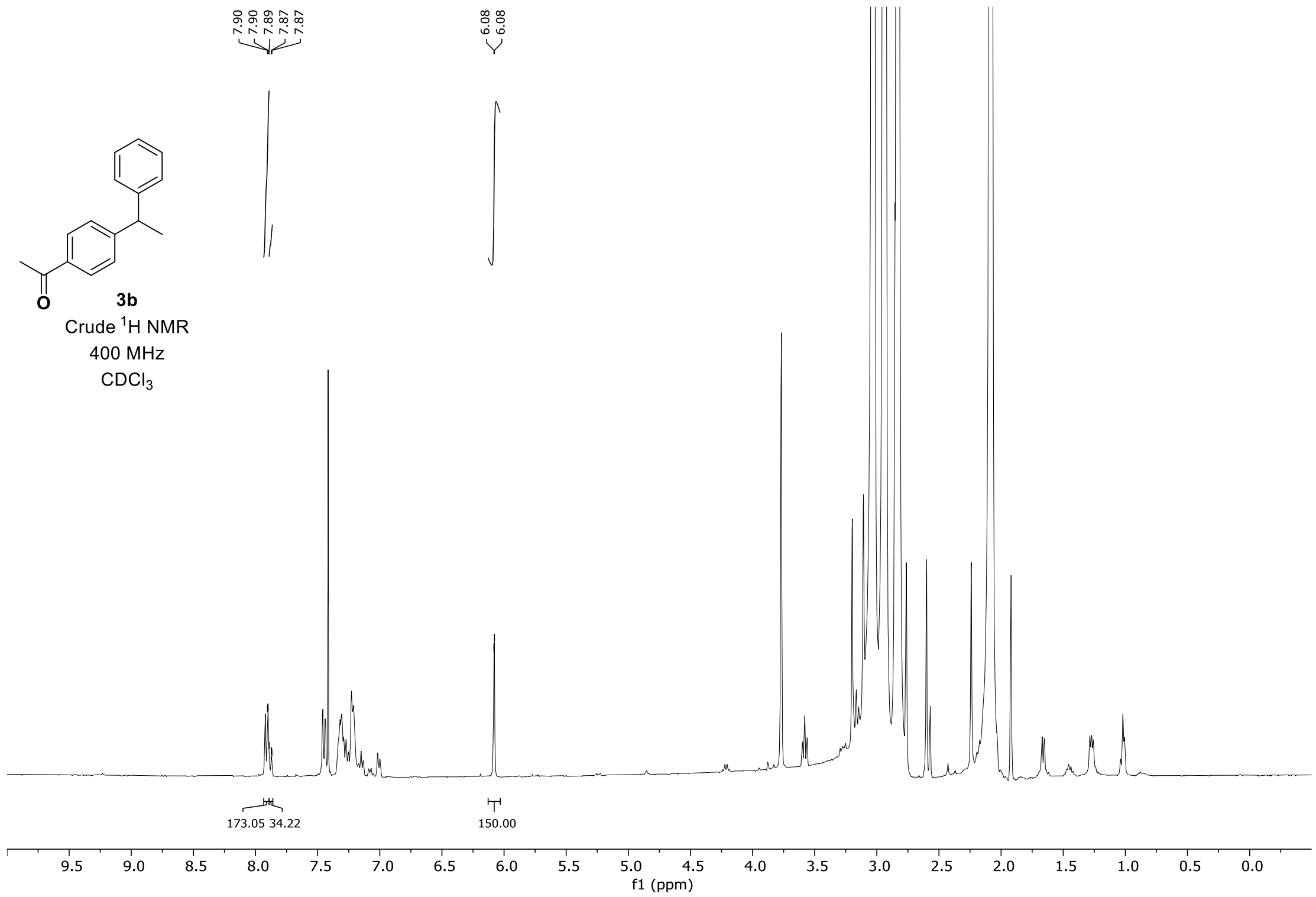
7.90
7.90
7.89
7.87
7.87
6.08
6.08
O
3b
Crude 1H NMR
400 MHz
CDCl3
173.05
34.22
150.00
9.5
9.0
8.5
8.0
7.5
7.0
6.5
6.0
5.5
5.0
4.5
4.0
3.5
3.0
2.5
2.0
1.5
1.0
0.5
0.0
f1 (ppm)

**3b**
$^{1}H$ NMR
400 MHz
$CDCl_3$

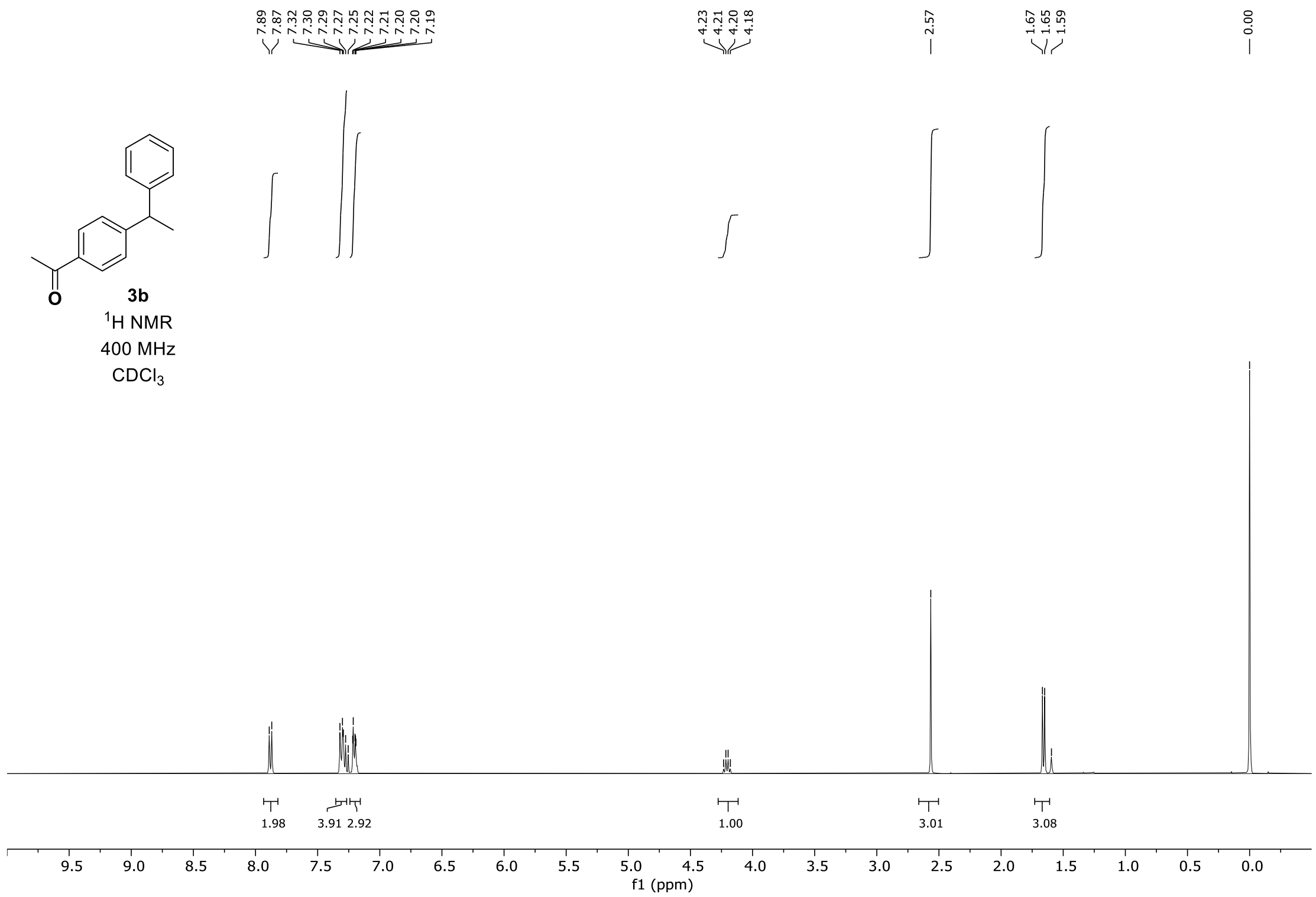


**3b**
$^{13}C$ NMR
100 MHz
$CDCl_3$

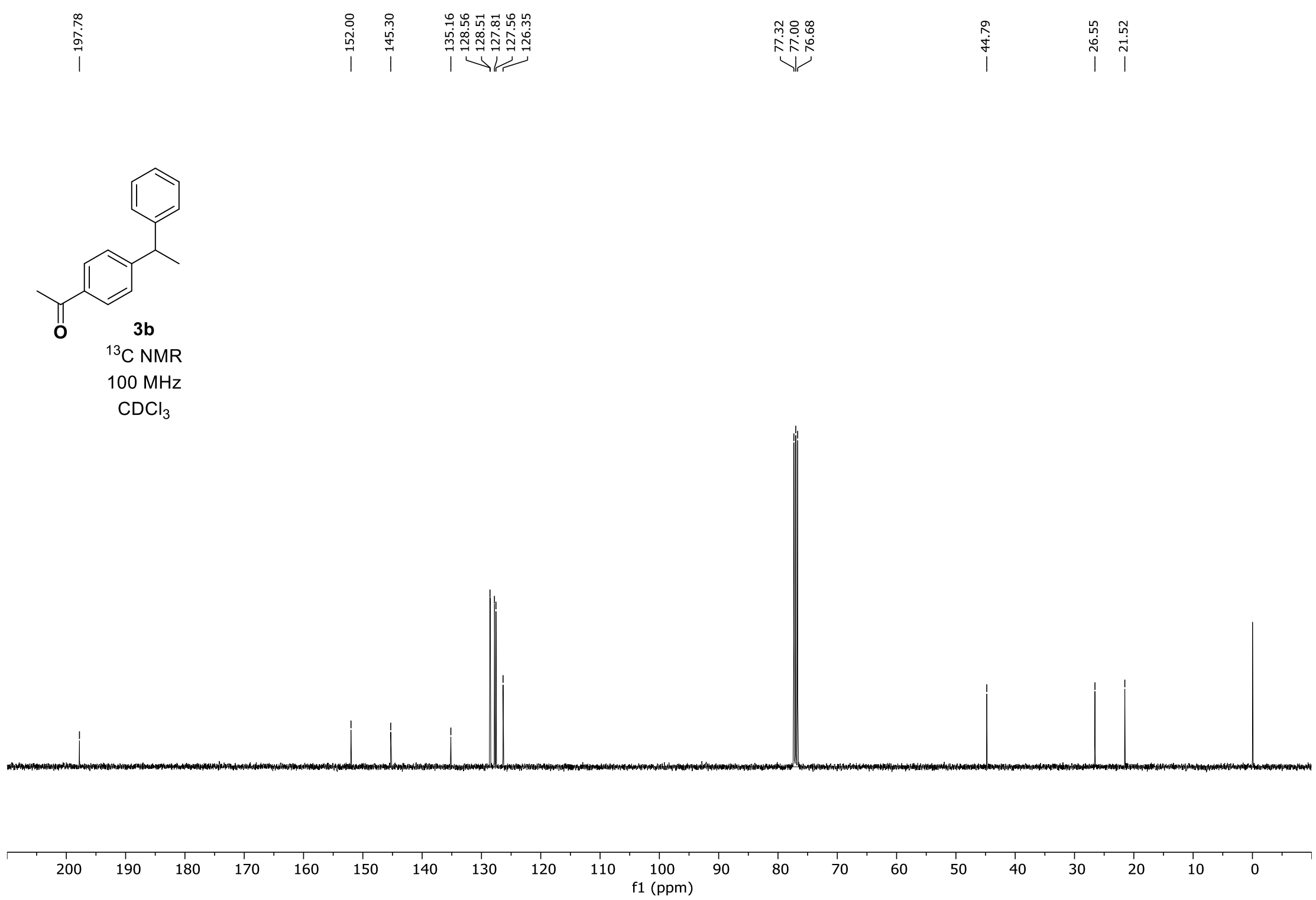

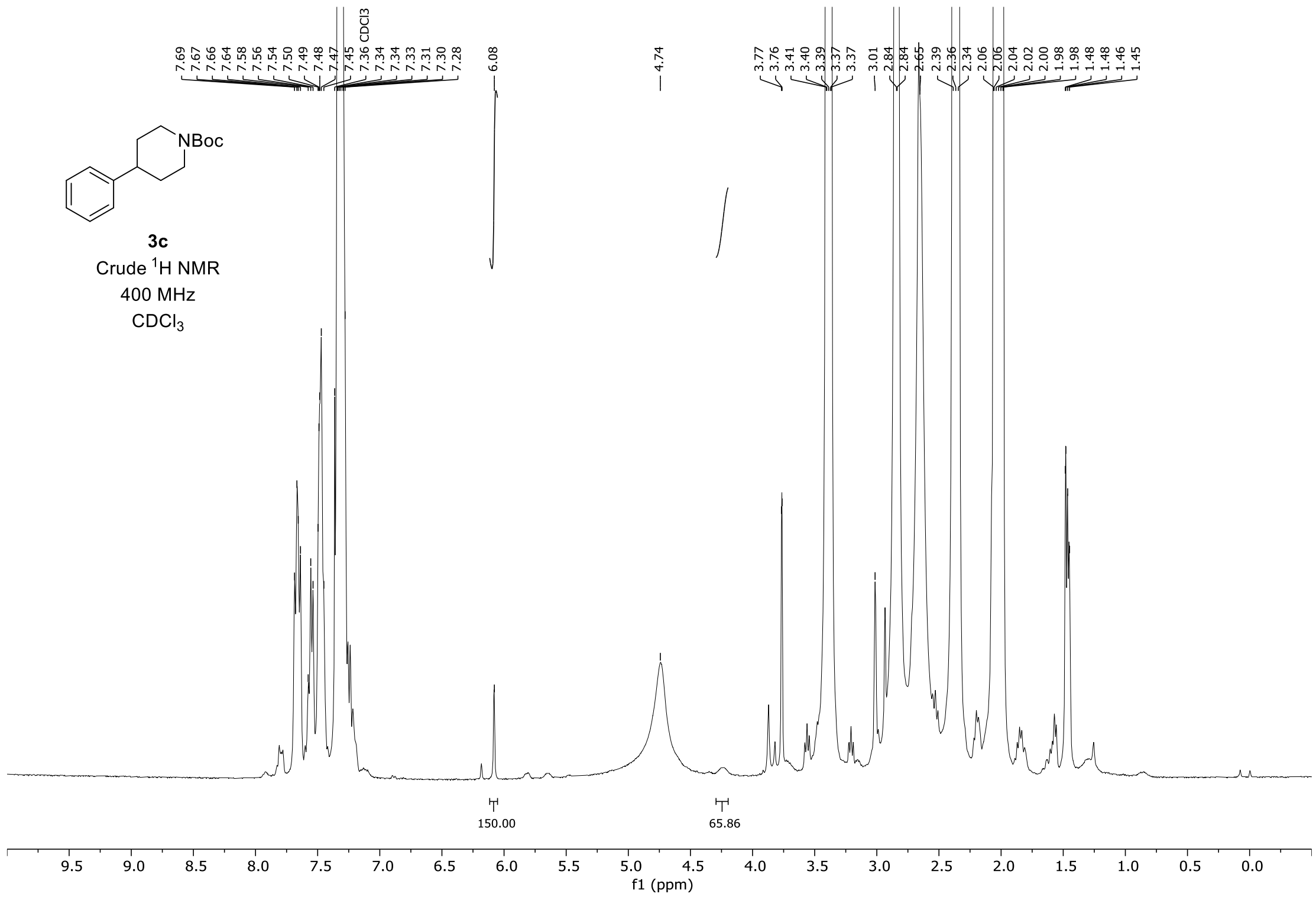

**3c**
Crude $^1$H NMR
400 MHz
$CDCl_3$

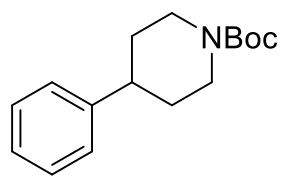


**3c**
$^{1}$H NMR
400 MHz
$CDCl_3$

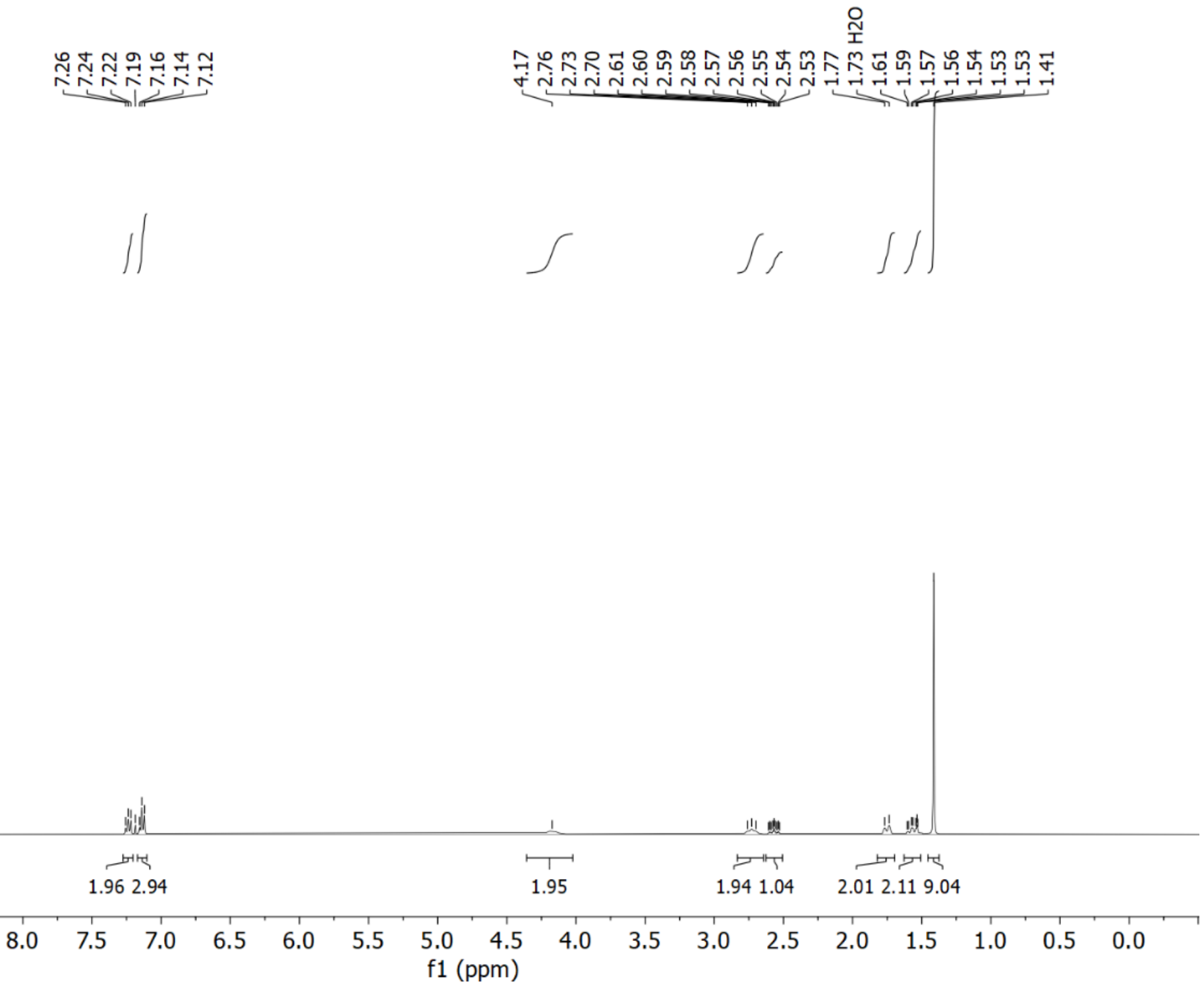


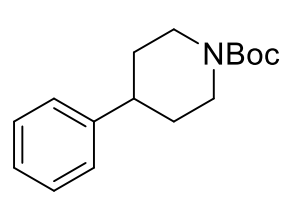


**3c**
$^{13}$C NMR
100 MHz
$CDCl_3$

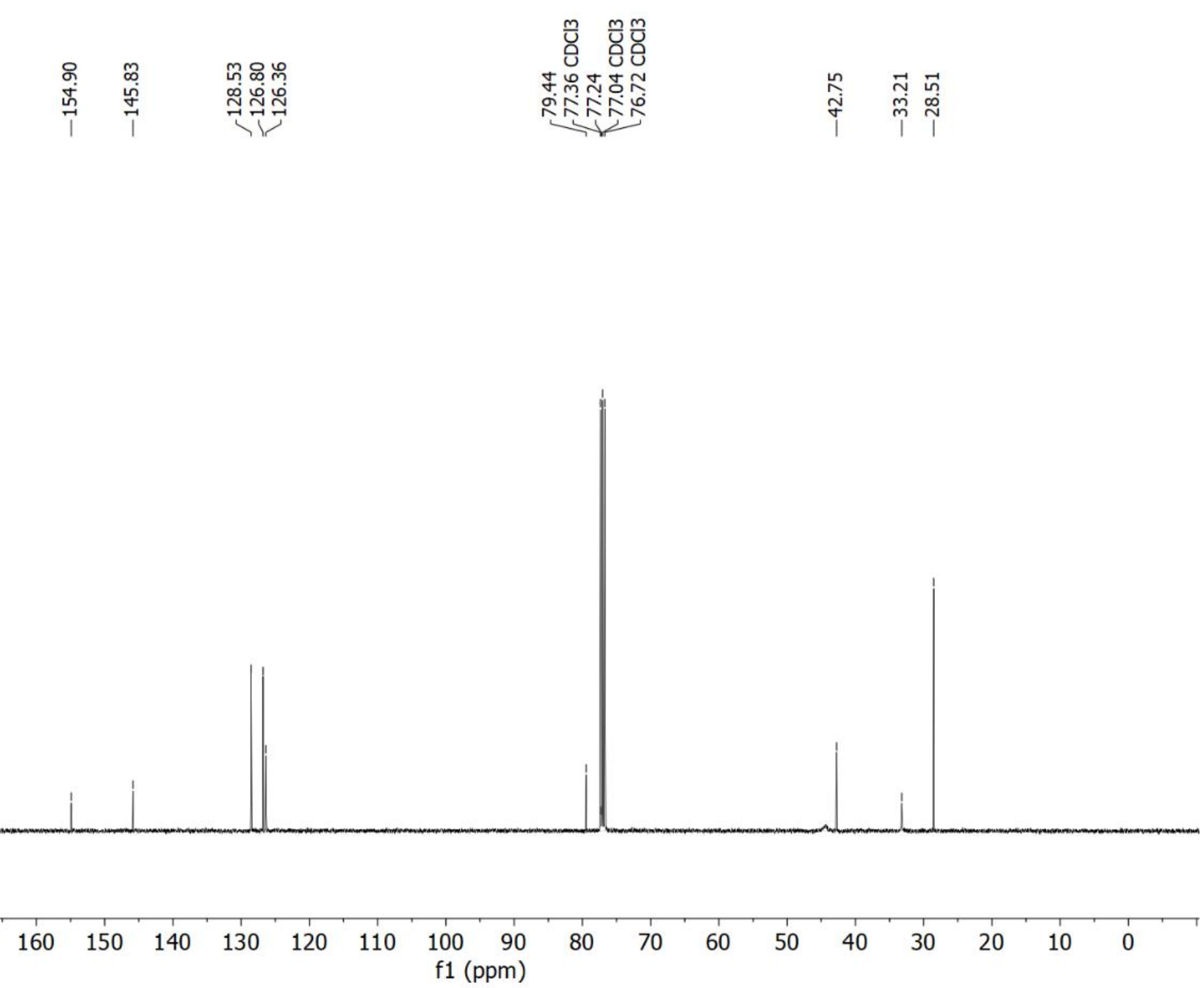

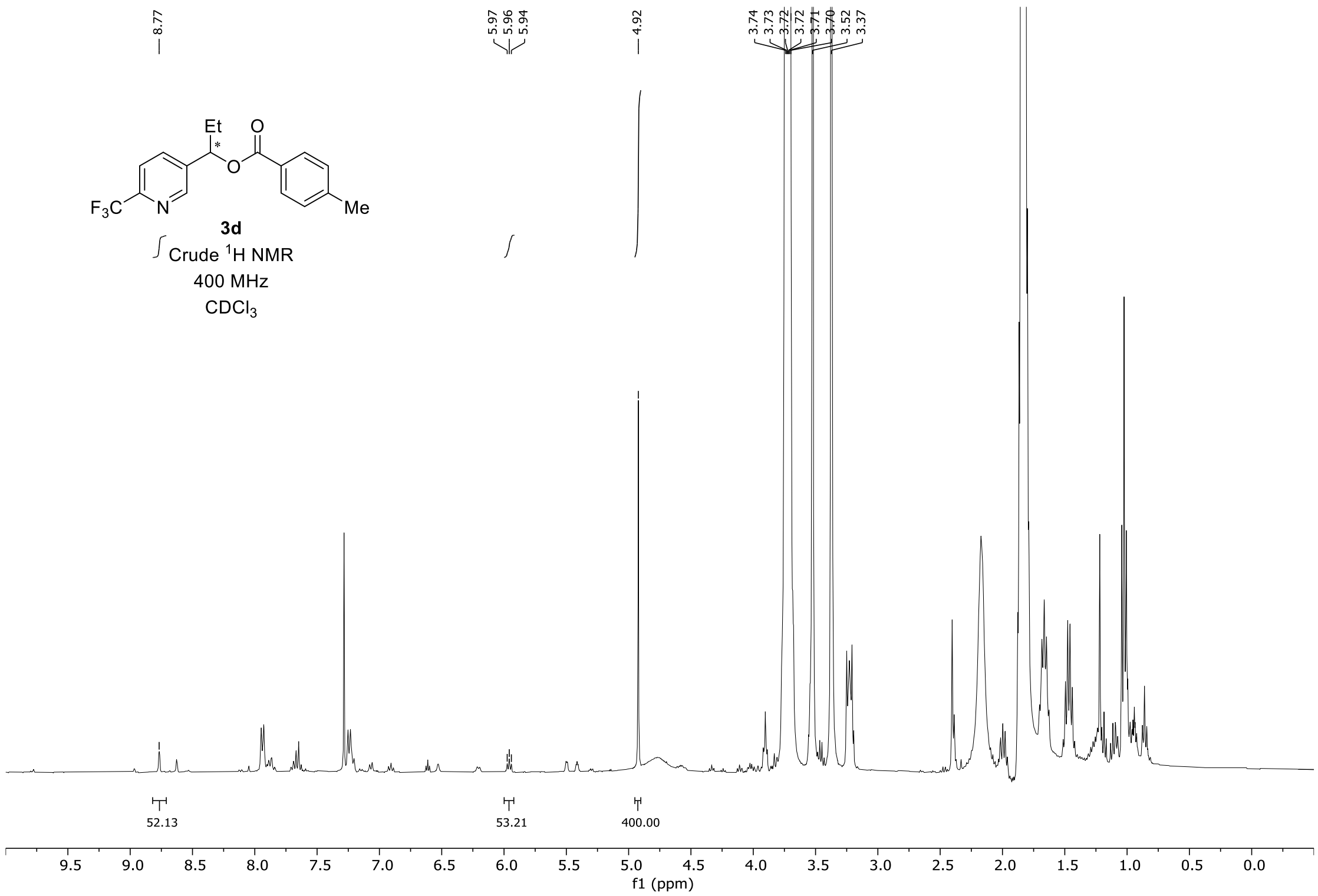
3d
Crude ¹H NMR
400 MHz
CDCl₃
52.13
53.21
400.00
f1 (ppm)

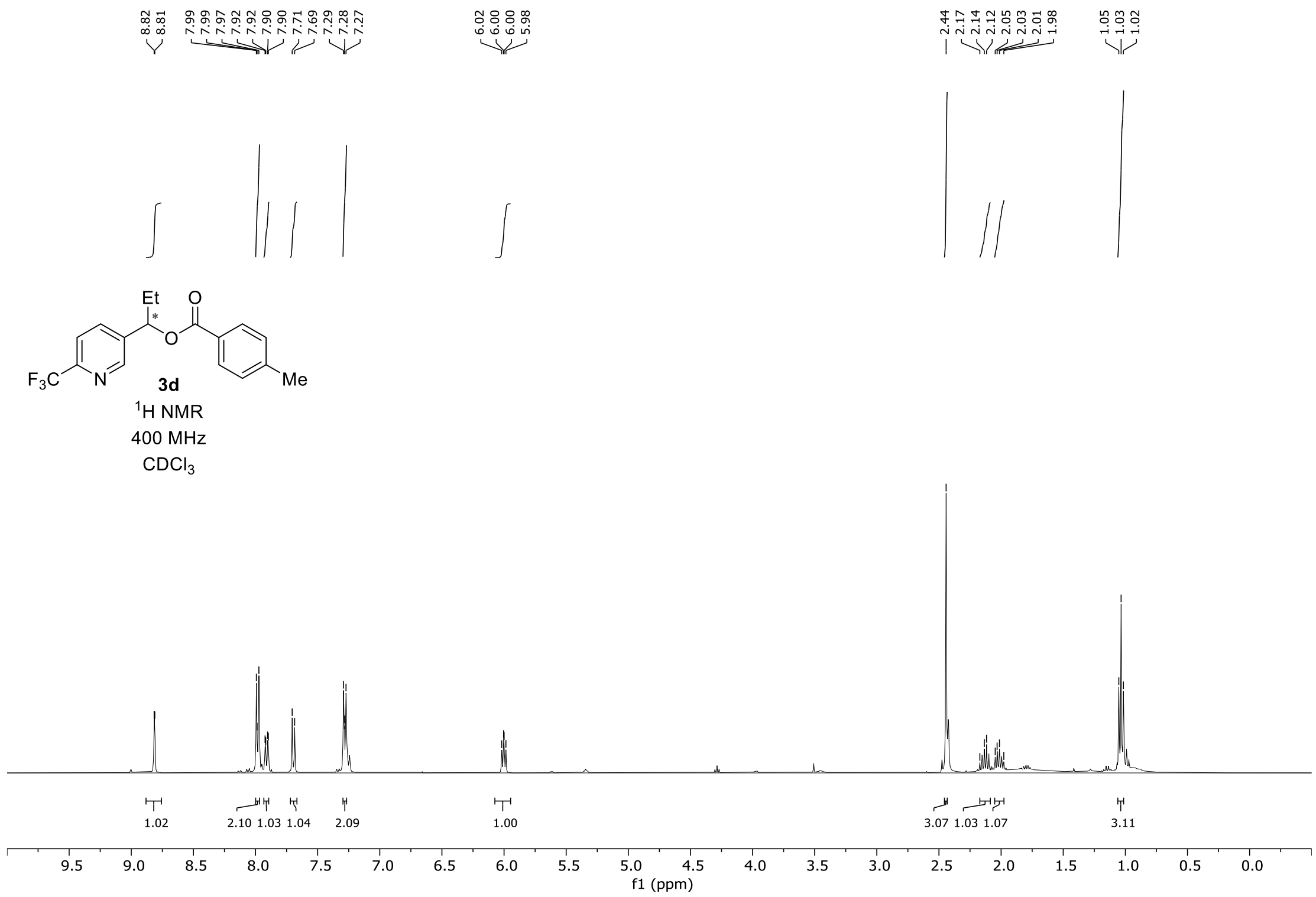
8.82
8.81
7.99
7.99
7.97
7.92
7.92
7.90
7.90
7.71
7.69
7.29
7.28
7.27
6.02
6.00
6.00
5.98
2.44
2.17
2.14
2.12
2.05
2.03
2.01
1.98
1.05
1.03
1.02
Et
O
O
$F_3C$
N
Me
3d
$^1$H NMR
400 MHz
$CDCl_3$
1.02
2.10
1.03
1.04
2.09
1.00
3.07
1.03
1.07
3.11
9.5
9.0
8.5
8.0
7.5
7.0
6.5
6.0
5.5
5.0
4.5
4.0
3.5
3.0
2.5
2.0
1.5
1.0
0.5
0.0
f1 (ppm)

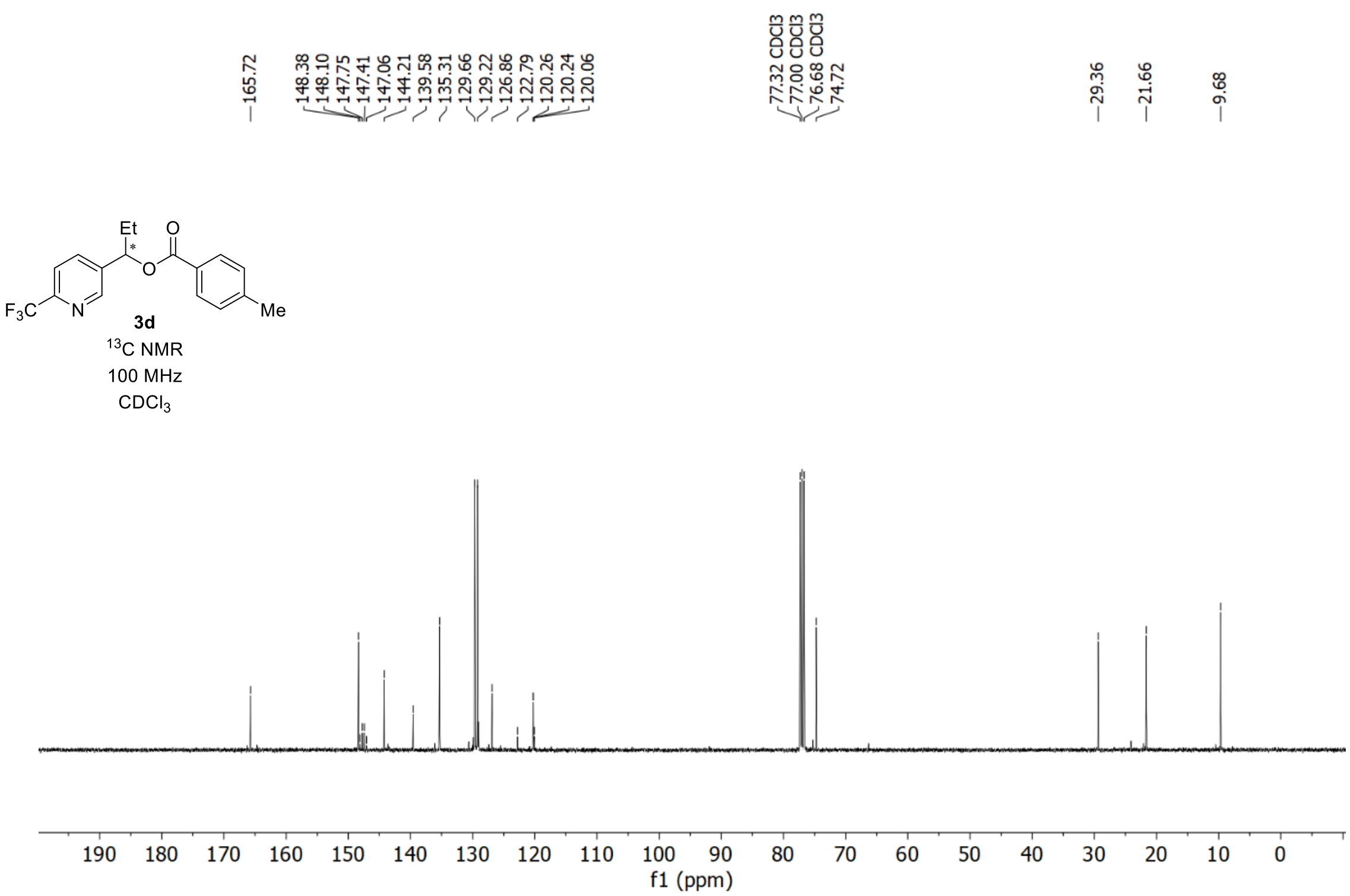
165.72
148.38
148.10
147.75
147.41
147.06
144.21
139.58
135.31
129.66
129.22
126.86
122.79
120.26
120.24
120.06
77.32 CDCl3
77.00 CDCl3
76.68 CDCl3
74.72
29.36
21.66
9.68
Et
O
O
$F_3C$
N
Me
3d
$^{13}$C NMR
100 MHz
$CDCl_3$
190
180
170
160
150
140
130
120
110
100
90
80
70
60
50
40
30
20
10
0
f1 (ppm)
